# Single-Cell RNA-Seq Reveals Chromosomal Instability-Associated Transcriptomic Profiles in Breast Cancer

**DOI:** 10.3390/biomedicines14091902

**Published:** 2026-08-26

**Authors:** María Paula Meléndez-Flórez, Nelson Rangel, Milena Rondón-Lagos, Oscar Ortega-Recalde

**Affiliations:** 1 Departamento de Genética y Ciencias Biomédicas, Facultad de Medicina, Universidad Nacional de Colombia, Bogotá 110231, Colombia; mmelendezf@unal.edu.co; 2 Unidad de Biología Computacional y Analítica de Datos, Biotecgen S.A.S., Bogotá 111111, Colombia; 3 Departamento de Nutrición y Bioquímica, Facultad de Ciencias, Pontificia Universidad Javeriana, Bogotá 110231, Colombia; 4 Escuela de Ciencias Biológicas, Universidad Pedagógica y Tecnológica de Colombia, Tunja 150003, Colombia; sandra.rondon01@uptc.edu.co

**Keywords:** breast cancer, chromosomal instability, clonal heterogeneity, scRNA-seq

## Abstract

**Background/Objectives**: Breast cancer (BC) is the most frequently diagnosed malignancy and a leading cause of cancer-related mortality in women worldwide. This disease is highly heterogeneous and dynamic, and chromosomal instability (CIN) plays a key role in the acquisition of these traits by generating genetic diversity that promotes tumor adaptation and influences therapeutic response and prognosis. Although several methods have been developed to quantify CIN, they are not readily applicable to human tumors and are limited in resolution, hindering a comprehensive understanding of intratumoral heterogeneity. In this study, we aimed to quantify CIN levels and clonal heterogeneity (CH) in HER2-positive (HER2+) and triple-negative (TNBC) breast cancer using single-cell RNA sequencing (scRNA-seq) data. **Methods:** We analyzed publicly available scRNA-seq data from HER2+ and TNBC tumors and non-malignant controls. CIN was scored at single-cell resolution using transcriptomic signatures, clonal heterogeneity was estimated from single-cell diversity metrics, and copy number alterations were inferred computationally. Differential expression and functional enrichment analyses were performed between cells with very low and extreme CIN levels, with key comparisons confirmed at the patient level. **Results**: Our analyses revealed pronounced intra- and intertumoral heterogeneity, with higher CIN levels in TNBC than in HER2+ and control samples. Genes differentially expressed in cells with extreme CIN values were mainly involved in cell division and related processes, and included candidate biomarkers not previously reported in this context. Our findings suggest a positive but statistically non-significant trend was observed between CIN and CH. **Conclusions**: Single-cell approaches such as scRNA-seq provide a powerful framework to elucidate CIN-related mechanisms and to identify potential biomarkers of BC aggressiveness and prognosis, supporting their further application in the study of intratumoral heterogeneity.

## 1. Introduction

Breast cancer (BC) is the most common malignancy and the leading cause of cancer-related mortality among women worldwide, with approximately 2.3 million new cases and 0.67 million deaths reported in 2022 [1]. One of its main biological features is the marked molecular and cellular heterogeneity associated with chromosomal instability (CIN), defined as cell-to-cell variability in chromosome number or structure [2]. CIN represents a major source of genetic diversity that promotes clonal heterogeneity (CH), a fundamental feature of cancer that underlies tumor evolution, phenotypic plasticity, therapy resistance, and poor clinical outcomes [3]. Various methodologies have been developed to assess CIN and CH, including karyotyping, fluorescence in situ hybridization (FISH), expression array-based platforms, and computational inference from bulk omics data. While karyotyping and FISH allow single-cell analysis, they lack the ability to detect small or genome-wide alterations. Conversely, bulk transcriptomic approaches provide only population-level estimates and cannot resolve cell-to-cell variability. Among transcriptomic methods, the CIN70 gene expression signature proposed by Carter et al. [4], together with its subsequent refinements, represents one of the most widely used approaches for inferring CIN and has been primarily validated using bulk transcriptomic datasets. Overall, the absence of a gold-standard methodology constrains the clinical implementation of CIN assessment [5].

Acknowledging these limitations, the recent advent of single-cell RNA sequencing (scRNA-seq) has provided unprecedented resolution to investigate cellular heterogeneity within tumors and their microenvironments. In malignancies such as BC, glioblastoma and non-small cell lung cancer, scRNA-seq has enabled the identification of distinct cellular subpopulations and the characterization of their functional roles in tumor progression, therapeutic response, and tumor evolution [6]. Although transcriptomic CIN signatures, including the original CIN70 signature and its subsequent refinements, have been extensively applied to bulk transcriptomic datasets, their application at single-cell resolution remains limited. Because CIN is inherently defined as cell-to-cell variation in chromosome number or structure, its evaluation particularly benefits from single-cell approaches capable of capturing this biological variability. In contrast to bulk or pseudobulk analyses, which generate an average transcriptional profile across heterogeneous cell populations, scRNA-seq preserves cellular resolution, enabling the identification of individual cells with distinct CIN states, the characterization of rare cellular subpopulations, and a more comprehensive assessment of intratumoral heterogeneity and clonal architecture [7]. These characteristics are particularly relevant for studying the relationship between CIN, CH, and tumor evolution, as chromosomal alterations often emerge and expand within specific cellular subpopulations rather than uniformly across the tumor [3]. Therefore, integrating validated transcriptomic CIN signatures with single-cell transcriptomics represents a complementary strategy that combines the robustness of established CIN signatures with the high-resolution profiling afforded by scRNA-seq, providing new opportunities to investigate the contribution of CIN to tumor evolution, therapeutic resistance, and phenotypic plasticity [3,7].

This study aimed to quantify CIN levels and CH in HER2-positive (HER2+) and triple-negative BC (TNBC) subtypes using scRNA-seq data. These subtypes exhibit greater aggressiveness than luminal tumors and are associated with increased rates of relapse and metastasis [8]. The results revealed marked intra- and intertumoral heterogeneity, with significantly higher CIN levels observed in TNBC compared with HER2+ and control samples. Differentially expressed genes in cells exhibiting extreme CIN values were predominantly associated with cell division and related biological processes and included previously unreported biomarker candidates. Overall, our findings highlight the potential association between CIN and CH, underscoring the value of single-cell for elucidating CIN-related mechanisms in BC. However, given the limited number of samples analyzed, these findings should be interpreted with caution. Future studies integrating transcriptomic CIN signatures with direct cytogenetic and genomic measurements in larger patient cohorts will be essential to validate these observations and to determine their biological and clinical significance.

## 2. Materials and Methods

### 2.1. scRNA-Seq Data Collection and Pre-Processing

This study analyzed scRNA-seq data from TNBC and HER2+ BC patients, as well as healthy individuals used as the control group. Tumor data were obtained from the study GSE176078 [9,10,11], which includes samples from seven TNBC patients and four HER2+ BC patients. Control samples were derived from three individuals who underwent reduction mammoplasty, available under accession GSE180878 [12]. Raw sequencing data were retrieved from the Gene Expression Omnibus (GEO) and the European Genome-Phenome Archive (EGA). None of the patients received adjuvant therapy at the time of sample collection, thereby minimizing potential confounding effects related to treatment-induced transcriptional changes.

For clarity and ease of reference in this study, all samples have been assigned consecutive codes: TNBC samples are labeled as T1–T7, HER2+ samples as H1–H4, and control samples as C1–C3. The original sample identifiers are provided in Supplementary Table S1. Sequencing of all samples was conducted using the 10x Genomics platform, and raw reads were processed using the Cell Ranger (v.7.2.0) pipeline [13]. Alignment was performed against the GRCh38 human reference genome, yielding gene expression count matrices for each sample. Subsequent quality control and filtering steps were conducted in RStudio (v4.2.1) using the scDblFinder (v.1.24.0) and Seurat (v5) packages [14,15]. Samples with fewer than 500 detected cells were excluded. At the cellular level, doublets and low-quality cells defined as those expressing fewer than 200 genes, fewer than 500 Unique Molecular Identifiers (UMIs), or containing ≥20% mitochondrial gene content were also removed from the dataset. After filtering, the expression data were log-normalized to account for differences in sequencing depth. To mitigate batch effects between the two independent datasets, integration and dimensionality reduction were performed using Seurat. Specifically, the FindIntegrationAnchors and IntegrateData functions were applied to identify mutual nearest neighbors and harmonize gene expression profiles across cohorts, facilitating a unified downstream analysis.

### 2.2. Determination of Major Cell Lineages and Copy Number Inference of RNA-Seq Data

Gene expression normalization was performed using the NormalizeData and ScaleData functions of the Seurat package. After normalization, the FindVariableFeatures function was applied to identify the 1500 most variable genes across the dataset. To reduce dimensionality and facilitate efficient downstream analysis, principal component analysis (PCA) was performed, followed by uniform manifold approximation and projection (UMAP) for visualization of the cellular landscape. To identify transcriptionally distinct cell clusters, the Seurat functions FindNeighbors and FindClusters were employed, generating unsupervised groupings based on PCA-reduced dimensions. Annotation of resulting clusters was performed by examining the expression of well-established cell type-specific marker genes. To enhance and validate cluster classification, we additionally employed the Single Cell Experiment v1.32.0 and SingleR v.2.12.0 packages, which leverage reference-based annotation strategies to assign cell subpopulations [16,17].

Following cell type annotation, copy number variants (CNV) were inferred at the single-cell level using the inferCNV v.1.26.0 package in RStudio (v4.2.1) [18]. Annotated non-epithelial cells present in the same samples—B cells, CD4^+^ and CD8^+^ T cells, endothelial cells, and fibroblasts—were specified as the reference (diploid) group, while epithelial cells were evaluated as the observation set. This use of infiltrating non-malignant immune and stromal cells as the reference baseline follows the standard approach for single-cell CNV inference [19,20]. For CNV inference and subsequent epithelial-level analyses, a minimum of 100 annotated epithelial cells per patient was required. Patients H2 and T1, which yielded 7 and 53 epithelial cells respectively after annotation, fell below this threshold and were excluded from these analyses; both samples were nonetheless retained through quality control and dataset integration.

### 2.3. Assessment of mRNA Expression Signatures in scRNA-Seq Data

CIN was assessed by calculating the expression-based scores of two previously described gene expression-based signatures: CIN70, which comprises 70 genes involved in cell cycle regulation and mitotic processes, and CIN17, a 17-gene signature associated with chromosome maintenance, mitotic control, and transcriptional features of chromosomal instability. The overall CIN profile was determined by evaluating the individual and combined expression patterns of both signatures [4,19]. CIN levels from these expression signatures were quantified using the R package UCell v.2.14.0, which computes gene activity in scRNA-seq data based on the Mann–Whitney U statistic [20].

Following criteria established in previous studies, CIN levels were classified into four categories: 0–25% (very low CIN), 26–50% (low CIN), 51–75% (high CIN), and 76–100% (extreme CIN) [4,21,22].

### 2.4. Differential Expression and Functional Enrichment Analyses

Differentially expressed genes (DEGs) were primarily identified at the single-cell level using the FindMarkers function from the Seurat package with the MAST statistical framework [22], which applies a hurdle model tailored to single-cell count data. *p*-values were adjusted for multiple testing using the Bonferroni method. To evaluate the robustness of these single-cell findings and explicitly account for patient-specific effects (inter-patient variability), a complementary pseudobulk differential expression analysis was performed. For this approach, single-cell raw counts were aggregated at the patient level within each CIN category and analyzed using the DESeq2 package [23], which identified 516 significant DEGs (adjusted *p* < 0.05). Functional enrichment analysis for both single-cell and pseudobulk DEG sets was carried out using the enrichGO function from the clusterProfiler package, evaluating three Gene Ontology (GO) categories: Biological Process (BP), Cellular Component (CC), and Molecular Function (MF) [24,25,26]. Statistical significance for GO terms was defined as *p* < 0.05 and adjusted *p* < 0.05, using the Benjamini–Hochberg False Discovery Rate (FDR) method. Genes included in the CIN87 signature were removed from the GO analyses.

### 2.5. Statistical Analyses

A descriptive statistical approach was applied to characterize the distribution and frequency of the studied variables, including clinicopathological features available, CIN, and CH in patients diagnosed with HER2+ and TNBC. To explore potential associations between CIN, CH levels and the clinicopathological variables, statistical tests were selected according to data type and distribution: the Mann–Whitney U test was used for comparing CIN and CH levels between two groups, the Kruskal–Wallis test was applied for comparisons across multiple molecular subtypes, and Spearman’s rank correlation was employed to assess relationships between continuous variables. For categorical variables such as subtype and lymphovascular invasion, the Chi-square test was used, and Cramer’s V coefficient was calculated to evaluate the strength of association. A significance threshold of *p* < 0.05 was adopted for all analyses.

To further investigate molecular–clinical relationships, a correlation analysis was conducted to evaluate the association between differentially expressed genes and clinicopathological variables. This analysis was restricted to tumor samples, excluding controls, and performed independently for each gene–clinical parameter pair. Spearman’s rank correlation coefficient was employed to quantify the strength of these associations. Genes were considered significantly correlated with specific clinical variables when they exhibited a *p*-value < 0.05 and a correlation coefficient (ρ) ≥ 0.7.

### 2.6. Software and Packages

Bioinformatic analyses were executed in R (v4.2.1). Single-cell processing, doublet detection, and batch integration were conducted using Seurat (v5.5.1), scDblFinder (v1.26.7), and harmony (v2.0.5). Cell type annotation was performed using SingleR (v2.14.1) with the Human Primary Cell Atlas reference (celldex v1.22.0, HPCA 26 Febuary 2024). Copy number variations were inferred using inferCNV (v1.18.0) applying 10x Genomics defaults (cutoff = 0.1, denoise = TRUE) against control non-epithelial cells as baseline. Single-cell CIN scoring was calculated using UCell (v2.16.0) and CINSignatureQuantification (v1.2.0). Differential expression was performed using MAST (v1.38.0) for single-cell data and DESeq2 (v1.25.0) for pseudobulk sensitivity analysis, followed by clusterProfiler (v4.20.0) for Gene Ontology enrichment. General data manipulation, statistical testing, and visualization were supported by tidyverse (v2.0.0), ggplot2 (v4.0.3), ggpubr (1.0.0), patchwork (1.3.2), and associated R packages.

## 3. Results

### 3.1. Integration of scRNA-Seq Reveals Distinct Cell Populations and Tumor Heterogeneity

scRNA-seq data were obtained from eleven (11) breast tumor samples from treatment-naïve patients, including four (4) HER2+ and seven (7) TNBC cases. In addition, three control samples were included, derived from individuals who underwent reduction mammoplasty. Table 1 summarizes the general clinical and demographic characteristics of the samples included in the study.

**Figure 1:**
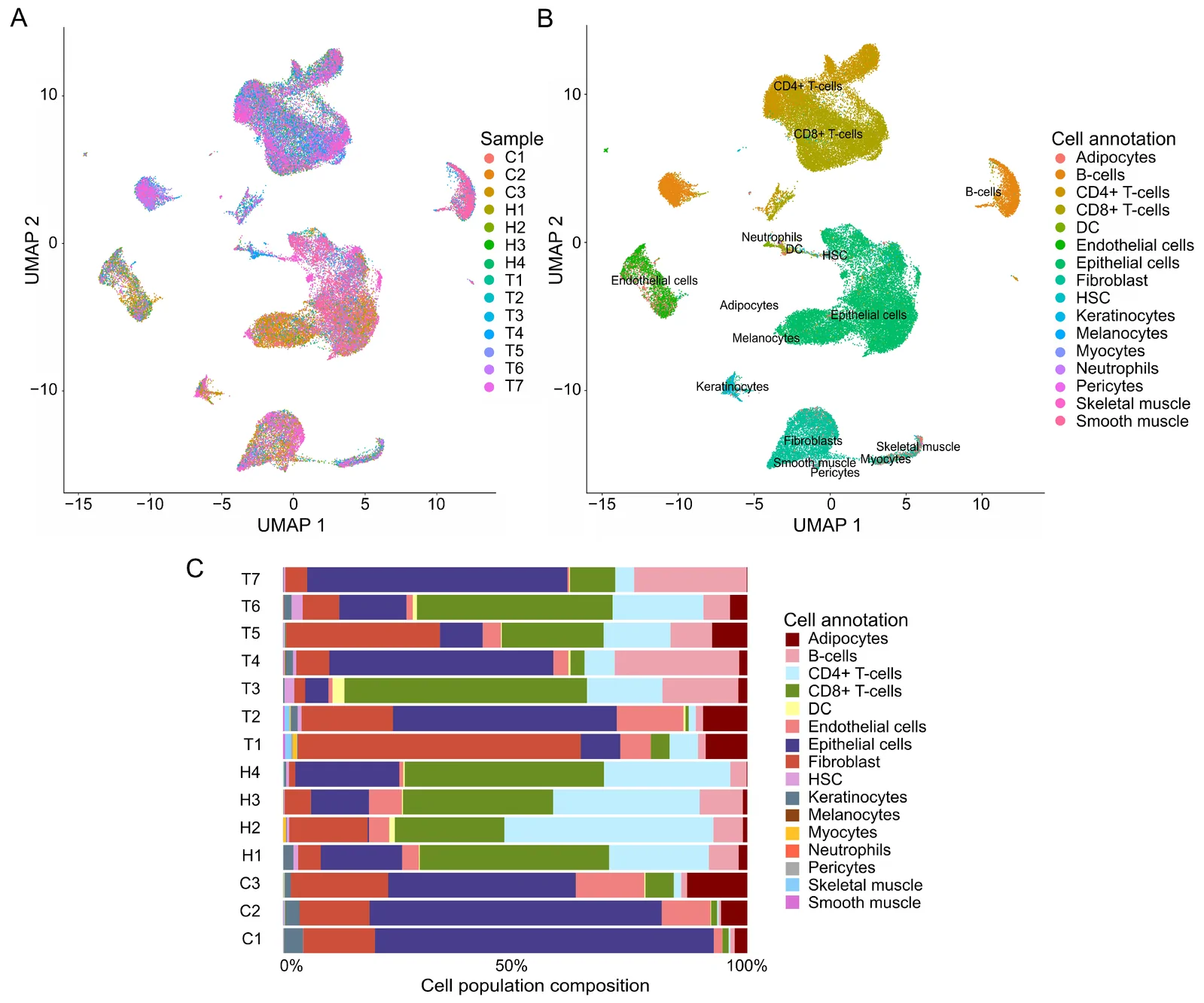
Tumor cell composition UMAP. UMAP visualization of the 72,281 integrated single cells from BC patient samples and healthy controls. (**A**) Integration of the 14 samples analyzed, including eleven BC patients (four HER2+ and seven TNBC) and three control individuals. The UMAP demonstrates effective batch-effect correction, resulting in homogeneous cell distribution independent of sample origin. Colors indicate individual samples. (**B**) Annotation of cellular clusters based on cell type-specific transcriptomic signatures. Multiple distinct cell populations were identified, including dendritic cells (DC), hematopoietic stem cells (HSC), T cells, B cells, epithelial cells, and others. (**C**) Distribution of different cell types across HER2+ and TNBC patients and control individuals. Each bar represents the proportion of annotated cell types per sample. Corresponding colors are shown on the right.

Before data integration, a rigorous quality control process was applied to ensure accuracy. Standard scRNA-seq metrics were used for filtering, including the number of unique molecular identifiers (UMIs), the number of genes detected per cell, and the percentage of mitochondrial gene expression. After filtering, the dataset was reduced from 84.081 captured cells to 72,281 high-quality cells suitable for downstream analyses with an average of 5171 cells per sample (Ranges 876–13.149) (Supplementary Table S2). After quality filtering, the data was integrated to correct batch effects and facilitate the quantification and classification of cells across all tumor and control samples (Figure 1A). This integration simplified the process of identifying the number and type of cells in the tested samples and eliminated the batch effect. Cell annotation was then performed using the SingleR algorithm in combination with reference transcriptomic signatures specific to each cell type. Sixteen (16) distinct cell types were identified, including: adipocytes, B cells, CD4^+^ and CD8^+^ T cells, dendritic cells, endothelial cells, epithelial cells, fibroblasts, hematopoietic stem cells (HSCs), keratinocytes, melanocytes, myocytes, neutrophils, pericytes, skeletal muscle cells, and smooth muscle cells. UMAP analysis highlights the relative abundance of each cell type across individuals and clearly separates the annotated cell subtypes (Figure 1B). Moreover, the visualization comparison allows comparison of cellular heterogeneity between tumor subtypes and controls (Figure 1C), revealing a higher proportion of epithelial cells in control samples, whereas patient samples were enriched in immune cells. Once cell populations were identified, epithelial cells were subset and included for further analysis.

### 3.2. CIN87 Uncovers a TNBC Specific High Instability Profile Distinct from HER2+ and Control Tissues

**Figure 2:**
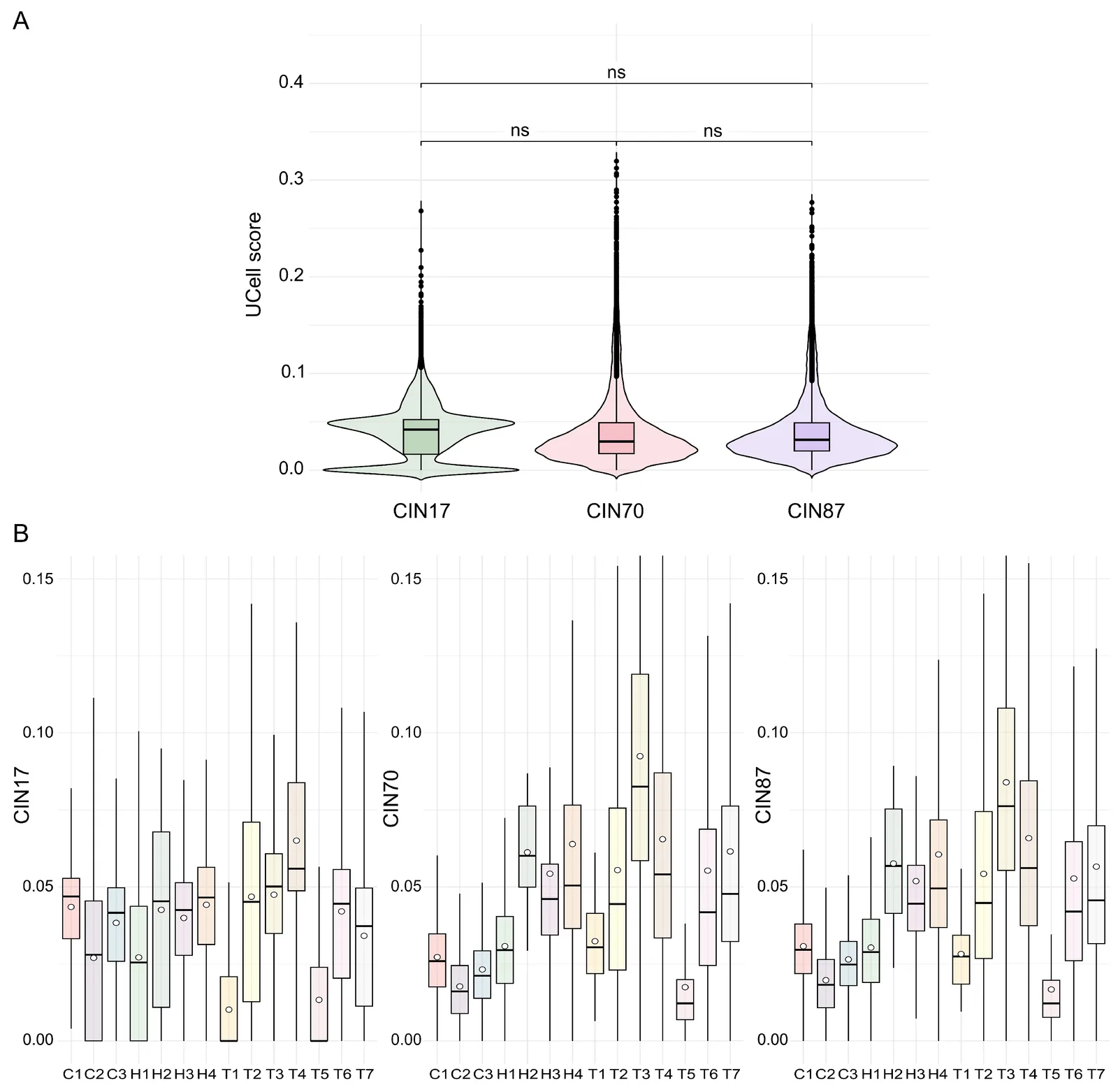
Assessment of CIN expression signatures across BC patients and tumor types. CIN was evaluated using three gene expression signatures: CIN17, CIN70, and CIN87. (**A**) Comparative global analysis of CIN17, CIN70, and CIN87 scores across all single cells. The Y-axis (0–0.40) reflects the full dynamic range, accommodating extreme single-cell outliers and statistical annotations. Statistical comparisons were evaluated at the patient level using mean scores per signature. No statistically significant (ns) differences were observed among the three signatures after multiple testing correction (*p* > 0.05). (**B**) Distribution of CIN17, CIN70, and CIN87 scores across individual patients and single cells. The Y-axis for Panel B is focused (0–0.15) to optimize visual resolution of interquartile ranges and median differences across individual patient samples. Although the three signatures showed similar overall CIN-associated expression patterns, CIN17 displayed greater overlap among samples, whereas CIN70 and CIN87 captured additional variation in CIN-related expression profiles. CIN87, generated by integrating the gene sets from CIN70 and CIN17, provides a combined representation of CIN-associated gene expression patterns and was selected for subsequent analyses of chromosomal instability heterogeneity in the dataset. Statistical comparisons were performed using the Friedman test followed by paired Wilcoxon post-hoc tests with Holm adjustment or Benjamini–Hochberg adjustment for multiple comparisons. No pairwise comparisons remained statistically significant after correction.

Two previously validated gene expression signatures, CIN70 and CIN17, were initially applied to independently assess CIN levels in BC patients and healthy controls at single-cell level. Comparative analysis at the patient level, treating each tumor sample as an independent biological replicate, showed no statistically significant differences among CIN17, CIN70, and the composite CIN87 signature (CIN17 vs. CIN70: *p* = 0.126, CIN17 vs. CIN87 *p* = 0.126; CIN70 vs. CIN87 = 0.126, Figure 2A). These results indicate that the overall CIN-associated gene expression patterns are comparable across signatures when evaluated across biological replicates. However, single-cell analyses revealed that the three signatures capture distinct patterns of CIN-associated gene expression and heterogeneity among individual tumor cells (Figure 2B), showing statistical significances when comparing groups at the cell level (*p* < 0.001).

Because CIN87 integrates the gene sets represented by CIN70 and CIN17, it provides a combined measure of CIN-associated expression patterns and may enable a more comprehensive characterization of chromosomal instability heterogeneity at the single-cell level. Therefore, CIN87 was selected for subsequent analyses of CIN heterogeneity in our dataset, while statistical comparisons were performed using patient-level measurements to ensure appropriate biological replication.

**Figure 3:**
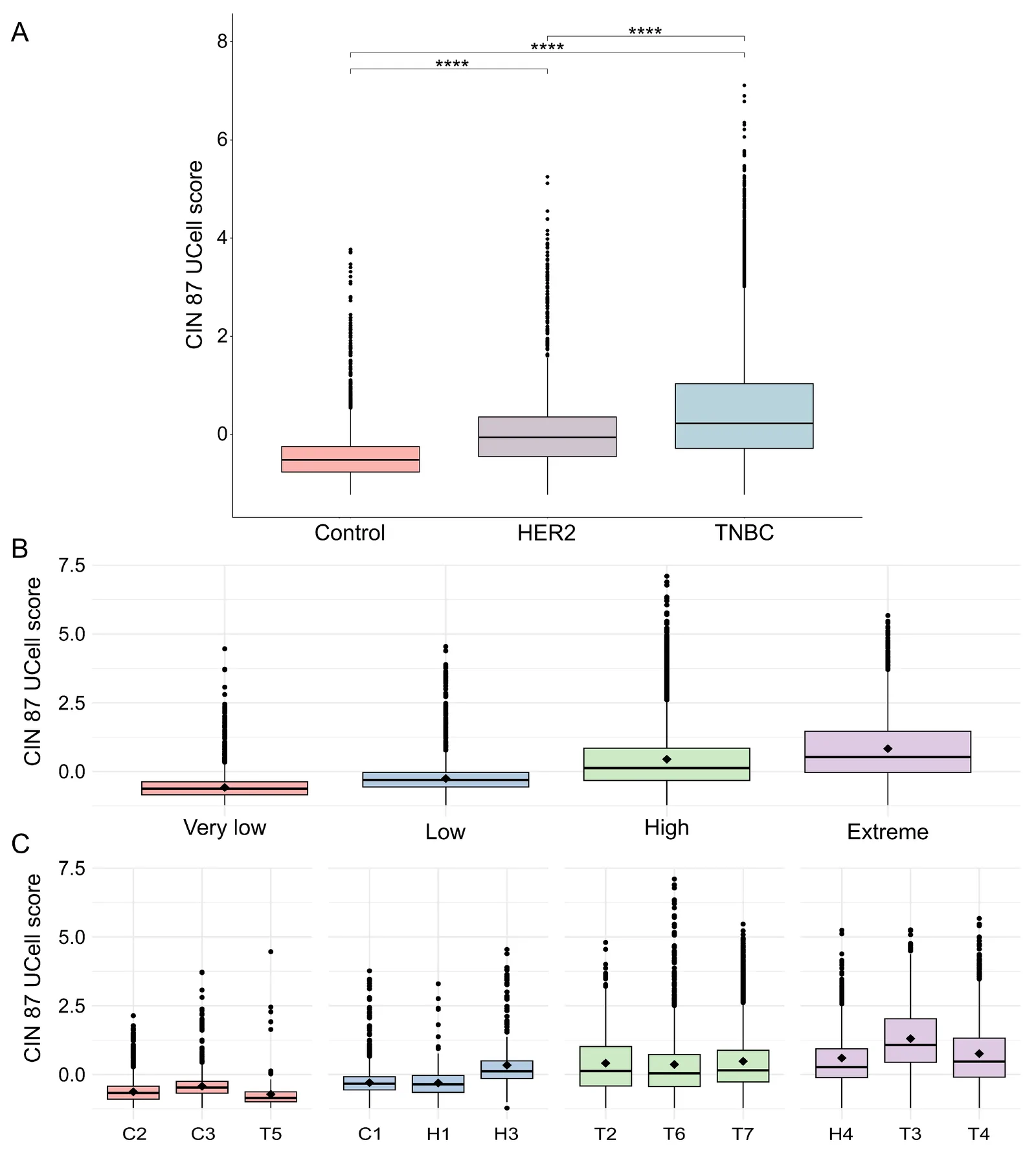
CIN87-based assessment of chromosomal instability across breast cancer subtypes. CIN levels were evaluated using the normalized CIN87 UCell enrichment score (CIN87_UCell_Normalized). All panels display the same normalized metric to ensure consistent interpretation across analyses. (**A**) Boxplots showing normalized CIN87 scores across control, HER2+, and TNBC groups. Statistical comparisons were performed using Dunn’s test with Benjamini–Hochberg adjustment, showing significant differences between Control and HER2+ groups (*p* = 5.93 × 10^−173^), Control and TNBC groups (*p* < 2.2 × 10^−16^), and HER2+ and TNBC groups (*p* = 2.00 × 10^−28^). **** *p* < 0.0001. (**B**) Distribution of normalized CIN87 scores across predefined CIN categories (very low, low, high, and extreme) for each molecular subtype. (**C**) Individual-level CIN category assignments highlighting subtype-specific CIN patterns, with most TNBC samples classified within high or extreme CIN categories, HER2+ samples showing broader CIN distributions, and control samples predominantly classified within very low and low CIN categories.

Expression analysis using the normalized CIN87 UCell enrichment score (CIN87_UCell_Normalized) revealed differences in CIN-associated gene expression levels across breast cancer molecular subtypes and control individuals (Figure 3A). All panels were updated to display the same normalized CIN87 metric, allowing direct comparison across analyses. Statistical comparisons based on normalized CIN87 scores were performed using Dunn’s test with Benjamini–Hochberg adjustment, revealing significant differences between control, HER2+, and TNBC groups (Control vs. HER2+: *p* = 5.93 × 10^−173^; Control vs. TNBC: *p* < 2.2 × 10^−16^; HER2+ vs. TNBC: *p* = 2.00 × 10^−28^). TNBC tumors exhibited the highest CIN87_UCell_Normalized scores among the analyzed groups.

Stratification based on CIN categories further highlighted differences in the distribution of CIN87-associated states among molecular subtypes, with most TNBC samples clustering within the high or extreme CIN categories, whereas HER2+ cases showed a broader distribution across CIN categories. Control samples were predominantly classified within the lowest CIN categories (Figure 3B). Substantial variability was observed among individual samples, indicating heterogeneity in CIN-associated expression patterns within and between molecular subtypes. Notably, patient T5 displayed considerably lower CIN87_UCell_Normalized levels than other TNBC and HER2+ cases, showing a profile comparable to control samples (Figure 3C). Together, these findings indicate that the normalized CIN87 signature captures differences in CIN-associated gene expression patterns across breast cancer subtypes while revealing heterogeneity among individual tumors.

### 3.3. CIN Drives CH in BC Exhibiting Distinct Patterns in TNBC and HER2+ Subtypes

After applying the CIN87 expression signature to evaluate CIN, we aimed to analyze its association with CH, copy number variations (CNVs), and other key clinical and pathological features. CH, a key aspect of tumor biology that reflects the coexistence of multiple genetically distinct subpopulations within a single tumor, was quantified using two diversity metrics: the Shannon Diversity Index (SDI) and the True Diversity Index (DV). These indices account for both, the richness and relative abundance of cellular clones, and were calculated following previously established methodologies [27,28].

BC patients exhibited markedly higher CH compared to healthy controls when evaluated using two diversity metrics: the Shannon Diversity Index (SDI) and true diversity (DV). SDI mean values were 1.25 in HER2+ tumors and 1.33 in TNBC tumors, compared with 0.73 in controls, indicating an increase in clonal diversity in tumor samples. True diversity (DV) showed mean values of 3.56 in HER2+, 4.14 in TNBC, and 2.09 in controls. The fold-change estimates refer specifically to DV comparisons between each tumor subtype and the control group, with HER2+ and TNBC tumors exhibiting approximately 1.71-fold and 1.98-fold higher diversity, respectively (Table S3). Thus, both SDI and DV consistently support increased CH in tumors relative to controls, although statistical significance was not reached in the non-parametric tests. A consistent trend toward increased clonal complexity was nevertheless observed, particularly in TNBC samples.

**Figure 4:**
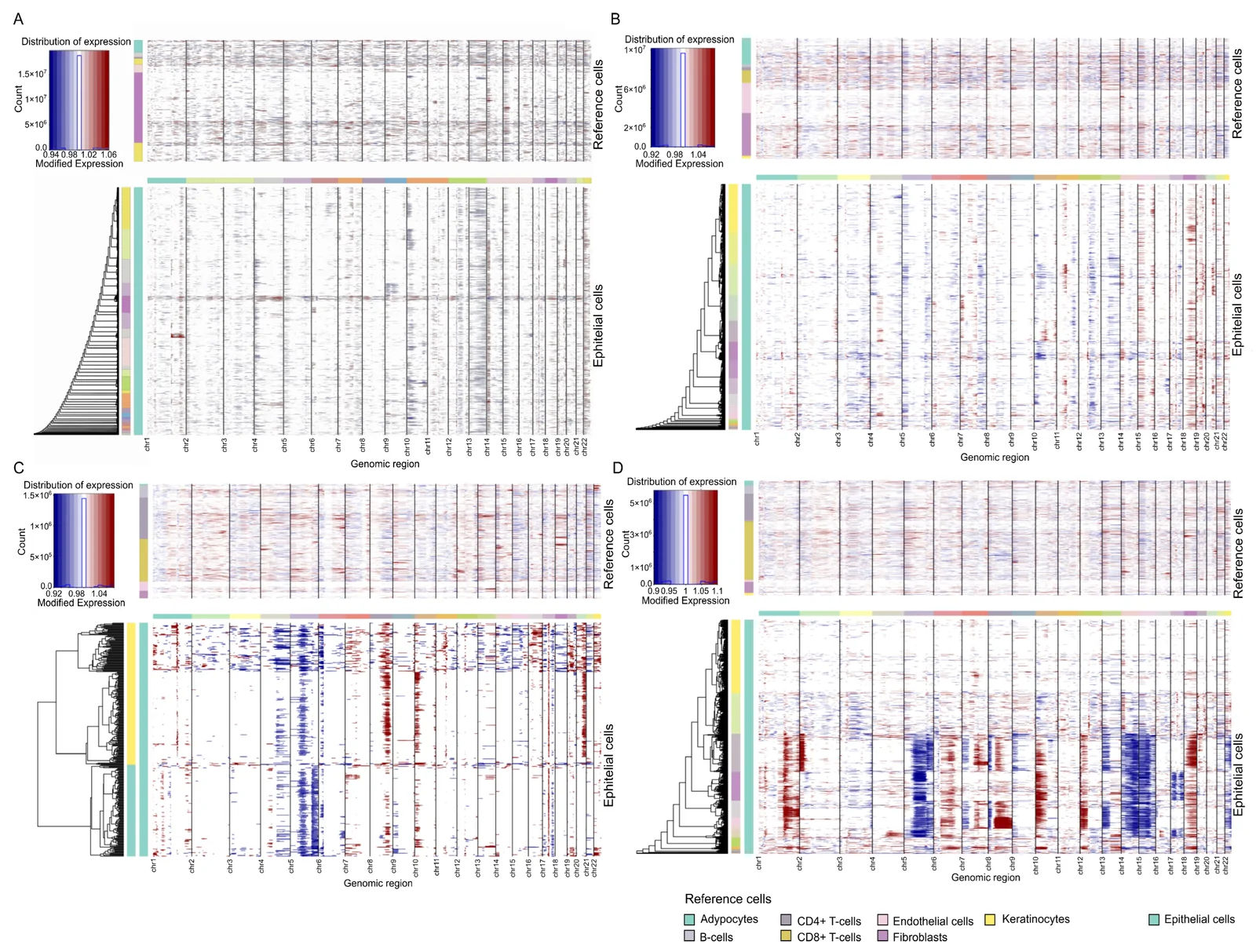
Correlation between expression signatures, CNVs and cell diversity. (**A**,**B**) Heatmaps of inferred CNV profiles from two control samples showing stable genomic profiles without significant chromosomal gains or losses. (**C**,**D**) Heatmaps of CNV profiles from TNBC and a HER2+ samples, highlighting recurrent alterations, including 5q loss in TNBC (T6) and 17p loss in HER2+ (H3) as well as additional subtype-specific gains and losses.

To further investigate the genomic mechanisms underlying CIN, CNVs were analyzed, revealing both common and subtype-specific structural alterations. Samples from patients H2 and T1 were excluded prior to CNV analyses as they failed to meet our quality threshold of a minimum of 100 epithelial cells per patient (patient H2 yielded 7 cells; patient T1 yielded 53 cells). In TNBC patients, recurrent loss of the 5q chromosomal region was frequently detected; for example, patient T6 exhibited gains in 1q and 18q, together with losses in 5q, 14q, and 15q. In the HER2+ group, approximately 86.9% of patients showed loss of 17p, and 31% displayed loss of 5q. Patient H3 presented losses in 4q, 5q, and 17p, along with gains in 8q and 20q (Figure 4C,D). In contrast, control individuals exhibited no detectable structural alterations, reflecting a genomically stable profile (Figure 4A,B).

In order to further explore the relationships among CIN, CH, and key clinical variables, including age, Ki67 expression, molecular subtype, and lymphovascular invasion, correlation analyses were conducted. Spearman’s correlation coefficient was applied to continuous variables, and Cramer’s V test was used for categorical variables. A positive association between CIN and CH was observed in both tumor subtypes, supporting the interconnection between CIN and clonal diversity (Supplementary Table S4). It should be highlighted that given the small number of samples this correlation should be interpreted as suggestive rather than conclusive. No strong correlations were detected between CIN or CH with other clinical parameters analyzed. Comparative analyses between HER2+ and TNBC tumors revealed higher CIN, CH assessed by DV, and Ki67 expression levels in TNBC, consistent with its more aggressive molecular profile. Although these differences did not reach statistical significance, the observed trends were biologically coherent and suggestive of distinct underlying molecular dynamics between subtypes (Supplementary Table S5).

When lymphovascular invasion was evaluated independently, no major differences were observed. However, when the molecular subtype was considered in combination with lymphovascular invasion, distinct patterns emerged for CIN, CH assessed by DV, and Ki67. Specifically, TNBC cases with lymphovascular invasion displayed higher values across all three parameters compared to HER2+ tumors (Supplementary Table S5). Although these differences did not reach statistical significance, the observed tendencies are consistent with the notion of greater biological complexity and aggressiveness in TNBC within invasive contexts.

### 3.4. Differential Gene Expression and Functional Enrichment Reveal Mitotic Dysregulation in Cells with Extreme CIN

In order to investigate the transcriptional landscape associated with CIN, differential gene expression analysis was performed. Epithelial cells were stratified into two extreme CIN87-based groups: very low and extreme CIN. Overall, 49.29% of the analyzed epithelial cells fell within these two categories. Among them, 68.8% corresponded to cells with very low CIN, primarily derived from control subjects C2 and C3, with a minor contribution from patient T5, previously identified as exhibiting low CIN87 expression. The remaining 31.2% represented cells with extreme CIN, originating from patients H4, T3, and T4.

**Figure 5:**
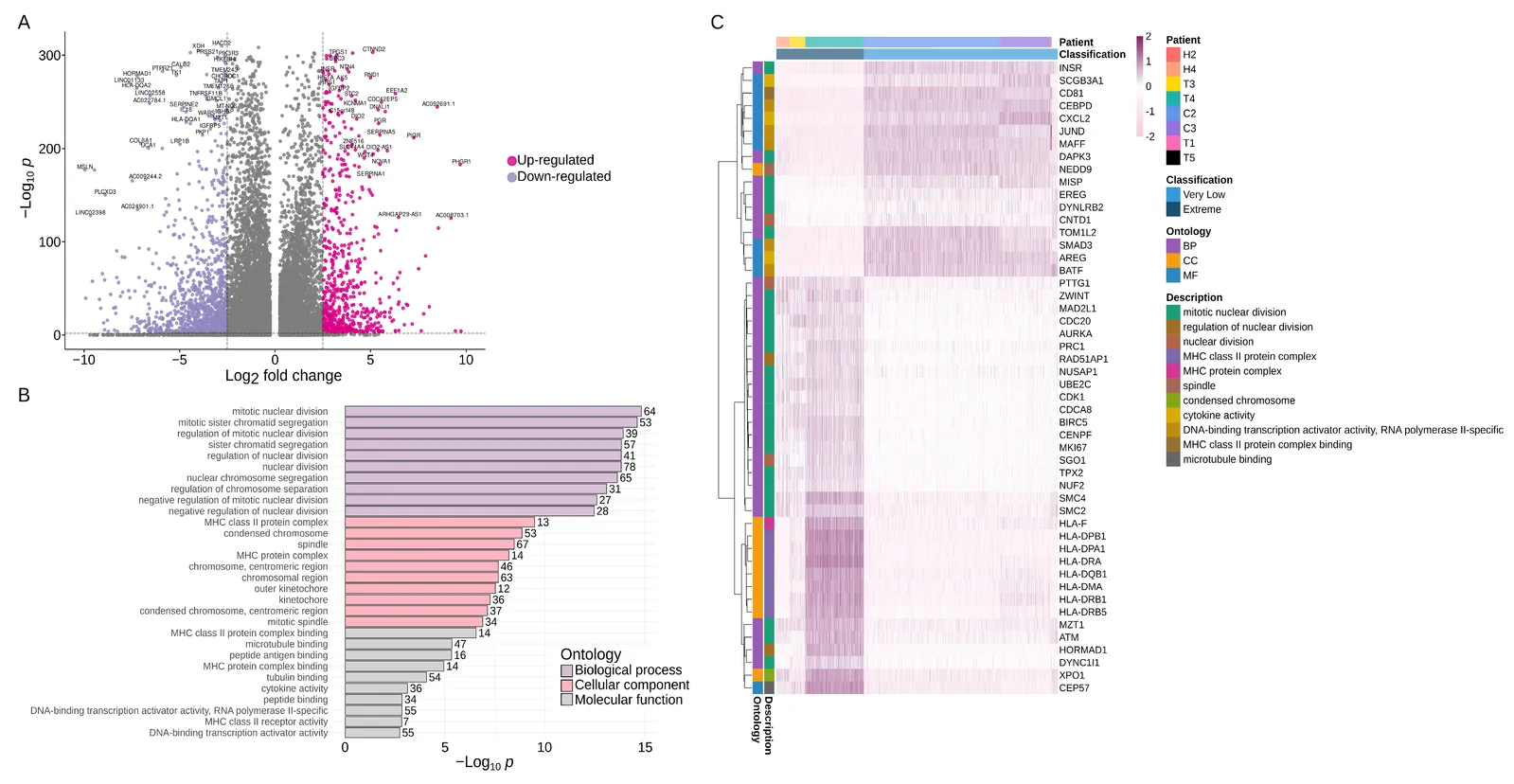
Transcriptional signature of differentially expressed genes between epithelial cells with very low and extreme CIN. (**A**) Volcano plot showing differentially expressed genes (DEGs) between epithelial cells classified by CIN87 signature expression. A total of 1958 DEGs were identified. (**B**) Gene Ontology (GO) enrichment analysis of DEGs, highlighting biological processes related to mitosis and chromosomal regulation, including sister chromatid segregation, mitotic nuclear division, and chromosome segregation. (**C**) Heatmap of the top 50 DEGs selected by adjusted *p*-value and fold change, illustrating distinct expression patterns between cells with very low and extreme CIN. The top of the heatmap indicates CIN classification and patient origin. BP, Biological process; CC, Cellular component; MF, Molecular function.

When both cell groups were compared, a total of 1958 DEGs exhibiting significant changes were identified (Supplementary Table S6). Functional enrichment analysis using the clusterProfiler package (v4.18.1), showed that these DEGs are significantly associated with biological processes including sister chromatid segregation, nuclear division, mitotic nuclear division, and chromosome segregation (Figure 5A,B). Many of these genes are involved in mitotic progression and cell cycle regulation, in agreement with their potential role in CIN development. Dysregulation of these processes may promote CIN by impairing sister chromatid cohesion or causing segregation errors, such as aberrant kinetochore–microtubule attachments (Figure 5B) [29,30,31].

Building on these findings, we next examined the expression patterns of the most relevant DEGs within the enriched biological processes. A heatmap of the top 50 representative genes, selected based on statistical significance and/or the magnitude of expression change, revealed distinct transcriptional profiles between cells with very low and extreme CIN. Notably, cells exhibiting extreme CIN showed a predominant upregulation of genes associated with key biological processes (BP) and cellular components (CC) compared to those with very low CIN (Figure 5C). Among the most upregulated DEG in the extreme CIN group, we found genes related to the histocompatibility complex class II (MHC II), as well as, key regulators of the cell cycle and mitosis, including *MKi67*, *CKS2*, *MAD2L1*, *CENPF*, *SMC4*, *UBE2C*, and *MZT1*. This transcriptional pattern suggests enhanced mitotic activity and proliferative potential in cells with extreme CIN. Conversely, genes such as *CXCL2*, *DAPK3*, *BATF*, and *AREG* were markedly downregulated in the extreme CIN group. These genes are involved in pathways that modulate the tumor microenvironment, apoptosis, cell growth signaling, and inflammation. Their reduced expression may reflect altered cellular homeostasis, potentially compromising immune response, intercellular communication, and genomic stability. Interestingly, none of the genes assessed in the CIN87 signature overlapped with the top 50 genes identified using this approach, suggesting that they could be independent biomarkers. Furthermore, the pseudobulk analysis comparing patients showed convergency among the genes and gene ontology terms providing support for our findings. Complete results from the pseudobulk sensitivity analysis, including the 516 DEGs and corresponding GO enrichment visualizations, are provided in Supplementary Figure S1 and Supplementary Table S7.

## 4. Discussion

BC remains the most frequently diagnosed malignancy and a leading cause of cancer-related mortality among women worldwide. Despite advances in precision oncology, tumor heterogeneity continues to hinder effective clinical management and accurate prediction of therapeutic outcomes. In this context, CIN has emerged as a critical hallmark of tumor evolution and a potential biomarker of BC aggressiveness, influencing genomic diversity, treatment resistance, and disease progression [2]. To deepen our understanding of CIN’s biological significance and molecular mechanisms, we analyzed scRNA-seq data from healthy breast tissue, HER2+ and TNBC samples to estimate cell type composition and explored CIN-associated gene expression signatures.

The transcriptional profiles obtained in this study provide key insights into the tumor microenvironment (TME) and its central role in BC development and progression [30]. The TME, composed of immune cells, cancer-associated fibroblasts, vascular and adipose cells, and extracellular matrix components, supports tumor growth and facilitates invasion and immune evasion [31]. Notably, we observed increased immune infiltration in HER2+ and TNBC samples (Figure 1C), consistent with previous reports describing these subtypes as highly immunogenic and “immune-hot” tumors [32,33]. We next compared different CIN-related gene expression signatures to assess their ability to characterize CIN-associated transcriptional states across BC subtypes. Although we did not find statistic significant differences among the three signatures assessed at the patient level, we found differences at the single cell level, suggesting the composite CIN87 signature may provide greater resolution for distinguishing transcriptional differences among samples and facilitate the stratification of BC subtypes within our single-cell dataset. Importantly this composed signature has not been validated and although useful in our study, more studies are required to demonstrate enhanced biological, prognosis or clinical performance. Using this approach, we observed elevated CIN levels and pronounced heterogeneity among HER2+ and TNBC samples, consistent with their reported genomic instability and poor prognosis [34,35]. These findings are consistent with the proposed role of CIN in tumor evolution through the promotion of genetic heterogeneity and adaptive potential [36].

This relationship is particularly evident in TNBC, which is widely recognized as the BC subtype with the highest levels of CIN but is also characterized by remarkable molecular and genomic heterogeneity. In agreement with previous reports, our results showed that most TNBC patients clustered within the high or extreme CIN categories. However, substantial inter-individual variability was observed, indicating that CIN patterns differ not only between molecular subtypes but also among patients within the same subtype. Patient T5 exemplified this variability by exhibiting a CIN87 profile comparable to, or even lower than, that of healthy controls. While the influence of tumor purity or normal tissue admixture cannot be completely excluded, no evidence of technical artifacts was identified during data quality assessment. Furthermore, because the CIN87 signature represents a transcriptomic surrogate rather than a direct measurement of chromosomal alterations, low scores in individual tumors may reflect biological diversity rather than analytical error. This interpretation is supported by current evidence demonstrating that TNBC comprises biologically distinct molecular entities with different transcriptional programs, evolutionary trajectories, and degrees of genomic instability. Consequently, not all TNBC tumors necessarily exhibit the same extent of CIN despite sharing the same clinical classification. Although the molecular characteristics of patient T5 were not further investigated in this study, its relatively low CIN87 score may therefore represent the intrinsic biological heterogeneity of TNBC rather than a measurement artifact. The marked interpatient variability in CIN87 expression further supports the concept that CIN is a dynamic and patient-specific process rather than a fixed tumor characteristic, likely reflecting differences in clonal architecture and the selective pressures acting during tumor evolution. In agreement with this interpretation, the increased CH observed in BC patients, particularly in TNBC, compared with healthy controls suggests that tumor evolution is driven by genetically diverse subpopulations that promote adaptation and disease progression. Together, these findings indicate that tumors sharing the same molecular subtype may nonetheless follow distinct genomic evolutionary trajectories and exhibit different degrees of CIN. This observation is consistent with previous reports describing TNBC as a molecularly heterogeneous disease with distinct genomic instability profiles and clinical behavior [3,37]. Therefore, T5 may represent a relatively chromosomally stable TNBC phenotype, highlighting the complexity of CIN biology and emphasizing the need for complementary cytogenetic, transcriptomic, and genomic analyses in larger cohorts to clarify the biological significance of these uncommon cases and to determine whether individualized CIN assessment may improve biological characterization and prognostic stratification.

The marked interpatient variability in CIN87 expression, even within the same molecular subtype, further supports the notion that CIN is a dynamic and patient specific process rather than a fixed tumor feature. This variability likely reflects differences in clonal architecture and selective pressures within individual tumors. Notably, cases with unexpectedly low CIN levels, such as T, highlight that not all tumors within a subtype share the same genomic trajectory, underscoring the value of individualized CIN assessment for refined prognostic and therapeutic stratification. Consistently, the observed increase in CH among BC patients (particularly in TNBC) compared with healthy controls suggests that tumor evolution is shaped by genetically diverse subpopulations, fostering adaptation and disease progression.

The results of this study also suggested a positive trend between CIN and CH, consistent with their interdependence as fundamental drivers of intratumoral heterogeneity and tumor evolution. Although correlations with clinical parameters did not reach statistical significance, the consistent trends, particularly the higher CIN, CH, and Ki67 levels observed in TNBC compared with HER2+ tumors, are biologically significant and consistent with the highly unstable and aggressive phenotype of this subtype [38,39]. Furthermore, the higher values across all three parameters (CIN, CH, and Ki67 levels), detected in TNBC cases with lymphovascular invasion, reinforce the notion that CIN driven clonal diversification may contribute to the distinct molecular behavior and unfavorable clinical outcomes characteristic of this subtype.

Interestingly, the CNV analysis revealed recurrent and subtype-specific chromosomal alterations that may underlie the genomic mechanisms driving CIN in BC. TNBC samples showed a higher CNV burden, with frequent gains in 1q and 8q, and losses in 5q and 17p, consistent with basal-like tumor profiles [35,37]. Deletions of 17p, encompassing *TP53*, and losses in 5q disrupt key genes in genome maintenance and metastasis regulation [40,41]. Conversely, gains in 1q and 8q promote oncogene amplification, increased proliferation, and poorer outcomes [42]. In HER2+ tumors, 17q amplification has been linked to clinical recurrence [43,44]. Overall, these recurrent CNVs reflect the elevated CIN typical of aggressive BC subtypes and support its role as a key driver of tumor evolution and therapeutic resistance [2,45].

Moreover, transcriptional profiling of epithelial cells stratified by CIN levels revealed marked molecular differences between low and extreme CIN cell populations, highlighting CIN’s strong impact on BC biology. Tumor samples showed enrichment of extreme CIN cells, while controls were dominated by low CIN profiles, reinforcing the link between CIN and malignant transformation. Functional enrichment analyses revealed overrepresentation of mitotic and chromosomal segregation pathways, core processes whose dysregulation drives genomic instability [46,47]. Cells with extreme CIN exhibited upregulation of genes regulating mitosis and chromosomal segregation (*MKi67*, *CKS2*, *MAD2L1*, *CENPF*, *SMC4*, *UBE2C* and *MZT1*), previously associated with tumor aggressiveness and poor prognosis [48,49]. The overexpression of these regulators has been linked to increased proliferative capacity, centrosome amplification and chromosome missegregation, hallmarks of aggressive tumor behavior [48,50,51]. In particular, *MKi67* and *UBE2C* are well established markers of mitotic hyperactivation and poor clinical outcomes [52], while dysregulation of CENPW, SMC2, AURKA and PRC1, has been linked to CIN due to their critical roles in controlling cell division [53]. Conversely, the downregulation of *CXCL2*, *DAPK*, and *BATF* suggests suppression of apoptotic and immune pathways, contributing to immune evasion [54,55,56,57,58].

Collectively, these results suggest that CIN is not merely a byproduct of mitotic dysfunction, but a broader adaptive program involving coordinated deregulation of cell cycle, immune, and stress-response pathways. Recognizing these interconnections highlights the potential of CIN and CH as complementary indicators of tumor complexity whose integration into molecular profiling frameworks could refine subtype characterization, improve risk prediction, and guide more precise therapeutic strategies. However, these findings should be interpreted with caution given the limited sample size and the lack of statistical significance in some comparisons. The relatively small cohort resulted from the application of stringent quality-control criteria to ensure the reliability of the scRNA-seq data. In addition, publicly available scRNA-seq datasets for HER2+ and TNBC remain scarce, particularly those with comprehensive clinicopathological annotation, genomic profiling, and longitudinal follow-up data. This limitation is further compounded by the lower prevalence of these subtypes compared with luminal breast cancers, reducing the availability of well-characterized cohorts. Although the CIN87 signature provided greater resolution for characterizing CIN-associated transcriptional states within this carefully curated single-cell dataset, these findings require validation in larger, independent cohorts. Future studies integrating genomic alterations, clinical outcomes, and therapeutic response data will be essential to determine the robustness and reproducibility of CIN87 and to establish whether the integration of complementary CIN-associated transcriptional signatures provides additional biological, prognostic, or predictive information beyond existing CIN signatures.

## 5. Conclusions

Our results highlight the potential usage of scRNA-seq to evaluate CIN in tumor clinical samples. The integration of transcriptomic and inferred copy number analyses suggests that chromosomal alterations not only contribute to clonal diversity but also influence tumor microenvironment interactions, potentially promoting tumor aggressiveness, particularly in TNBC and, to a lesser extent, HER2+ tumors. The observed CIN patterns are consistent with the concept of a functional threshold, in which intermediate levels of instability may confer adaptive advantages while excessive instability becomes detrimental to tumor cell fitness. Furthermore, the dysregulation of mitotic regulators and the increased expression of proliferation-associated genes provide additional evidence of the molecular programs underlying CIN and their potential contribution to immune evasion and tumor survival. Finally, our findings indicate that integrating complementary CIN-associated transcriptional signatures, such as CIN70 and CIN17, may improve the resolution of CIN-related transcriptional states in single-cell datasets. Further validation in larger, independent cohorts integrating genomic, clinical, and therapeutic response data will be required to determine the broader biological and clinical relevance of this approach.

## Figures and Tables

**Table 1:** General clinical and demographic characteristics of HER2+ and TNBC patients and control individuals.

Variable	HER2+ Patients (GSE176078, *n* = 4)	TNBC Patients (GSE176078, *n* = 7)	Controls (GSE180878, *n* = 3)
Age (Years)	52.5 (43–60)	52.4 (35–67)	38.7 (36–41)
Tumor Grade	Grade 3 (100%)	Grade 3 (100%)	-
ER Expression (%)	0 (100%)	0 (100%)	NA
PR Expression (%)	0 (100%)	0 (100%)	NA
HER2 (IHC Score)	3+ (100%)	0 (100%)	NA
Ki67 (%)	55% (30–80%)	63% (40–80%)	NA
Lymphovascular invasion	2 (50%)	4 (57%)	NA
Surgery Performed	Tumor resection (100%)	Tumor resection (100%)	Breast reduction mammoplasty (100%)
Cancer History	No reported	No reported	Negative (100%)
BRCA Genotype	No reported	No reported	WT (100%)

HER2+, human epidermal growth factor receptor 2 positive; TNBC, triple-negative breast cancer; ER, estrogen receptor; PR, progesterone receptor; IHC, immunohistochemistry; WT, wild-type.

## Data Availability

The original data presented in the study are openly available in GEO datasets at https://www.ncbi.nlm.nih.gov/geo/query/acc.cgi?acc=GSE176078 (accessed on 1 December 2025) and https://www.ncbi.nlm.nih.gov/geo/query/acc.cgi?acc=GSE180878 (accessed on 1 December 2025).

## Supplementary Materials

The following supporting information can be downloaded at https://www.mdpi.com/article/10.3390/biomedicines14091902/s1, Table S1. Datasets analyzed in this study. Table S2. Number of cells before and after quality control filtering. Table S3. True diversity analysis and Shannon index. Table S4. Correlation between clinicopathological features, CIN and CH. Table S5. Comparison between clinicopathological features, CIN and CH among tumor subtypes. Table S6. List of DEG between epithelial cells with very low and extreme CIN. Table S7. List of DEG between epithelial cells with very low and extreme CIN by patient. Figure S1. Transcriptional signature of differentially expressed genes between patients with very low and extreme CIN.
