# Supplementary material for: Single-Cell RNA-Seq Reveals Chromosomal Instability-Associated Transcriptomic Profiles in Breast Cancer
Source: Biomedicines. 2026 Aug 26;14(9):1902. doi: 10.3390/biomedicines14091902 (PMC13604525; doi:10.3390/biomedicines14091902)
Supplement: Supplementary file 1 [file biomedicines-14-01902-s001.zip › biomedicines-4425341-supplementary.pdf]

Supplementary figures

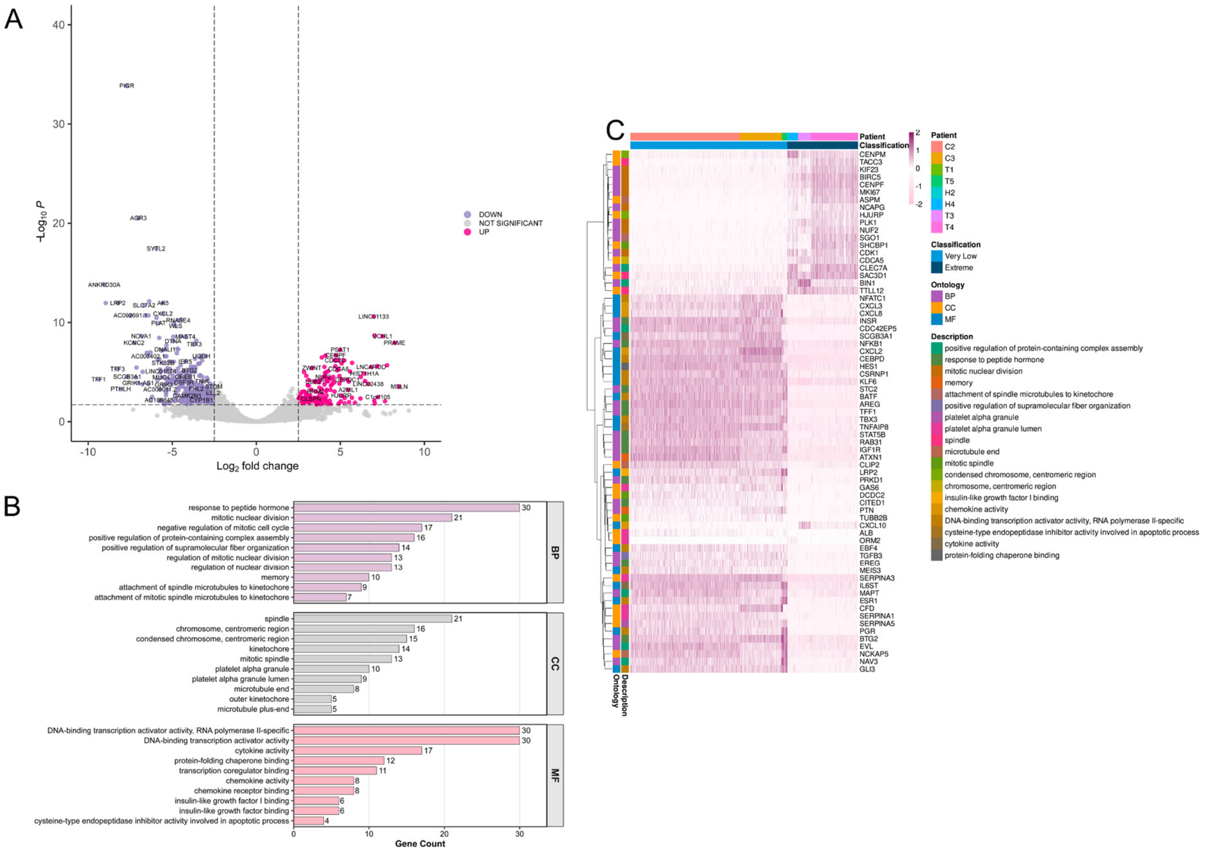

Figure S1. Transcriptional signature of differentially expressed genes between patients with very low and extreme CIN. Pseudobulk differential expression analysis was conducted at the sample/patient level to compare epithelial cell aggregates classified with low versus extreme CIN87 scores, following the exclusion of CIN87 signature genes. **(A)** Volcano plot showing differentially expressed genes (DEGs) identified from the pseudobulk analysis. A total of 516 DEGs were identified (p_adj < 0.05). **(B)** Gene Ontology (GO) enrichment analysis of the 516 DEGs, highlighting highly conserved biological processes related to chromosome regulation and mitosis, including sister chromatid segregation, mitotic nuclear division, and chromosome segregation. **(C)** Heatmap of the top 50 DEGs (selected by adjusted p-value and absolute fold change), displaying distinct expression profiles across pseudobulk samples from low and extreme CIN groups. Sample annotations at the top indicate CIN classification and patient origin.

Supplementary tables

Supplementary Table S1. Datasets analysed in this study.

Original Sample ID	Assigned Code	GSE	Reference
HER3586	H1	GSE176078	Wu SZ, Al-Eryani G, Roden DL, Junankar
HER3838	H2		

HER3921	H3		S et al. A single-cell and spatially resolved atlas of human breast cancers. Nat Genet 2021 Sep;53(9):1334-1347. PMID: 34493872
HER45171	H4		
TNBC3946	T1		
TNBC4465	T2		
TNBC4495	T3		
TNBC4515	T4		
TNBC44041	T5		
TNBC44971	T6		
TNBC44991	T7		
C360	C1	GSE180878	Gray GK, Li CM, Rosenbluth JM, Selfors LM et al. A human breast atlas integrating single-cell proteomics and transcriptomics. Dev Cell 2022 Jun 6;57(11):1400-1420.e7. PMID: 35617956
C361	C2		
C362	C3		

Supplementary Table S2. Number of cells before and after quality control filtering

Sample	Cells (Unfiltered)	Cells (Filtered)
H1	4580	4013
H2	4035	3656
H3	4225	3650
H4	3136	2973
T1	1350	876
T2	1801	1064
T3	16199	13149
T4	6096	5158
T5	2392	2359
T6	12230	9883
T7	9023	7231
C1	5419	5194
C2	8520	8226
C3	5075	4849
Total	84081	72281

Mean ± SD	6006 ± 4197	5171 ± 3434

Supplementary Table S3. True diversity analysis and Shannon index

Patient	Group	SDI_CIN87	DV_CIN87
C1	Control	0.8208078651	2.272334836
C2	Control	0.5855760391	1.796025269
C3	Control	0.7844320525	2.191162122
H1	HER2+	0.9101727283	2.484751683
H2	HER2+	1.277034259	3.585988827
H3	HER2+	1.261118607	3.529367254
H4	HER2+	1.534934298	4.641020594
T1	TNBC	0.8230393171	2.277411104
T2	TNBC	1.577679154	4.84370127
T3	TNBC	1.755467178	5.786150275
T4	TNBC	1.653341649	5.224408833
T5	TNBC	0.4349201515	1.544839701
T6	TNBC	1.540948619	4.669017289
T7	TNBC	1.527475534	4.606533095

Supplementary Table S4. Correlation between clinicopathological features, CIN and CH

	**CIN_87**	**True diversity**	**Age**	**Ki67**	**Lymphovascular invasion**	**Subtype**
**CIN_87**	1.00	0.95	0.42	0.18	NA	NA
**True diversity**	0.95	1.00	0.47	0.16	NA	NA
**Age**	0.42	0.47	1.00	0.22	NA	NA
**Ki67**	0.18	0.16	0.22	1.00	NA	NA
**Lymphovascular invasion**	NA	NA	NA	NA	NA	0.16
**Subtype**	NA	NA	NA	NA	0.16	NA

Supplementary Table S5. Comparison between clinicopathological features, CIN and CH among tumor subtypes

**Variable**	**HER2_breast_cancer_Mean**	**TNBC_Mean**	**P_Value**
CIN_87	0.21	0.43	0.55
True diversity	0.49	0.55	0.91
Ki67	0.44	0.63	0.07

Age	53.67	52.50	1.00

Supplementary Table S6. List of DEG between epithelial cells with very low and extreme CIN.

Gene	p_val	avg_log2FC	pct.1	pct.2	p_val_adj
DTNA	0	5.446671331	0.777	0.039	0
LINC00486	0	-4.025921316	0.048	0.753	0
CXCL2	0	5.645356661	0.723	0.037	0
CALML5	0	-5.047088788	0.187	0.836	0
XIST	0	2.95760032	0.745	0.111	0
ATXN1	0	4.258161953	0.725	0.109	0
MTHFD2L	0	4.530062005	0.724	0.115	0
AC010343.3	0	-8.730837263	0.005	0.599	0
IER5	0	4.81868099	0.708	0.114	0
RBPMS	0	3.248106251	0.734	0.151	0
SAMD4A	0	4.289325106	0.703	0.122	0
TFF3	0	9.724302798	0.591	0.01	0
TTTY14	0	-4.770650689	0.057	0.635	0
PSMB9	0	-5.269522165	0.083	0.658	0
AC024230.1	0	-6.645186272	0.019	0.593	0
FABP5	0	-3.782515308	0.109	0.682	0
S100A4	0	-6.613812296	0.097	0.664	0
FGFR1	0	5.041466441	0.614	0.049	0
PYCR1	0	-3.235871807	0.197	0.761	0
FNIP1	0	3.511174609	0.681	0.121	0
COTL1	0	-3.920620703	0.158	0.705	0
HLA-DPA1	0	-5.072280415	0.074	0.616	0
TMEM238	0	5.636428121	0.58	0.04	0
RARRES1	0	-5.084028994	0.161	0.695	0
SDHD	0	-3.394404021	0.238	0.77	0
HLA-DRA	0	-4.7870121	0.162	0.694	0
ANKRD30A	0	5.520287162	0.541	0.01	0
FAM136A	0	-2.854892574	0.217	0.741	0
SCGB3A1	0	9.430902713	0.533	0.009	0
CEP57	0	-3.290034807	0.209	0.732	0
CDK6	0	-3.221803759	0.147	0.668	0
JUND	0	3.706259556	0.878	0.365	0

DUXAP8	0	-2.838662273	0.156	0.668	0
HLA-DPB1	0	-4.882177477	0.119	0.63	0
FSCN1	0	-3.259413143	0.156	0.662	0
LEMD1	0	-7.113880777	0.007	0.51	0
XPO1	0	-2.849889992	0.154	0.656	0
CKS1B	0	-2.808969496	0.274	0.772	0
AREG	0	5.40904278	0.827	0.33	0
TFF1	0	7.657264395	0.501	0.006	0
EFNA5	0	-2.918855297	0.14	0.633	0
CEBPD	0	4.49246272	0.922	0.436	0
BACH2	0	3.432270807	0.59	0.104	0
MRAS	0	-3.96387347	0.079	0.565	0
PSMG3	0	-3.067368324	0.223	0.706	0
ERBB4	0	3.074564864	0.589	0.108	0
LINC00393	0	-7.208419628	0.01	0.489	0
HLA-DRB1	0	-3.439200044	0.143	0.613	0
AFF3	0	4.475118384	0.538	0.071	0
C6orf15	0	-8.408887681	0.019	0.486	0
CACNA1C	0	-3.214814449	0.044	0.51	0
CKS2	0	-2.99783356	0.244	0.709	0
IFI27	0	-5.475140596	0.088	0.552	0
HLA-DRB5	0	-4.430782443	0.058	0.518	0
DNAJC12	0	5.456088512	0.479	0.022	0
PLXDC2	0	-3.071353592	0.121	0.578	0
CD81	0	3.052549904	0.827	0.374	0
TIMP3	0	-2.988861696	0.145	0.595	0
DAPK3	0	2.576516919	0.742	0.293	0
THSD4	0	3.335529485	0.732	0.283	0
AHNAK2	0	-5.6579922	0.022	0.471	0
IL6R	0	3.913346097	0.519	0.072	0
AGR3	0	5.408670899	0.465	0.02	0
TANC2	0	3.70619244	0.504	0.061	0
HLA-DQB1	0	-3.603125651	0.111	0.553	0
CAV1	0	-2.945280578	0.163	0.604	0
WDPCP	0	-2.518868193	0.182	0.621	0
HLA-DMA	0	-3.637960316	0.099	0.536	0
PDLIM4	0	3.774864763	0.493	0.059	0
PPP3R1	0	-2.580868638	0.21	0.643	0
TBC1D5	0	-2.659174803	0.23	0.659	0

SYTL2	0	5.16790199	0.508	0.079	0
C3orf52	0	4.181229468	0.489	0.063	0
KLK6	0	-3.201822661	0.078	0.503	0
PLAT	0	4.54860963	0.501	0.077	0
HLA-F	0	-3.297007278	0.102	0.525	0
GLYATL2	0	-4.638023049	0.038	0.451	0
MAST4	0	3.331051345	0.597	0.188	0
MAGED2	0	3.033736149	0.645	0.238	0
TSPAN5	0	3.001238601	0.53	0.124	0
UGP2	0	-2.57983652	0.408	0.814	0
MYOF	0	2.706661423	0.804	0.399	0
PTHLH	0	6.482825979	0.436	0.031	0
SLC7A2	0	4.661674628	0.414	0.01	0
TMEM123	0	-3.450491673	0.516	0.918	0
WEE1	0	2.956011535	0.623	0.221	0
MAFF	0	2.613282998	0.809	0.41	0
IGF1R	0	3.112412405	0.68	0.289	0
DUSP5	0	4.099759905	0.532	0.145	0
KLHL5	0	3.972862843	0.452	0.065	0
HES4	0	2.538387281	0.799	0.413	0
BCAM	0	3.377785601	0.599	0.215	0
NEDD9	0	2.690796651	0.629	0.245	0
H1FX	0	3.452391324	0.716	0.332	0
IGFBP4	0	3.74489183	0.456	0.074	0
LARGE1	0	2.562125899	0.516	0.136	0
VIM	0	-2.996411621	0.417	0.795	0
UGCG	0	2.97146851	0.57	0.193	0
NEDD4L	0	2.52015579	0.875	0.506	0
MTRNR2L8	0	4.312684238	0.427	0.059	0
FHL2	0	3.001933839	0.722	0.358	0
ARPC1B	0	-3.238813124	0.413	0.775	0
TNFAIP8	0	3.256467589	0.599	0.237	0
TNIP1	0	2.589784602	0.734	0.374	0
SRGAP1	0	2.653673511	0.684	0.327	0
TPD52L1	0	-2.605087427	0.432	0.786	0
ADIRF	0	3.215213978	0.686	0.335	0
RAB11FIP1	0	3.006363961	0.721	0.37	0
TMEM45B	0	6.305066263	0.358	0.015	0
SHB	0	3.159205696	0.573	0.234	0

GADD45B	0	3.26397914	0.696	0.359	0
TIAM1	0	3.811561104	0.397	0.073	0
ANO6	0	2.553499648	0.674	0.351	0
SMAD3	0	2.9967888	0.61	0.288	0
SFMBT2	0	3.779913592	0.395	0.075	0
FBXL2	0	2.759333721	0.487	0.167	0
TCIM	0	3.553585372	0.58	0.271	0
BATF	0	3.65729961	0.511	0.208	0
TRIO	0	2.976611866	0.897	0.596	0
MAML2	0	-2.664694384	0.369	0.669	0
DLGAP1	0	-2.889526463	0.295	0.595	0
CCND1	0	-2.841864987	0.405	0.704	0
CCDC74A	0	4.212323529	0.312	0.034	0
TFPI	0	4.507358159	0.302	0.031	0
AZGP1	0	2.532276829	0.926	0.663	0
TOX3	0	4.850320464	0.277	0.022	0
MYO1B	0	2.521533396	0.414	0.16	0
SEMA3E	0	4.20999476	0.311	0.057	0
SERPINA3	0	2.783113926	0.884	0.487	0
INPP4B	0	3.566081898	0.501	0.197	0
GNG4	0	-5.413616162	0.019	0.453	0
CTSC	0	-4.563121268	0.124	0.584	0
GNA15	0	3.53781676	0.435	0.084	0
DNAH5	0	4.927789942	0.331	0.045	0
HACD2	0	-2.795386906	0.163	0.602	0
XDH	0	-4.015170365	0.088	0.504	0
CTNND2	0	5.110940228	0.306	0.019	0
BCL11A	0	-4.435001846	0.038	0.543	0
AC025580.2	0	4.063822895	0.438	0.092	0
PRSS21	0	-3.536401916	0.11	0.501	0
TPGS1	0	3.233291033	0.352	0.067	0
TOM1L2	0	2.692831059	0.599	0.267	0
CLU	0	2.878931014	0.738	0.204	0
PIK3R3	0	-2.614036363	0.199	0.645	0
MTIF2	0	-3.012235189	0.112	0.615	0
TNIK	0	3.181610017	0.374	0.07	0
ADGRV1	0	2.704440804	0.338	0.104	0
BBC3	0	3.176258888	0.571	0.198	0
HIKESHI	0	-2.618080812	0.214	0.657	0

SLC5A1	0	-2.584733997	0.102	0.46	0
CALB2	0	-4.924597684	0.032	0.451	0
NTN4	0	3.779270966	0.491	0.136	0
MREG	0	3.127290069	0.477	0.149	0
INSR	0	2.764659938	0.678	0.224	0
TUBA1A	0	2.508026443	0.734	0.335	0
PTPRZ1	0	-5.886677747	0.011	0.445	0
TMC5	0	3.861616313	0.422	0.101	0
TMEM243	0	-2.64758245	0.156	0.578	0
MACC1	0	2.526356904	0.618	0.239	0
DUSP10	0	2.826241738	0.389	0.131	0
SESN3	0	-3.576727159	0.071	0.51	0
TK1	0	-5.166062113	0.035	0.556	0
RASGEF1B	0	-2.527278242	0.164	0.56	0
HORMAD1	0	-7.213204649	0.003	0.425	0
RND1	0	5.000349428	0.36	0.041	0
CHORDC1	0	-2.603602858	0.173	0.627	0
HIF1A	0	2.659315124	0.491	0.176	0
AK5	0	3.511351315	0.387	0.045	0
LINC01133	0	-7.632202165	0.004	0.416	0
TAP1	0	-2.820561795	0.208	0.609	0
IRS1	0	2.827622114	0.326	0.083	0
RNASE4	0	3.533481334	0.407	0.066	0
TMEM126B	0	-2.959433873	0.174	0.637	0
HLA-DQA2	0	-7.245237061	0.005	0.436	0
ROCR	0	-2.594862914	0.108	0.468	0
IGFBP2	0	3.361696502	0.383	0.167	0
GLI3	0	2.855360159	0.27	0.03	0
DENND5A	0	2.680870545	0.531	0.189	0
HDGFL3	0	2.717734492	0.261	0.04	0
NIBAN1	0	2.644207748	0.432	0.155	0
EEF1A2	0	6.305843664	0.205	0.005	0
LINC02558	0	-6.542967147	0.006	0.341	0
STC2	0	4.005165003	0.442	0.157	0
MICAL2	0	3.320228937	0.389	0.146	0
TNFRSF11B	0	-3.624850071	0.121	0.462	0
AGR2	0	3.449622288	0.492	0.179	0
SMC4	0	-3.570561117	0.108	0.609	0
KCNMA1	0	4.205131419	0.39	0.104	0

GMCL1	0	-3.066275722	0.092	0.601	0
IER3	0	2.63324311	0.858	0.603	0
NCKAP5	0	2.763449964	0.329	0.079	0
CDC42EP5	0	5.567790413	0.368	0.013	0
BARX2	0	2.707877886	0.477	0.174	0
AC022784.1	0	-6.566869771	0.008	0.459	0
EVA1B	0	2.901031139	0.419	0.157	0
COL4A5	0	2.98088843	0.385	0.154	0
CDKN1A	0	2.640820438	0.477	0.133	0
AC092691.1	0	8.490348335	0.19	0.002	0
SERPINE2	0	-4.769289984	0.055	0.543	0
AR	0	2.514626937	0.318	0.089	0
MT-ND6	0	-2.528912259	0.224	0.619	0
DNALI1	0	5.400861228	0.212	0.004	0
CTXN1	0	2.870696537	0.255	0.074	0
MAFB	0	-2.93747496	0.096	0.516	0
UPK3A	0	5.769343654	0.175	0.005	0
ANGPTL1	0	2.920269049	0.304	0.099	0
IL18	0	-4.654030291	0.044	0.429	0
C15orf48	0	3.506113684	0.476	0.119	0
IL4R	0	3.288689186	0.366	0.097	0
IGHGP	0	-2.642447287	0.008	0.377	0
PHYHD1	0	3.336498383	0.322	0.048	0
WARS	0	-3.471992759	0.137	0.585	0
BTRC	0	2.581133366	0.361	0.14	0
SGCD	0	-3.31913655	0.11	0.451	0
CXCL17	0	-2.930556267	0.151	0.582	0
DIO2	0	4.270787707	0.253	0.046	0
SDHAF3	0	-3.139734898	0.099	0.59	0
ZSCAN18	0	2.626847072	0.317	0.085	0
MZT1	0	-2.87520786	0.125	0.586	0
ENPP1	0	3.606462053	0.204	0.035	0
HLA-DQA1	0	-4.653914088	0.023	0.442	0
TBX3	0	2.877006779	0.441	0.166	0
PHLDB2	0	2.835689489	0.38	0.129	0
AL136454.1	0	-2.666171201	0.093	0.513	0
PGR	0	5.419979725	0.211	0.009	0
AC011287.1	0	-4.433771472	0.046	0.452	0
NUDT1	0	-3.154420069	0.144	0.631	0

LINC00504	0	3.028389572	0.276	0.087	0
WLS	0	3.674739439	0.291	0.043	0
IGFBP5	0	-3.387030622	0.087	0.512	0
SYTL4	0	4.000467746	0.228	0.051	0
RAB31	0	2.65541233	0.378	0.155	0
CLCF1	0	3.382962251	0.415	0.121	0
NPAS3	0	-2.833741855	0.099	0.457	0
NFIX	0	-2.552678447	0.215	0.668	0
SERPINA5	0	5.486883764	0.193	0.01	0
PKP1	0	-3.799905442	0.036	0.446	0
C16orf89	0	4.393982553	0.24	0.021	0
ABR	0	3.160237518	0.27	0.065	0
C6orf141	0	2.668581973	0.207	0.088	0
CPE	0	2.615374345	0.302	0.139	0
STAT5B	0	2.619605495	0.423	0.19	0
PIGR	0	7.261816098	0.397	0.009	0
FBP1	0	2.732996684	0.278	0.075	0
B4GALNT3	0	-3.132567955	0.101	0.363	0
SH3GLB2	0	2.932545519	0.344	0.119	0
COL8A1	0	-7.023911195	0.005	0.392	0
LRP1B	0	-4.979588428	0.045	0.398	0
ZNF516	0	4.040259739	0.221	0.019	0
SEMA3B	0	4.012410404	0.232	0.027	0
CCDC82	0	-3.002958347	0.082	0.527	0
FCHSD2	0	-2.795471546	0.144	0.543	0
RNF223	0	3.793807412	0.291	0.04	0
TM7SF3	0	-2.787747698	0.151	0.65	0
UCA1	0	-6.627459641	0.007	0.399	0
KCNMB2-AS1	0	-5.903545457	0.004	0.368	0
SLC44A4	0	4.206277977	0.205	0.017	0
DIO2-AS1	0	5.384501349	0.185	0.02	0
LURAP1L-AS1	0	2.528074677	0.297	0.116	0
CACNG4	0	5.879037343	0.158	0.007	0
SCUBE2	0	3.663504003	0.244	0.03	0
FANCL	0	-3.337026833	0.066	0.498	0
CHRM3	0	-4.059887235	0.068	0.413	0
MTCL1	0	4.707581704	0.161	0.014	0
CMTM3	0	2.633681823	0.331	0.119	0
VSIG2	0	5.141835967	0.214	0.007	0

COL4A6	0	3.818221057	0.211	0.035	0
PLCB4	0	-2.764439277	0.136	0.559	0
ESR1	0	3.312061092	0.279	0.039	0
KMO	0	2.859261389	0.196	0.068	0
WNT4	0	4.665885306	0.167	0.012	0
PTPRM	0	3.268506901	0.235	0.034	0
PFDN2	0	-2.51983146	0.659	0.882	0
DUSP8	0	3.468872609	0.215	0.039	0
TNFRSF18	0	3.583147576	0.23	0.041	0
ANP32E	0	-2.924190505	0.111	0.544	0
CCDC88A	0	-4.01103952	0.044	0.465	0
DYNC1I1	0	-4.477555914	0.019	0.245	0
NOVA1	0	5.489104565	0.169	0.008	0
RASD1	0	3.677581533	0.25	0.035	0
NAV3	0	3.628860067	0.243	0.034	0
PHGR1	0	9.681175592	0.143	0	0
MISP	0	3.50333583	0.212	0.042	0
GPRC5C	0	4.165192758	0.188	0.019	0
LINC01315	0	-3.958067832	0.06	0.606	0
AXL	0	4.399148322	0.187	0.018	0
STMN1	0	-2.609569217	0.33	0.706	0
CSRNP1	0	2.9118837	0.436	0.156	0
UGDH	0	2.614564137	0.574	0.367	0
IGHG3	0	-3.835169839	0.006	0.285	0
POLR3K	0	-3.586939089	0.068	0.601	0
CYP1B1	0	3.786714788	0.217	0.032	0
AARD	0	-3.615955541	0.079	0.554	0
DNMBP	0	3.68192358	0.21	0.025	0
MSLN	0	-9.953086032	0.001	0.379	0
IGHG4	0	-9.452061132	0	0.316	0
NLRC5	0	-3.114510515	0.075	0.461	0
MRM2	0	-2.68676375	0.117	0.577	0
B3GALT6	0	3.510013055	0.255	0.048	0
IL32	0	2.827287804	0.484	0.185	0
MAN2A1	0	2.504565858	0.355	0.178	0
PIP	0	3.397268757	0.365	0.101	0
C1S	0	-3.080105351	0.101	0.509	0
FBXO2	0	-2.710289102	0.14	0.49	0
CACNA2D1	0	3.382747876	0.185	0.029	0

RHCG	0	-5.12219109	0.018	0.233	0
TMEM51	0	2.501606196	0.353	0.149	0
MCRIP2	0	-2.519798911	0.21	0.707	0
LINC02246	0	3.034556201	0.164	0.037	0
PLEKHO1	0	3.412888841	0.215	0.035	0
PRKD1	0	3.739280749	0.178	0.015	0
RALGDS	0	2.588285733	0.394	0.16	0
ABLIM3	0	3.882269848	0.191	0.041	0
SERPINA1	0	4.945933242	0.241	0.039	0
S100A2	0	-2.742259257	0.13	0.453	0
HCP5	0	-2.733031296	0.083	0.443	0
AC009244.2	0	-6.78395661	0.002	0.291	0
GGH	0	-2.692197538	0.131	0.525	0
SERPINH1	0	-3.198782988	0.108	0.568	0
EED	0	-2.972159949	0.077	0.481	0
MLIP-AS1	0	-7.479515261	0.002	0.349	0
TGM2	0	3.018990551	0.251	0.074	0
PRKAR1B	0	-2.642456452	0.15	0.546	0
CEACAM6	0	-3.971892198	0.034	0.214	0
TSPYL5	0	-3.307613571	0.038	0.387	0
LY75	0	-2.882273671	0.063	0.417	0
AASDHPPT	0	-2.734216293	0.094	0.539	0
ATM	0	-2.840478471	0.08	0.486	0
LY6D	0	-4.506258175	0.044	0.39	0
ST3GAL4	0	-2.734741409	0.161	0.619	0
PCYT2	0	-3.279338869	0.065	0.535	0
KRT23	0	4.551788629	0.49	0.107	0
AP001636.3	0	-3.788044899	0.031	0.35	0
PREX1	0	3.365624801	0.199	0.029	0
SMIM10L1	0	-3.174872635	0.106	0.628	0
UBE2T	0	-5.103310901	0.018	0.452	0
APOD	0	4.58597467	0.263	0.022	0
HPX	0	4.930180256	0.143	0.015	0
FHDC1	0	3.159009284	0.21	0.067	0
CITED4	0	3.204574425	0.516	0.234	0
TTC9	0	3.362106044	0.276	0.063	0
NBN	0	-2.628842445	0.097	0.525	0
CACYBP	0	-2.746850134	0.201	0.726	0
RGS6	0	4.047674123	0.251	0.03	0

EBF4	0	5.001435411	0.134	0.006	0
DLAT	0	-2.75071918	0.086	0.496	0
ZNF521	0	-2.945833331	0.056	0.415	0
RASSF6	0	3.225612719	0.201	0.05	0
AC093817.2	0	-2.553415907	0.093	0.444	0
PLCXD3	0	-8.888349872	0.003	0.331	0
STX11	0	3.809980358	0.272	0.036	0
TENM3	0	5.094611683	0.148	0.016	0
FZD1	0	-2.992426178	0.046	0.367	0
BMERB1	0	3.398312336	0.236	0.092	0
CXCL1	0	3.980639895	0.436	0.1	0
SRPX2	0	3.200657813	0.182	0.048	0
HS6ST2	0	4.433921716	0.158	0.008	0
CUEDC1	0	2.686267327	0.224	0.055	0
IGFL2-AS1	0	-5.728627752	0.013	0.18	0
ARNT2	0	2.702193868	0.12	0.024	0
ST8SIA1	0	-3.545855598	0.047	0.414	0
SH3KBP1	0	-2.753939843	0.074	0.435	0
AC127526.2	0	3.33872631	0.152	0.029	0
CDH19	0	-5.028893052	0.007	0.28	0
AC007319.1	0	4.087738243	0.16	0.015	0
ARV1	0	-2.771686743	0.076	0.499	0
CTU1	0	3.696660194	0.219	0.038	0
DOT1L	0	2.558689482	0.285	0.127	0
SGPP1	0	2.585107386	0.14	0.042	0
CIITA	0	-5.562822675	0.012	0.33	0
GRK6	0	2.681336732	0.264	0.09	0
PSIP1	0	-3.286971623	0.087	0.58	0
AP000787.1	0	-2.714224784	0.051	0.358	0
KDELR3	0	-2.901646904	0.08	0.485	0
PAQR4	0	-4.135325005	0.03	0.468	0
LAMB2	0	2.561708043	0.275	0.119	0
EGOT	0	3.232795063	0.283	0.111	0
CAMK2N1	0	3.199536789	0.231	0.043	0
ZNF165	0	2.948283507	0.339	0.136	0
AC024901.1	0	-7.199533287	0.005	0.388	0
HLA-DMB	0	-4.98425985	0.012	0.329	0
JRKL	0	-3.51270712	0.036	0.415	0
FNDC4	0	3.109105557	0.226	0.067	0

FRMD3	0	-3.826721688	0.05	0.421	0
FNIP2	0	2.953327732	0.265	0.112	0
SLC7A8	0	3.113041696	0.158	0.03	0
AC002066.1	0	-5.688268241	0.006	0.324	0
AC027288.3	0	3.235767196	0.233	0.077	0
CHST15	0	3.225299538	0.179	0.039	0
MEIS3	0	4.052553071	0.11	0.009	0
BIRC5	0	-5.800573275	0.014	0.328	0
LINC02398	0	-9.659463157	0.001	0.362	0
C16orf74	0	-3.275321229	0.085	0.452	0
MESP1	0	-3.439549719	0.043	0.417	0
ARHGAP29-AS1	0	6.468415159	0.129	0.008	0
MSH2	0	-2.768140762	0.057	0.409	0
MYBPC1	0	3.356436226	0.211	0.068	0
PCDH9	0	-4.711790758	0.02	0.304	0
ASF1A	0	-2.7284321	0.065	0.453	0
AC008703.1	0	9.203376581	0.111	0	0
AC020611.2	0	2.594127339	0.196	0.07	0
AKR1C3	0	-3.226977481	0.075	0.343	0
SSPN	0	-2.777979965	0.087	0.468	0
CDH13	0	-4.387291865	0.028	0.329	0
SUSD3	0	3.615099007	0.139	0.029	0
LPCAT2	0	-3.411176423	0.043	0.399	0
PSAT1	0	-5.222553002	0.015	0.426	0
SERTAD4	0	-3.510756724	0.051	0.434	0
RPP25	0	-3.076715374	0.043	0.387	0
PARM1	0	2.815049583	0.147	0.012	0
TMEM38A	0	-3.313845211	0.033	0.366	0
LINC01198	0	3.454282211	0.285	0.061	0
SOX6	0	-3.525566314	0.047	0.411	0
PCNA	0	-2.635626878	0.106	0.491	0
USB1	0	-2.58368879	0.102	0.5	0
PROS1	0	-4.305479577	0.015	0.324	0
SLITRK6	0	-5.287104946	0.004	0.213	0
PLCD3	0	3.54428653	0.141	0.029	0
CBX6	0	2.68284038	0.24	0.071	0
CLIP2	0	2.704758964	0.237	0.077	0
LINC01876	0	5.22430428	0.11	0.005	0

IMPA2	0	-2.633568311	0.126	0.541	0
ST8SIA6-AS1	0	-5.585615391	0.012	0.512	0
DBF4	0	-2.730385761	0.079	0.482	0
RIMS2	0	-3.582598562	0.025	0.189	0
TMPRSS11E	0	5.330265571	0.089	0.005	0
NRXN1	0	2.984039263	0.194	0.074	0
CCDC170	0	4.248281136	0.125	0.014	0
ROR1	0	-4.201765708	0.029	0.404	0
SERPINA11	0	8.551016507	0.101	0	0
DCLK1	0	2.751380955	0.163	0.055	0
CALML3	0	3.038369631	0.168	0.034	0
OTULINL	0	3.030397883	0.132	0.019	0
ALDH1A3	0	3.953023072	0.411	0.125	0
CLEC7A	0	-4.132593313	0.016	0.331	0
KCNC2	0	6.344634636	0.113	0.003	0
STON2	0	3.314799438	0.121	0.018	0
TEX30	0	-2.510519626	0.121	0.521	0
CENPF	0	-6.019110407	0.018	0.338	0
RHOU	0	2.572871095	0.118	0.041	0
TCEANC	0	-3.435593335	0.017	0.252	0
FSTL3	0	3.40158763	0.165	0.033	0
DEFB1	0	3.928839999	0.263	0.029	0
RGL3	0	3.767646269	0.108	0.013	0
B3GNT3	0	-2.715625083	0.05	0.333	0
LRP2	0	5.412672768	0.198	0.005	0
ISLR	0	-3.819856895	0.018	0.335	0
UBE2C	0	-5.24788992	0.018	0.341	0
SHC4	0	-4.78888115	0.009	0.174	0
LINC01956	0	-5.323606056	0.006	0.299	0
EFEMP1	0	-3.401052397	0.061	0.348	0
AC092969.1	0	-5.012902497	0.006	0.109	0
KIRREL3	0	-3.29497729	0.011	0.222	0
CXCL3	0	5.192526836	0.37	0.082	0
PODNL1	0	-3.753919289	0.02	0.303	0
VAX2	0	-4.268306464	0.016	0.321	0
PRSS41	0	-4.996056925	0.002	0.152	0
MCEE	0	-2.625045016	0.06	0.434	0
INTS13	0	-3.026505567	0.063	0.462	0
TCF4	0	2.614028216	0.131	0.034	0

SLC25A10	0	-2.744316153	0.068	0.443	0
C20orf27	0	-3.245919697	0.085	0.529	0
SHC2	0	4.629850248	0.109	0.006	0
AC136604.2	0	2.595993443	0.136	0.046	0
AC011483.1	0	-3.325963442	0.018	0.222	0
POLD3	0	-2.905873703	0.075	0.454	0
LIFR	0	2.811186506	0.122	0.021	0
MIR22HG	0	2.883073233	0.292	0.095	0
ZNF385C	0	4.288895095	0.206	0.026	0
SLC52A3	0	-3.118590281	0.036	0.32	0
ME1	0	-2.985902447	0.054	0.456	0
MAGOHB	0	-2.592860773	0.134	0.577	0
CFD	0	4.006786557	0.27	0.046	0
LRRCC1	0	-3.233092339	0.045	0.384	0
BHLHE41	0	-2.793595108	0.041	0.364	0
GON7	0	-2.605442914	0.066	0.453	0
AC015712.2	0	3.877941927	0.292	0.074	0
FERMT1	0	-4.40197939	0.014	0.341	0
AP002360.1	0	-2.694823328	0.056	0.391	0
MARCH9	0	3.105888518	0.139	0.037	0
GPNMB	0	-2.874624327	0.04	0.293	0
PCED1B-AS1	0	3.639719401	0.093	0.015	0
INPP5J	0	3.492016577	0.113	0.019	0
LBR	0	-2.58017967	0.065	0.38	0
DIPK2A	0	-2.663255061	0.049	0.373	0
IRX2	0	2.549623091	0.174	0.04	0
ANG	0	2.65998453	0.201	0.048	0
ZDHHC2	0	3.116347538	0.147	0.04	0
KCNQ5	0	-4.162108435	0.048	0.338	0
AC009107.2	0	2.743039334	0.181	0.053	0
ZFYVE1	0	2.685915147	0.122	0.042	0
CT83	0	-7.11053445	0.002	0.291	0
TGFB3	0	3.262482956	0.118	0.022	0
LYPD6	0	3.857418029	0.094	0.012	0
SORD	0	-2.507622588	0.102	0.485	0
SKAP1	0	2.884765163	0.155	0.034	0
EREG	0	4.548122796	0.14	0.028	0
DLX5	0	-6.615528918	0.001	0.096	0
CKAP2	0	-4.060663264	0.025	0.341	0

EFNA3	0	-3.061059054	0.043	0.371	0
CLDN23	0	-3.337814349	0.019	0.211	0
AC007402.1	0	5.53144382	0.118	0.011	0
AC119674.1	0	-2.642419343	0.087	0.401	0
FSIP1	0	3.492756602	0.092	0.014	0
AC007100.1	0	-4.011537515	0.012	0.188	0
LINC02664	0	-8.287652552	0	0.238	0
PCLAF	0	-3.77653367	0.046	0.35	0
LSAMP	0	3.545122703	0.149	0.062	0
IGHG1	0	-2.778753698	0.003	0.173	0
GLIPR1	0	3.121405775	0.351	0.146	0
CAVIN3	0	-3.253016219	0.053	0.367	0
CENPW	0	-2.567143207	0.091	0.442	0
AC092069.1	0	-4.083450007	0.011	0.075	0
TMEM156	0	3.553720941	0.097	0.014	0
OTUD1	0	3.14314369	0.121	0.02	0
TMEM211	0	-6.222082983	0.002	0.245	0
BATF3	0	3.31454044	0.125	0.029	0
RAI2	0	3.879378866	0.114	0.007	0
F3	0	3.860916825	0.227	0.036	0
SLC49A3	0	2.873075169	0.1	0.019	0
CHAC2	0	-2.802388958	0.047	0.371	0
SLC22A17	0	3.09007493	0.15	0.025	0
WDR92	0	-2.731027325	0.041	0.33	0
DYNLRB2	0	3.068976626	0.092	0.016	0
HEPACAM2	0	7.881002648	0.077	0	0
NMB	0	-2.983360059	0.107	0.431	0
AC015923.1	0	3.570235494	0.102	0.01	0
VCAN	0	-4.539977356	0.018	0.347	0
SHC3	0	-4.454044084	0.009	0.251	0
OTX1	0	-8.004546904	0.001	0.179	0
CDCA7L	0	-2.936260981	0.052	0.42	0
UCHL1	0	-7.866426006	0.004	0.364	0
LINC01122	0	-3.458068497	0.021	0.26	0
LINC01143	0	-5.310757644	0.005	0.218	0
KLF2	0	3.193030789	0.18	0.042	0
AC009501.3	0	-4.086173872	0.009	0.198	0
RFC3	0	-2.547803687	0.069	0.366	0
SYT9	0	4.698368122	0.084	0.009	0

AC007529.2	0	-3.144612432	0.025	0.279	0
TBX19	0	-2.987190086	0.028	0.312	0
MANCR	0	4.400458978	0.08	0.013	0
STAC	0	-3.894918213	0.022	0.327	0
ZNF98	0	2.969226274	0.095	0.027	0
CHRM3-AS2	0	-4.337961984	0.01	0.187	0
CALML4	0	-3.612542724	0.038	0.383	0
PDK4	0	2.620956519	0.244	0.113	0
ZWINT	0	-3.791951464	0.035	0.345	0
HLTF	0	-2.55668666	0.063	0.418	0
GREB1	0	3.009022732	0.132	0.029	0
APBB1	0	3.018295651	0.138	0.031	0
DTYMK	0	-2.830862656	0.133	0.568	0
LIN7A	0	4.229870208	0.1	0.013	0
ANKRD33B	0	4.012192431	0.114	0.019	0
CDC25B	0	-3.081623659	0.053	0.398	0
GATA6	0	2.667252085	0.096	0.035	0
ZNF483	0	2.967514722	0.137	0.037	0
AC010735.2	0	3.801789437	0.097	0.014	0
PRRT3-AS1	0	-3.14670839	0.038	0.358	0
PLAAT1	0	-3.710209535	0.027	0.386	0
MUC4	0	4.454748185	0.093	0.007	0
FOXI1	0	-2.569696534	0.02	0.164	0
AL078590.2	0	-4.671997259	0.005	0.148	0
RCAN1	0	3.036100591	0.497	0.286	0
PLA2G7	0	-5.635949419	0.003	0.284	0
TFAP4	0	-4.026304297	0.018	0.323	0
SMIM31	0	4.371702101	0.076	0.007	0
MKI67	0	-6.407976398	0.007	0.229	0
SATB1-AS1	0	-5.356679879	0.004	0.207	0
AC091729.3	0	-3.324555746	0.024	0.282	0
SNORC	0	3.770679301	0.276	0.121	0
POSTN	0	-3.507651372	0.032	0.313	0
FAM83D	0	-5.171331679	0.007	0.303	0
RPH3AL	0	3.028852471	0.077	0.01	0
CCNB2	0	-5.484697815	0.012	0.27	0
GLP1R	0	-5.423897502	0.007	0.282	0
AL161431.1	0	-11.38275312	0	0.286	0
AL390860.1	0	3.800568675	0.07	0.003	0

TCHP	0	-2.500761241	0.06	0.377	0
AC078923.1	0	3.728184783	0.148	0.033	0
FYB2	0	4.323905924	0.077	0.007	0
CEP170	0	-2.665399431	0.059	0.421	0
FAM92A	0	-2.540294422	0.038	0.268	0
ZBTB16	0	2.9969158	0.148	0.025	0
NFATC1	0	2.603370463	0.209	0.04	0
JHY	0	3.837273223	0.075	0.009	0
HMGCLL1	0	3.739657821	0.086	0.014	0
DSCC1	0	-3.583870865	0.02	0.312	0
LYZ	0	-6.07672022	0.006	0.154	0
LIME1	0	-5.095705938	0.011	0.325	0
TNFSF11	0	7.519401803	0.057	0	0
AP000547.3	0	4.449210749	0.124	0.007	0
HMOX1	0	2.616799983	0.156	0.065	0
HILPDA	0	-4.266068228	0.048	0.543	0
IGLC3	0	-3.528445575	0.007	0.158	0
RTKN2	0	-2.827543313	0.05	0.339	0
TLE2	0	2.528867001	0.248	0.083	0
AC027601.1	0	-4.426485845	0.007	0.256	0
CDH12	0	-2.95853146	0.041	0.257	0
HAGHL	0	-3.316860497	0.044	0.421	0
LARGE2	0	-2.876022646	0.055	0.419	0
RP1	0	3.424087828	0.086	0.009	0
GJB3	0	-2.96456731	0.051	0.343	0
HIST1H4C	0	-2.819924417	0.265	0.657	0
AKAP12	0	3.093544786	0.079	0.02	0
CDH11	0	-4.395536058	0.014	0.285	0
DCDC2	0	4.334539592	0.1	0.007	0
PRTFDC1	0	-4.886316211	0.009	0.292	0
ITGB2	0	-3.12814985	0.023	0.23	0
SYN3	0	-3.747360254	0.019	0.234	0
AC016576.1	0	6.539758525	0.067	0.001	0
GABRE	0	-3.992825144	0.013	0.169	0
FN1	0	-2.763526304	0.08	0.276	0
AC105094.2	0	-2.925721569	0.013	0.123	0
STK32B	0	5.284189588	0.073	0.005	0
MSANTD4	0	-3.473941317	0.023	0.311	0
C2CD4B	0	3.544008559	0.068	0.009	0

CDK1	0	-5.190714937	0.01	0.245	0
MCF2L-AS1	0	-2.901071386	0.029	0.306	0
NCAM1	0	-4.86188437	0.017	0.238	0
FAM110C	0	3.749334695	0.082	0.007	0
SAC3D1	0	-3.000372937	0.052	0.429	0
TDRD1	0	-9.03700659	0	0.154	0
ADAM19	0	2.992210531	0.088	0.023	0
LINGO1	0	-4.07382364	0.008	0.163	0
TTC13	0	-3.694159589	0.021	0.312	0
PPARGC1A	0	-4.431414058	0.006	0.138	0
AC239799.2	0	-4.061063308	0.015	0.179	0
TUBB3	0	-3.421424965	0.044	0.335	0
AP001783.1	0	-5.12769049	0.004	0.212	0
METRNL	0	2.980528365	0.101	0.028	0
BDKRB2	0	3.441388091	0.048	0.007	0
DGAT2	0	-2.781527876	0.048	0.415	0
NGB	0	-3.900923011	0.005	0.133	0
AC079336.4	0	2.804253492	0.082	0.021	0
PAPLN	0	3.325181628	0.075	0.013	0
GAS6	0	3.325912825	0.117	0.01	0
ATP6V0A4	0	-2.582576613	0.032	0.273	0
DSEL	0	3.476242804	0.117	0.029	0
AL133334.1	0	-4.531325909	0.006	0.208	0
EHMT2	0	-2.594838379	0.055	0.401	0
VIM-AS1	0	-2.863939757	0.031	0.256	0
LINC01436	0	-4.526430953	0.009	0.228	0
C16orf95	0	-3.676331093	0.03	0.353	0
AP000779.1	0	-3.512495205	0.013	0.166	0
SPON1	0	-2.792620257	0.048	0.311	0
LINC02438	0	-7.124934707	0.001	0.159	0
MKRN3	0	-8.287145827	0.001	0.26	0
PIP4P2	0	-3.49676524	0.018	0.266	0
UNG	0	-2.75821566	0.072	0.418	0
NPFFR2	0	6.409611567	0.055	0.001	0
RFC4	0	-3.234340659	0.04	0.356	0
GRIA2	0	-4.819958535	0.003	0.081	0
ALOXE3	0	5.548813453	0.063	0.003	0
LUCAT1	0	2.826330048	0.315	0.153	0
GRB14	0	-2.544714364	0.04	0.271	0

FAM184B	0	3.112801147	0.1	0.029	0
LINC02609	0	-2.80045106	0.015	0.157	0
SLC26A4-AS1	0	-2.90315555	0.016	0.169	0
GOLIM4	0	-2.751370704	0.054	0.356	0
ZNF750	0	-3.405299566	0.015	0.183	0
CDC20	0	-4.923876112	0.019	0.322	0
TLL1	0	-3.459862727	0.015	0.278	0
AP003100.1	0	-8.922487253	0	0.125	0
SH2B2	0	2.551315795	0.077	0.028	0
AC104574.2	0	4.093787344	0.055	0.006	0
FBXL16	0	-4.26309901	0.006	0.126	0
SLC35G2	0	-2.828449479	0.025	0.228	0
SLC6A11	0	-3.64590841	0.01	0.165	0
PRC1	0	-3.382743429	0.041	0.268	0
ADD2	0	-6.785047574	0.003	0.282	0
CUX2	0	3.910006391	0.074	0.015	0
MAGEA6	0	-9.562702113	0	0.201	0
KCNMB2	0	-4.594097998	0.003	0.091	0
GGT5	0	3.120904824	0.107	0.013	0
PIR	0	-3.583817666	0.022	0.289	0
AC068774.1	0	-4.905797278	0.007	0.242	0
PPP4R4	0	4.701278525	0.063	0.005	0
TSKU	0	-3.536630787	0.023	0.328	0
SMOC1	0	-3.949301883	0.017	0.277	0
C15orf41	0	-2.66573346	0.052	0.378	0
PALM	0	3.585104205	0.064	0.011	0
CEP295	0	-2.781810388	0.048	0.363	0
CLEC4F	0	-8.195632987	0	0.163	0
DNAH17	0	-2.975544237	0.014	0.17	0
HCAR2	0	4.523861763	0.121	0.014	0
S100P	0	-2.613594658	0.059	0.305	0
AC105114.2	0	-3.878749468	0.007	0.129	0
IMPDH1	0	-2.786209763	0.142	0.595	0
CITED1	0	5.286997709	0.078	0.006	0
ASPM	0	-6.317808651	0.005	0.216	0
SDK1	0	2.727542088	0.142	0.039	0
STRIP2	0	-3.943507071	0.011	0.227	0
AC021148.2	0	6.191244694	0.047	0.001	0
CXCL8	0	3.960975537	0.272	0.081	0

PDGFA	0	-3.43969466	0.031	0.306	0
SMOC2	0	2.906856932	0.082	0.021	0
TPX2	0	-5.02401433	0.011	0.248	0
ARHGEF38-IT1	0	4.509538967	0.106	0.008	0
EPSTI1	0	-3.358713595	0.018	0.194	0
LRG1	0	3.512588334	0.078	0.013	0
POMC	0	3.821729376	0.048	0.004	0
AC090644.1	0	-4.258269765	0.006	0.101	0
SLCO4A1	0	-2.916225374	0.024	0.229	0
TMEM64	0	-2.549850167	0.026	0.237	0
LINC01828	0	-6.486814267	0.002	0.229	0
NCK1-DT	0	-2.867154857	0.023	0.221	0
AC079921.1	0	3.13789831	0.116	0.017	0
SLC4A11	0	-2.75199669	0.026	0.246	0
LINGO2	0	-6.608994215	0.004	0.209	0
SRARP	0	4.284691878	0.047	0.002	0
LINC02381	0	2.962928411	0.114	0.032	0
CHI3L2	0	3.045745212	0.259	0.107	0
ORC6	0	-3.954985579	0.021	0.28	0
EVA1A	0	-2.834235945	0.05	0.295	0
AL022316.1	0	-4.991418897	0.004	0.187	0
LTF	0	3.02560366	0.298	0.066	0
PRSS27	0	-2.689540358	0.024	0.183	0
AC005162.3	0	-4.172703206	0.007	0.147	0
AC018695.9	0	-4.208218776	0.006	0.144	0
AC244102.4	0	-6.47440937	0.002	0.186	0
BAG2	0	-3.449984113	0.022	0.29	0
AC092171.3	0	-2.880945833	0.013	0.134	0
MYPN	0	4.087061281	0.064	0.007	0
KCNK2	0	-3.590209091	0.009	0.146	0
TRIM58	0	4.351747944	0.052	0.004	0
GPR180	0	-2.508667648	0.039	0.275	0
SYTL5	0	4.289166606	0.055	0.005	0
AC100801.1	0	3.838474826	0.072	0.011	0
AC127526.1	0	4.153976863	0.057	0.008	0
NRXN3	0	2.674054946	0.097	0.039	0
RAD51AP1	0	-4.343603845	0.015	0.251	0
NECAB1	0	-5.755862238	0.002	0.149	0

AC105094.1	0	-6.011462142	0.001	0.051	0
FAM217B	0	-3.075254557	0.022	0.268	0
MAD2L1	0	-3.556636045	0.026	0.264	0
TMEM182	0	-2.549164857	0.027	0.221	0
CYP24A1	0	2.752754065	0.226	0.093	0
LRP8	0	-3.526020479	0.022	0.303	0
GLYATL1	0	-3.405705144	0.014	0.147	0
AC108734.4	0	-4.995945196	0.003	0.069	0
GPR12	0	-3.345558055	0.007	0.078	0
ASF1B	0	-4.10781893	0.02	0.285	0
CCDC74B	0	3.742483348	0.055	0.008	0
MICU3	0	3.2807579	0.059	0.007	0
PTTG1	0	-3.136742699	0.058	0.321	0
AC036176.3	0	-3.11997595	0.005	0.1	0
SYT1	0	-3.717023841	0.014	0.237	0
RUNX2	0	-2.848856912	0.022	0.186	0
AC087762.1	0	3.086701889	0.124	0.031	0
AC004083.1	0	-4.207157704	0.006	0.172	0
LINC01833	0	-4.898402717	0.003	0.067	0
DZIP1	0	-4.812839794	0.005	0.205	0
LINC01214	0	3.539300974	0.061	0.01	0
IGSF1	0	3.446160284	0.061	0.01	0
ITPRID1	0	-3.96341765	0.004	0.119	0
AC005808.1	0	-4.276980008	0.003	0.109	0
AC021723.2	0	6.476921057	0.04	0.001	0
AC092535.4	0	3.197796225	0.054	0.009	0
NUSAP1	0	-3.338634139	0.047	0.28	0
AC104248.1	0	-4.81477495	0.004	0.15	0
MLKL	0	-2.784704579	0.021	0.231	0
AKR1C1	0	-4.107759162	0.013	0.199	0
ANXA9	0	3.626071225	0.068	0.01	0
ASPG	0	-4.528962704	0.003	0.067	0
ZNF365	0	-4.204686395	0.01	0.201	0
IFI44L	0	-3.130984249	0.017	0.167	0
SAMHD1	0	-3.249887842	0.018	0.179	0
NLRP1	0	-4.958048229	0.014	0.287	0
AC010789.1	0	-7.661622231	0.001	0.198	0
CNTD1	0	2.894091269	0.096	0.033	0
SPART-AS1	0	3.762657301	0.058	0.009	0

MUC16	0	-2.927116427	0.023	0.246	0
HIST1H1A	0	-7.332659927	0.001	0.156	0
COL9A3	0	-4.461351433	0.012	0.317	0
GRIK1	0	3.454135231	0.121	0.024	0
ARTN	0	3.276692872	0.056	0.016	0
FAM81B	0	2.776041159	0.039	0.002	0
AURKA	0	-5.248640428	0.006	0.219	0
DLC1	0	2.647397027	0.087	0.02	0
C1QTNF1	0	-2.583782065	0.056	0.302	0
RGS9	0	2.546235056	0.062	0.013	0
AC006329.1	0	-2.866918457	0.03	0.244	0
TEDC1	0	-3.262577924	0.03	0.276	0
WDR86-AS1	0	-2.559180765	0.02	0.199	0
DEPDC1	0	-6.713352193	0.002	0.18	0
SOX15	0	-3.195802706	0.017	0.267	0
ZNF341-AS1	0	3.149573671	0.094	0.021	0
DLX6-AS1	0	-7.9883828	0	0.048	0
BATF2	0	-3.944781508	0.008	0.149	0
ETV7	0	-4.035075128	0.018	0.266	0
FOXP4-AS1	0	-2.944876653	0.025	0.269	0
NSMCE4A	0	-3.799216139	0.185	0.59	0
PTGER3	0	4.10425174	0.053	0.003	0
KCNIP4	0	-4.675136799	0.013	0.122	0
C2CD4A	0	2.984958398	0.065	0.02	0
EN1	0	-6.055366832	0.002	0.114	0
MGAM2	0	4.66654184	0.077	0.008	0
PLCXD2	0	2.616958952	0.054	0.017	0
MSI1	0	4.433071849	0.06	0.006	0
SMC2	0	-2.517207351	0.07	0.371	0
ENC1	0	-3.263561512	0.017	0.259	0
TTYH3	0	-2.581926113	0.042	0.345	0
CDCA8	0	-5.257568834	0.009	0.233	0
SGO1	0	-5.356159766	0.006	0.223	0
AC053513.1	0	-2.779282793	0.015	0.161	0
PRAME	0	-8.608538379	0.001	0.218	0
MIR924HG	0	-3.091711745	0.045	0.26	0
RRM2	0	-4.097269472	0.014	0.221	0
AC019197.1	0	-4.326140878	0.005	0.126	0
NUF2	0	-4.859971955	0.008	0.192	0

IRX6	0	-4.722758793	0.001	0.054	0
HYMAI	0	3.598259004	0.06	0.005	0
AC020659.1	0	-2.835116774	0.022	0.215	0
AMN	0	-3.905337144	0.017	0.258	0
NRIP3	0	3.103548516	0.064	0.016	0
MT1A	0	3.695617155	0.092	0.013	0
KNSTRN	0	-3.088115632	0.039	0.289	0
CSF3R	0	3.037142188	0.083	0.015	0
BRCA2	0	-3.527860056	0.023	0.256	0
GPT2	0	-2.864570517	0.035	0.329	0
LIX1L	0	-2.818912724	0.019	0.198	0
CDKN2C	0	-3.672378295	0.01	0.165	0
NAALADL2-AS2	0	-2.960904397	0.014	0.114	0
KLHDC7B	0	-2.862730833	0.033	0.245	0
MT1M	0	3.575721741	0.1	0.008	0
PIFO	0	2.957018194	0.076	0.013	0
GFRA1	0	3.697995347	0.059	0.007	0
SEMA6D	0	-2.915542736	0.01	0.094	0
RMI2	0	-3.02937137	0.025	0.264	0
MPRIP-AS1	0	2.595345381	0.084	0.01	0
MAGEA3	0	-8.098634343	0	0.054	0
AC093627.4	0	-3.360623071	0.009	0.148	0
NOXO1	0	-3.249887091	0.01	0.107	0
PPP1R14B-AS1	0	-2.854842565	0.041	0.292	0
AP003100.2	0	-6.840128088	0.001	0.096	0
SHCBP1	0	-5.444308012	0.005	0.211	0
ZWILCH	0	-2.579543931	0.045	0.302	0
ABCC8	0	4.134329895	0.046	0.006	0
ST7-AS1	0	3.969688591	0.061	0.005	0
ADCY7	0	-3.157989517	0.021	0.275	0
A2ML1	0	-4.75946818	0.004	0.175	0
SLC2A13	0	-2.869751235	0.027	0.204	0
APELA	0	5.976064661	0.029	0.001	0
DUOXA1	0	-2.815206128	0.01	0.066	0
TMPO	0	-2.579226626	0.048	0.339	0
SLC16A2	0	3.1570203	0.068	0.01	0
VSIG10L2	0	2.951724318	0.054	0.005	0

LINC00920	0	-3.768609097	0.008	0.144	0
VGLL1	0	-3.845775604	0.015	0.236	0
ARHGAP40	0	3.324326	0.056	0.01	0
AC103770.1	0	-7.603307138	0.001	0.15	0
ARHGAP11A	0	-5.860636969	0.005	0.196	0
CDCA7	0	-5.234715776	0.006	0.24	0
HTN1	0	-9.912204384	0	0.033	0
CLEC2L	0	-5.436865948	0.002	0.176	0
NEXMIF	0	-2.671362161	0.012	0.107	0
CXCL13	0	2.511373062	0.07	0.028	0
AL445253.1	0	2.523385274	0.065	0.023	0
SLC16A6	0	3.778723875	0.052	0.008	0
TMEM217	0	3.51351766	0.077	0.016	0
CYP39A1	0	-3.195862131	0.015	0.208	0
RPUSD1	0	-2.748578036	0.066	0.42	0
LINC01806	0	-3.081536448	0.008	0.116	0
CCNB1	0	-3.466612112	0.038	0.256	0
HR	0	-3.334345811	0.011	0.195	0
ADAP1	0	-3.92269924	0.014	0.281	0
TRIM59	0	-2.574158337	0.029	0.234	0
FAM189A2	0	2.513418303	0.166	0.07	0
AQP1	0	-2.576275686	0.051	0.237	0
AC106799.2	0	-4.785029055	0.006	0.172	0
VIT	0	-3.156395273	0.01	0.114	0
FAM167B	0	-3.771532653	0.013	0.199	0
OVCH1-AS1	0	5.483809857	0.059	0.003	0
LINC01950	0	-4.144595899	0.002	0.059	0
CSAG1	0	-9.193835828	0	0.144	0
PCDHGA10	0	-3.525896785	0.006	0.105	0
LINC00887	0	-4.441408857	0.003	0.125	0
CENPJ	0	-3.326362265	0.024	0.254	0
NWD1	0	-4.376391176	0.004	0.103	0
TSHZ3	0	3.573508199	0.053	0.01	0
TRAF1	0	2.879998488	0.099	0.033	0
POGLUT3	0	-3.304855768	0.018	0.24	0
PLK1	0	-5.923464939	0.004	0.183	0
GPX8	0	-3.285001776	0.024	0.243	0
AC008537.4	0	-3.458784915	0.009	0.084	0
NDC80	0	-5.72092706	0.004	0.179	0

GATA3-AS1	0	2.901310042	0.093	0.026	0
DLGAP1-AS5	0	-3.486694356	0.007	0.129	0
NAIP	0	2.692333282	0.07	0.019	0
AL008719.1	0	-5.438879182	0.001	0.045	0
AL139393.3	0	3.101328235	0.061	0.014	0
AL136456.1	0	-4.17422568	0.006	0.126	0
CENPK	0	-3.409809169	0.02	0.231	0
XAF1	0	-2.849715227	0.017	0.187	0
AP000721.2	0	-2.611615169	0.015	0.087	0
AC087071.2	0	-2.62999789	0.014	0.08	0
LRRC32	0	-4.497644917	0.007	0.076	0
GPR146	0	-2.824334872	0.011	0.135	0
PLAG1	0	-3.542713069	0.007	0.111	0
TRIM9	0	-2.565032029	0.012	0.163	0
CCNA2	0	-5.103902642	0.005	0.159	0
LPAR5	0	-3.190022675	0.003	0.072	0
LINC01087	0	6.485158151	0.028	0	0
SLC66A1L	0	-3.293254057	0.009	0.144	0
SAMD1	0	-2.99058255	0.034	0.326	0
AC018695.2	0	-4.661069589	0.004	0.099	0
BDNF	0	-3.721196088	0.005	0.104	0
IL7	0	-2.959657515	0.018	0.182	0
TSPAN2	0	-3.8909754	0.008	0.174	0
PCAT7	0	-3.016754079	0.013	0.165	0
PRKCQ-AS1	0	-2.688968256	0.018	0.171	0
CCL14	0	5.519431424	0.06	0.003	0
HIST1H1E	0	-4.832566451	0.006	0.153	0
PACRG	0	2.623580218	0.065	0.014	0
HEATR3	0	-2.872826048	0.018	0.194	0
AC093523.1	0	-4.668582639	0.003	0.124	0
C11orf96	0	3.686986991	0.068	0.013	0
KRT83	0	-4.888319881	0.002	0.05	0
PBK	0	-3.657733699	0.007	0.162	0
UGT8	0	-2.569233566	0.02	0.178	0
AC010329.1	0	2.679596537	0.073	0.02	0
SIX3	0	-4.342407612	0.002	0.052	0
MTBP	0	-3.032671062	0.018	0.221	0
CLIC3	0	-3.291677298	0.018	0.162	0
CA11	0	2.525755103	0.085	0.02	0

TF	0	2.637292318	0.069	0.015	0
MOCS1	0	3.047251767	0.067	0.022	0
CDCA3	0	-5.353275479	0.005	0.188	0
CDCA5	0	-4.507371	0.01	0.222	0
PICSAR	0	-6.623762851	0.001	0.174	0
TRIP13	0	-4.05866319	0.021	0.291	0
CCDC68	0	3.032910864	0.04	0.01	0
GPER1	0	-4.110111413	0.006	0.189	0
GPC5	0	-3.034383721	0.013	0.115	0
SYCE1	0	6.450815513	0.034	0	0
AL596087.2	0	-3.275740229	0.006	0.044	0
AC008663.1	0	6.878804324	0.035	0	0
AL358232.2	0	-2.682893733	0.026	0.226	0
RASGRF1	0	-5.078534182	0.003	0.151	0
HS3ST4	0	4.114238484	0.06	0.005	0
LINC01764	0	-4.465376591	0.003	0.088	0
AL121578.3	0	4.487608007	0.031	0.002	0
AL035401.1	0	-2.9142354	0.014	0.108	0
CNTN4	0	2.720023921	0.095	0.017	0
KLHL35	0	-2.599578396	0.028	0.217	0
AC243967.2	0	-2.696544224	0.009	0.067	0
ZNF385B	0	3.774435653	0.088	0.033	0
KIF23	0	-3.892054078	0.021	0.252	0
BAALC	0	4.030019944	0.04	0.004	0
CYP1A1	0	6.944437215	0.034	0.001	0
HPDL	0	-4.211888505	0.008	0.143	0
TACC3	0	-3.769715403	0.011	0.206	0
IDO1	0	-3.140965523	0.014	0.106	0
SOBP	0	-3.046540649	0.009	0.19	0
TNRC6C-AS1	0	-3.864387779	0.005	0.106	0
FAM111B	0	-3.904759619	0.009	0.12	0
SCN2A	0	-4.49982281	0.003	0.099	0
MARC1	0	-2.982343398	0.035	0.311	0
BFSP1	0	-2.63789253	0.027	0.217	0
PKMYT1	0	-4.366607822	0.007	0.2	0
LCT	0	-2.739184891	0.009	0.119	0
AL592156.2	0	-2.566785042	0.014	0.096	0
C1QTNF1-AS1	0	-3.475219804	0.011	0.183	0
AC017083.1	0	-3.847347058	0.004	0.14	0

AL627171.1	0	-2.637212929	0.016	0.142	0
AC007563.2	0	-2.787969053	0.008	0.082	0
CENPM	0	-3.067495411	0.035	0.272	0
LINC00639	0	-5.19918277	0.003	0.175	0
BMPR1B	0	-2.676548278	0.026	0.25	0
NINJ2	0	-3.556868841	0.007	0.188	0
AC106795.2	0	2.964355239	0.029	0.004	0
CCL20	0	3.696496696	0.142	0.046	0
AC103796.1	0	-4.74404502	0.002	0.099	0
HNF4G	0	2.809255498	0.073	0.024	0
TEDC2	0	-4.170228644	0.007	0.168	0
IGFL2	0	8.075241437	0.031	0	0
XRCC3	0	-3.68836451	0.01	0.185	0
FLVCR1-DT	0	-3.145496871	0.009	0.17	0
NCAM2	0	2.644722057	0.068	0.018	0
B3GNT8	0	3.370456845	0.04	0.004	0
AC063960.1	0	-4.379398747	0.004	0.094	0
LINC00471	0	-4.1665559	0.003	0.114	0
TOP2A	0	-4.044255199	0.01	0.181	0
SH2D4A	0	-2.65008003	0.02	0.213	0
CHEK2	0	-2.627723465	0.03	0.227	0
MECOM	0	-2.788315533	0.067	0.384	0
LINC02616	0	-8.349313	0	0.146	0
TMEM88	0	2.55483609	0.042	0.015	0
AC090236.2	0	3.638078661	0.038	0.006	0
AC108860.2	0	-2.791993427	0.012	0.125	0
CHEK1	0	-2.985688823	0.028	0.267	0
CCDC85A	0	-3.750116628	0.011	0.147	0
LINC00839	0	-3.079686382	0.011	0.125	0
TROAP	0	-4.811803979	0.005	0.148	0
DIAPH3	0	-3.092045765	0.035	0.256	0
CMPK2	0	-3.841533742	0.006	0.106	0
SLC9C1	0	2.913092881	0.065	0.016	0
SKP2	0	-4.301698524	0.007	0.212	0
AC008945.2	0	-3.616841472	0.012	0.127	0
AP001207.3	0	-2.662989968	0.062	0.343	0
VGF	0	-4.502823401	0.005	0.151	0
MAGEA12	0	-8.448772107	0	0.1	0
ALKBH8	0	-2.885994422	0.021	0.229	0

WNK4	0	3.815099567	0.033	0.003	0
KIRREL3-AS1	0	-4.901448603	0.003	0.151	0
CCNO	0	-3.398388751	0.014	0.256	0
AC008268.1	0	6.702664153	0.029	0	0
SLCO1B3	0	-9.12729013	0	0.093	0
FOXM1	0	-3.929545471	0.011	0.196	0
LINC01910	0	-6.573562133	0.001	0.095	0
SCN3A	0	-4.375513908	0.002	0.06	0
ADGRG6	0	-3.347324864	0.01	0.151	0
AC003101.2	0	6.072551158	0.039	0.001	0
ADRA2A	0	4.623894899	0.033	0.002	0
AMBP	0	5.147789393	0.04	0.003	0
TRMT9B	0	2.747815768	0.056	0.014	0
GINS1	0	-4.096581263	0.008	0.164	0
UGT2B15	0	4.806768902	0.033	0.005	0
OIP5	0	-3.442467512	0.013	0.203	0
PRSS12	0	-3.759554306	0.009	0.159	0
SLC16A11	0	3.372955167	0.06	0.006	0
BUB1B	0	-4.871603479	0.004	0.148	0
LINC02649	0	2.880648974	0.052	0.009	0
NPR3	0	-3.694887672	0.004	0.068	0
AL139220.2	0	-2.733710686	0.024	0.253	0
DLGAP1-AS3	0	-3.750509772	0.006	0.092	0
PRTG	0	2.59094436	0.046	0.014	0
EXOC3L1	0	-2.96111853	0.007	0.073	0
FIGNL1	0	-3.323810752	0.01	0.127	0
FLT3	0	2.586237358	0.031	0.009	0
DLGAP1-AS4	0	-4.197822602	0.003	0.06	0
ITGBL1	0	3.025623207	0.084	0.03	0
HMMR	0	-3.500659199	0.015	0.141	0
AC009053.3	0	-4.045393029	0.003	0.054	0
RASD2	0	-4.206745875	0.002	0.059	0
CPAMD8	0	2.690747932	0.148	0.055	0
CTHRC1	0	-3.703287674	0.026	0.261	0
CDCA2	0	-3.64026285	0.013	0.2	0
FBXO41	0	-3.345494626	0.011	0.154	0
GVQW3	0	-3.269552531	0.009	0.147	0
BPIFB1	0	3.668154411	0.049	0.008	0
LINC01703	0	-2.667169929	0.016	0.15	0

AC019117.2	0	-3.616385743	0.007	0.117	0
AC008011.2	0	5.107770805	0.036	0.002	0
IL17RE	0	-2.521115157	0.033	0.26	0
PALM3	0	-5.252647043	0.003	0.165	0
BCL11B	0	-4.026606243	0.007	0.154	0
CCK	0	4.845803491	0.026	0.001	0
CPVL	0	-4.693020651	0.005	0.142	0
LCN12	0	3.263651592	0.04	0.008	0
ADAMTS12	0	3.383405325	0.029	0.005	0
SPINK8	0	-2.724760591	0.049	0.126	0
ALB	0	5.29651236	0.051	0.004	0
LIN9	0	-2.656912163	0.025	0.224	0
MDGA2	0	-4.366047379	0.006	0.14	0
CILP	0	-3.584806314	0.004	0.039	0
AC010336.1	0	-2.857761204	0.005	0.082	0
LARGE-AS1	0	3.117346781	0.041	0.012	0
PLCB2	0	-5.051526295	0.003	0.093	0
FLT1	0	-2.999042444	0.022	0.182	0
NR2E3	0	2.541868199	0.054	0.018	0
AC005264.1	0	4.745386867	0.037	0.002	0
GAS1	0	-2.871734649	0.01	0.133	0
PLBD1-AS1	0	-3.324204787	0.008	0.126	0
AC090541.1	0	4.072422921	0.029	0.004	0
ZMYND15	0	2.650207558	0.096	0.03	0
NKX2-8	0	5.083320076	0.021	0.001	0
HPSE	0	-4.535992818	0.001	0.085	0
TAF1A	0	-2.784573524	0.016	0.201	0
JAG2	0	-3.228837677	0.009	0.14	0
NEK2	0	-5.945658074	0.003	0.157	0
FGL1	0	4.324153227	0.031	0.002	0
C21orf58	0	-3.987242494	0.009	0.164	0
LINC02036	0	-2.840119384	0.006	0.07	0
GRIK3	0	5.728378019	0.038	0.002	0
LRRC45	0	-2.763041639	0.033	0.29	0
AC016682.1	0	5.997955628	0.021	0	0
CYP2A7	0	5.764906806	0.019	0	0
PSPH	0	-3.028497673	0.025	0.264	0
RAD51	0	-3.083915391	0.017	0.17	0
DPP4	0	-4.019969546	0.011	0.198	0

AC110491.2	0	-5.162810861	0.001	0.031	0
RNF212	0	4.923931995	0.039	0.003	0
TBILA	0	4.549062674	0.025	0.001	0
SNPH	0	4.725603356	0.036	0.002	0
GPX3	0	2.754462707	0.046	0.008	0
ZAN	0	-3.115974992	0.005	0.039	0
GINS3	0	-2.766012097	0.017	0.165	0
MIR155HG	0	3.072371897	0.082	0.02	0
HJURP	0	-6.18695017	0.002	0.139	0
AL442003.1	0	-2.606980736	0.011	0.073	0
C8orf37	0	-2.536439929	0.011	0.136	0
AC015922.3	0	2.514892641	0.054	0.02	0
F5	0	-3.358385823	0.003	0.045	0
AC068286.2	0	-3.439375519	0.004	0.059	0
SPC24	0	-2.638640619	0.018	0.146	0
NDN	0	-2.938418527	0.01	0.163	0
ALKAL2	0	5.559580033	0.049	0.002	0
HIST1H1D	0	-4.288318161	0.007	0.114	0
TTK	0	-5.471405006	0.002	0.129	0
CST6	0	-2.928513987	0.035	0.178	0
PRR11	0	-4.527628903	0.005	0.139	0
MUC5B	0	-2.54985517	0.014	0.1	0
ENPEP	0	-4.000220249	0.004	0.043	0
PTPRC	0	-3.447996937	0.007	0.089	0
MTRNR2L1	0	3.347892071	0.097	0.022	0
AL132712.1	0	-2.666158688	0.007	0.073	0
AC093627.1	0	-8.335816598	0	0.103	0
TUBB2B	0	4.3100723	0.056	0.005	0
RHEX	0	-4.02670258	0.005	0.141	0
LINC02532	0	-4.914751462	0.016	0.158	0
FIBIN	0	-3.409999827	0.006	0.068	0
COX7A1	0	4.235509579	0.071	0.004	0
DNAAF1	0	2.609774172	0.047	0.012	0
CEP152	0	-2.98657104	0.021	0.23	0
CLSPN	0	-4.14681227	0.011	0.21	0
PCDHB9	0	-3.683893155	0.005	0.109	0
CCL2	0	2.932675225	0.244	0.119	0
CFAP157	0	2.789122764	0.03	0.012	0
CSF3	0	7.781196107	0.054	0	0

AC092839.1	0	-2.863502721	0.008	0.064	0
LINC00970	0	-2.959762689	0.007	0.061	0
KNL1	0	-4.343287167	0.012	0.187	0
MS4A7	0	2.980126217	0.029	0.01	0
CFAP46	0	3.790742277	0.032	0.003	0
NDRG4	0	-2.748481928	0.009	0.125	0
CLDN5	0	5.390120599	0.037	0.003	0
AC138512.1	0	3.15118749	0.049	0.014	0
AC133919.1	0	-2.553422373	0.007	0.058	0
ALDH1L2	0	-3.137025566	0.012	0.147	0
ADGRG5	0	-5.214815388	0.002	0.08	0
SIAH2-AS1	0	-2.593428915	0.015	0.13	0
GAS1RR	0	-3.396008451	0.004	0.086	0
AC133065.1	0	-4.788776772	0.001	0.039	0
TMEM132E	0	-6.67872355	0	0.084	0
ARHGAP11B	0	-5.609608591	0.005	0.201	0
ERICH5	0	-3.923612687	0.005	0.094	0
EFCAB10	0	-2.604872854	0.017	0.143	0
LPCAT4	0	-3.00438587	0.014	0.192	0
MCM2	0	-3.529797667	0.012	0.179	0
SPOCD1	0	3.784657177	0.037	0.006	0
CYP4F22	0	2.723911351	0.046	0.005	0
CHST11	0	-2.573980456	0.025	0.25	0
CNKSR2	0	-3.137871014	0.007	0.125	0
AL162595.1	0	-2.691613517	0.011	0.115	0
F11-AS1	0	-3.740012986	0.005	0.117	0
EML2-AS1	0	-4.537620397	0.002	0.054	0
RADIL	0	-3.007964693	0.015	0.214	0
AL355304.1	0	3.590539454	0.06	0.011	0
RRAD	0	2.812664206	0.106	0.043	0
SMPX	0	-3.594892149	0.002	0.033	0
NKX1-2	0	-5.635940726	0.001	0.149	0
NGEF	0	-2.724994905	0.017	0.17	0
ARMCX2	0	3.091735321	0.057	0.013	0
AC129926.1	0	3.197773875	0.021	0.005	0
MNS1	0	-2.526497046	0.048	0.249	0
AC124283.1	0	-3.321402546	0.007	0.051	0
LINC01208	0	-2.758296383	0.017	0.127	0
TMEM26	0	4.85020491	0.033	0.001	0

RSAD2	0	-2.555810728	0.012	0.059	0
TRAV13-2	0	5.484548826	0.017	0	0
AL161908.1	0	2.757190654	0.035	0.008	0
MMACHC	0	-2.698051162	0.012	0.092	0
LMNB1	0	-4.296550448	0.016	0.247	0
ACKR1	0	2.795930462	0.063	0.011	0
AC108519.1	0	2.531682833	0.036	0.019	0
PCDH9-AS2	0	-4.627675909	0.001	0.041	0
RBM20	0	-3.58368559	0.004	0.053	0
ACMSD	0	4.507756845	0.026	0.002	0
FRMPD2	0	4.047077986	0.03	0.002	0
ADAMTS6	0	-4.021412034	0.004	0.082	0
AL591845.1	0	4.181043468	0.029	0.002	0
LINC01715	0	-3.142071633	0.005	0.07	0
SPIN4	0	-3.529367725	0.007	0.125	0
AC087241.2	0	-2.674304427	0.004	0.077	0
OLFM1	0	2.870947022	0.019	0.002	0
PCDHB2	0	-2.968970318	0.009	0.113	0
CNGB1	0	-4.467089836	0.003	0.095	0
AC090527.1	0	5.741920768	0.021	0.001	0
AURKB	0	-4.089450474	0.009	0.177	0
CHRDL2	0	2.943371808	0.052	0.012	0
RNASEH2A	0	-3.317378929	0.014	0.169	0
SHISA6	0	2.924419762	0.041	0.012	0
AL157778.1	0	-6.230372615	0.001	0.071	0
ACAN	0	-4.176743747	0.003	0.103	0
AL133342.1	0	5.337250121	0.017	0	0
AC026347.1	0	-2.955058458	0.009	0.087	0
AC090692.1	0	-7.500039117	0	0.061	0
AL109976.1	0	-4.529283226	0.002	0.072	0
LINC01276	0	-3.13888966	0.005	0.067	0
AC106729.1	0	3.562855738	0.026	0.002	0
MFAP5	0	-4.066619359	0.009	0.091	0
KIF2C	0	-4.487830644	0.007	0.161	0
SLC6A15	0	-4.788447647	0.004	0.14	0
FANCI	0	-2.509707541	0.029	0.221	0
AC006230.1	0	-2.900982174	0.005	0.071	0
SEMA3B-AS1	0	6.021472119	0.02	0	0
AC134312.5	0	-5.943343959	0	0.089	0

BOLA3-AS1	0	-2.775788249	0.021	0.214	0
AC079298.3	0	3.023351791	0.05	0.01	0
MGAT3	0	-2.562533012	0.004	0.033	0
SOX30	0	4.704290645	0.021	0.001	0
BORA	0	-2.951247573	0.013	0.154	0
FRGCA	0	-4.196804861	0.005	0.155	0
GIPR	0	2.890970402	0.034	0.011	0
AC099552.5	0	-3.03011296	0.005	0.088	0
AC002310.1	0	-2.523118974	0.014	0.135	0
LSAMP-AS1	0	4.639582296	0.016	0.001	0
SGO2	0	-2.732699488	0.022	0.185	0
AC117386.1	0	3.79005945	0.026	0.003	0
DLGAP5	0	-4.472347641	0.004	0.104	0
KCTD21-AS1	0	-2.636532236	0.01	0.114	0
AC093627.5	0	-2.739866976	0.008	0.069	0
INCENP	0	-2.812085479	0.014	0.142	0
MMP17	0	2.866094256	0.022	0.007	0
AC015813.1	0	2.599284167	0.033	0.013	0
AL590560.3	0	-3.133567597	0.004	0.052	0
GABRA3	0	-4.537338288	0.002	0.11	0
CYB5RL	0	-2.694585355	0.013	0.15	0
AC073934.1	0	-2.552015181	0.006	0.091	0
WNT11	0	-6.605854338	0.001	0.135	0
E2F8	0	-6.045463751	0.001	0.089	0
HENMT1	0	-2.98181975	0.029	0.231	0
ACSM1	0	3.55793346	0.035	0.004	0
HLA-DOA	0	-5.865956379	0.001	0.061	0
OPRK1	0	-5.356662521	0.001	0.039	0
TYROBP	0	-2.825786308	0.009	0.079	0
ZNF860	0	-2.717708471	0.01	0.093	0
GTSE1	0	-4.877335361	0.008	0.158	0
HLA-DOB	0	-2.855736538	0.011	0.117	0
AL928654.1	0	-3.47306874	0.005	0.072	0
AC002456.1	0	-3.744019667	0.005	0.138	0
PARPBP	0	-3.276977199	0.013	0.164	0
BUB1	0	-5.039948509	0.003	0.102	0
LRRC20	0	-3.660068174	0.006	0.12	0
DDIAS	0	-4.938608718	0.004	0.137	0
KIF18A	0	-3.360133738	0.009	0.147	0

TMEM200C	0	-5.352170601	0.001	0.076	0
AC092071.1	0	5.003286203	0.011	0	0
DNA2	0	-2.586854571	0.021	0.167	0
GPR83	0	-7.206935266	0	0.052	0
GSEC	0	-3.655439166	0.008	0.17	0
AL731571.1	0	-2.535547065	0.006	0.064	0
QPCT	0	2.9165932	0.074	0.018	0
TYMS	0	-3.141350254	0.033	0.255	0
CENPE	0	-2.701420626	0.027	0.152	0
AGTR1	0	4.982448123	0.03	0.002	0
AC092802.1	0	-3.078074832	0.005	0.047	0
KCND3	0	2.791327872	0.039	0.008	0
AC110491.3	0	-5.352068988	0	0.021	0
CXCL9	0	-5.901964876	0	0.047	0
IFIT3	0	-2.735435658	0.02	0.09	0
P3H2-AS1	0	-2.774803001	0.009	0.103	0
AC024559.1	0	-5.297528984	0.001	0.056	0
MCM10	0	-4.53311399	0.005	0.13	0
AL451042.1	0	3.127231138	0.053	0.016	0
HOXC13	0	-3.19518423	0.003	0.079	0
CT69	0	-3.188560274	0.002	0.037	0
AP000769.6	0	-4.753718063	0.001	0.042	0
LINC02136	0	-2.731517673	0.006	0.045	0
IGSF21	0	2.648542724	0.041	0.003	0
LETM2	0	3.361862758	0.031	0.006	0
CDC6	0	-3.551310343	0.01	0.144	0
GSC	0	2.532889645	0.022	0.003	0
AC009315.1	0	3.55060082	0.021	0.003	0
KCNQ4	0	-2.552593546	0.009	0.112	0
ZNF385D	0	-2.965910373	0.073	0.279	0
KIF4A	0	-3.935151726	0.005	0.123	0
AC093866.1	0	3.8929399	0.023	0.005	0
HGF	0	3.496819959	0.017	0.003	0
CNTD2	0	-2.72513829	0.01	0.118	0
AL356108.1	0	-4.201357435	0.001	0.048	0
THNSL1	0	-3.010032281	0.006	0.085	0
SOX17	0	6.201482674	0.016	0	0
LINC01010	0	-2.748114219	0.006	0.052	0
TGFBI	0	-2.805055333	0.037	0.183	0

LINC00534	0	-2.984283546	0.005	0.049	0
AC008760.2	0	-3.792515339	0.003	0.073	0
AC016745.1	0	-3.441314474	0.005	0.124	0
LINC00637	0	-2.62322227	0.006	0.047	0
MAP10	0	-3.722817772	0.004	0.076	0
CALB1	0	-8.778638604	0	0.031	0
EP300-AS1	0	-3.044708866	0.012	0.12	0
CDC45	0	-4.133061341	0.008	0.177	0
AL163051.1	0	-2.662723864	0.012	0.11	0
AC069148.1	0	-3.280720136	0.005	0.071	0
LINC02160	0	-6.606800852	0	0.019	0
AL512598.1	0	-4.575470279	0.001	0.028	0
HLA-DQB2	0	-4.163960704	0.001	0.058	0
SLC16A8	0	-3.234781379	0.004	0.072	0
SLC5A4-AS1	0	-3.908272442	0.003	0.077	0
SERF1A	0	3.172687102	0.033	0.008	0
C9orf40	0	-2.665868985	0.032	0.271	0
SH2D2A	0	-3.328021562	0.006	0.088	0
HTRA1	0	2.854486851	0.044	0.013	0
ZNF334	0	2.997720272	0.024	0.005	0
FREM2	0	-4.875352188	0.003	0.137	0
LRRC3	0	4.064175169	0.019	0.002	0
CRYBB2	0	-3.208100755	0.009	0.13	0
HRCT1	0	-3.570282465	0.005	0.113	0
AL450992.3	0	-3.324398873	0.004	0.076	0
AC020907.1	0	-2.997910453	0.007	0.089	0
CAND2	0	-3.043601014	0.005	0.043	0
CKAP2L	0	-4.450605662	0.003	0.1	0
CBX8	0	-2.629491605	0.009	0.092	0
SIX1	0	-3.969717554	0.005	0.146	0
MESP2	0	-3.961400084	0.006	0.133	0
AC104459.1	0	2.644548869	0.021	0.008	0
RNF139-AS1	0	-2.503696017	0.01	0.09	0
ELFN1-AS1	0	-6.816688108	0.001	0.053	0
LINC02269	0	-5.482481374	0.001	0.051	0
IRX4	0	-2.669625503	0.023	0.221	0
DNMBP-AS1	0	2.985076903	0.028	0.003	0
GRIK1-AS1	0	6.122928055	0.017	0	0
RGS22	0	2.536979771	0.022	0.009	0

LFNG	0	-3.231157401	0.009	0.131	0
AL356413.1	0	-4.937456543	0.001	0.023	0
COPZ2	0	2.503385917	0.026	0.007	0
C1QTNF7	0	-3.346215916	0.004	0.061	0
AC020656.1	0	-3.019135685	0.005	0.027	0
NTM-AS1	0	-7.04718317	0	0.069	0
AUNIP	0	-3.964601229	0.005	0.155	0
OR10G2	0	-6.967105126	0	0.044	0
AC051619.4	0	-3.221511652	0.003	0.04	0
ANKFN1	0	2.608072777	0.029	0.012	0
HOXA9	0	-3.001054027	0.027	0.105	0
LINC02447	0	2.855320061	0.022	0.004	0
NFATC4	0	2.55061686	0.025	0.009	0
HES6	0	-3.017893625	0.035	0.297	0
PMFBP1	0	-3.505890863	0.003	0.046	0
EML1	0	-2.895127809	0.018	0.17	0
ASPRV1	0	-3.998671327	0.003	0.05	0
OPA1-AS1	0	-3.101080802	0.005	0.058	0
GPRIN3	0	-3.714498311	0.004	0.067	0
AL365259.1	0	-2.860810425	0.004	0.043	0
AC087521.2	0	-2.582467937	0.006	0.038	0
SLC26A7	0	-3.423157026	0.002	0.065	0
CFAP73	0	-3.295103579	0.002	0.03	0
MAPT-AS1	0	3.46357669	0.023	0.004	0
NT5DC4	0	-5.621655667	0	0.039	0
DKK1	0	-7.182346438	0.004	0.253	0
AC023905.1	0	-4.83319003	0.001	0.035	0
NR2F1-AS1	0	-3.948427388	0.006	0.18	0
C5AR2	0	3.343759867	0.024	0.006	0
DSCR8	0	4.850281787	0.012	0	0
AC021242.2	0	-6.307531831	0	0.023	0
ZIC1	0	-3.261085738	0.023	0.255	0
NCAPG	0	-4.175599314	0.005	0.12	0
MAPK12	0	-3.92082517	0.006	0.138	0
GK-AS1	0	-2.506798089	0.008	0.05	0
AC090825.1	0	2.64337762	0.026	0.006	0
LINC01354	0	-3.617928982	0.004	0.058	0
CCDC163	0	-2.96205802	0.006	0.053	0
AC093110.1	0	-4.233020291	0.001	0.024	0

MYO16-AS1	0	-5.475208645	0.001	0.081	0
COLGALT2	0	-2.942800026	0.004	0.046	0
ARHGAP15	0	-3.087284512	0.01	0.05	0
AC092171.4	0	-3.808647055	0.002	0.036	0
ECSCR	0	2.933512151	0.021	0.006	0
TFR2	0	-3.049298932	0.004	0.098	0
TMEM71	0	2.953271031	0.056	0.01	0
UBXN10-AS1	0	4.108946471	0.013	0.001	0
ESCO2	0	-4.369174603	0.003	0.105	0
AGMO	0	-3.141464613	0.005	0.032	0
AC016134.1	0	-3.963380038	0.002	0.054	0
UCKL1-AS1	0	-2.546667333	0.006	0.061	0
SFTA1P	0	-3.659774806	0.005	0.071	0
IFIT1	0	-2.87729108	0.009	0.05	0
AMHR2	0	3.584833598	0.012	0	0
LINC02570	0	-4.019726109	0.002	0.05	0
ARL11	0	-3.586959603	0.002	0.071	0
AP005717.2	0	-3.117107648	0.004	0.051	0
FAM180A	0	-4.118442685	0.004	0.116	0
AL157395.1	0	3.411412179	0.017	0.004	0
SKA3	0	-3.397604607	0.009	0.138	0
CYP2A6	0	5.41594905	0.013	0	0
TBC1D24	0	-2.68565592	0.029	0.201	0
ZBTB47	0	2.540048921	0.026	0.013	0
CASP14	0	-8.973555234	0	0.054	0
SLC13A2	0	2.922550307	0.076	0.014	0
GHRHR	0	-3.139975441	0.001	0.026	0
TCF19	0	-2.95028174	0.014	0.14	0
HHIPL1	0	-2.812575026	0.005	0.051	0
TMEM171	0	-4.031806115	0.001	0.05	0
DLX6	0	-6.360446819	0	0.018	0
HOXC13-AS	0	-5.150403176	0.001	0.048	0
KIF14	0	-3.330201154	0.02	0.18	0
CPSF4L	0	-4.906667695	0.001	0.042	0
AL121987.1	0	-4.433393911	0.001	0.024	0
GLOD5	0	3.29721862	0.018	0.003	0
AC010422.2	0	-2.613206182	0.006	0.064	0
DTL	0	-3.780323333	0.008	0.138	0
ZBP1	0	-2.763398519	0.009	0.085	0

IGFALS	0	3.270767924	0.015	0.002	0
RAB23	0	-2.527913119	0.013	0.129	0
QRFPR	0	-6.600341988	0.001	0.154	0
AC087379.2	0	-7.080450753	0	0.027	0
POTEJ	0	5.237757193	0.013	0	0
SH3TC2	0	-2.93124416	0.006	0.077	0
MRLN	0	-3.634093038	0.002	0.055	0
AC016735.1	0	-3.661092404	0.002	0.058	0
CALY	0	4.369327946	0.012	0.001	0
TPM1-AS	0	3.342674436	0.039	0.007	0
SCAT1	0	-4.926693713	0	0.035	0
AP001372.2	0	-3.820138381	0.004	0.122	0
KIF11	0	-4.269049665	0.004	0.091	0
HAND2-AS1	0	-4.10031962	0.005	0.092	0
BEND6	0	-3.643288803	0.004	0.128	0
LIF	0	2.756337907	0.207	0.097	0
CXCL10	0	-4.106574514	0.01	0.113	0
RACGAP1	0	-2.593831641	0.024	0.174	0
BDKRB1	0	3.539259423	0.012	0.001	0
C17orf99	0	-4.688176505	0.001	0.053	0
AP000550.1	0	4.483507627	0.018	0.001	0
AL109615.3	0	-2.603686378	0.012	0.136	0
RNF183	0	2.962783772	0.048	0.016	0
AL365181.3	0	4.138184659	0.012	0.001	0
PGBD4	0	-2.881932578	0.006	0.069	0
AL645568.1	0	-3.157670389	0.006	0.079	0
CYP4F3	0	-6.588232046	0	0.013	0
TAT	0	7.184251391	0.023	0	0
AC092903.2	0	2.685966564	0.029	0.008	0
UBXN10	0	3.971564127	0.017	0.002	0
RLBP1	0	-3.599494019	0.002	0.028	0
MTFR2	0	-3.187922317	0.008	0.12	0
KCNJ8	0	5.035184155	0.018	0.001	0
MTRNR2L10	0	2.945358324	0.054	0.009	0
SMC1B	0	-3.131413597	0.012	0.145	0
MELK	0	-3.208662727	0.016	0.183	0
DOC2B	0	3.075149148	0.017	0.003	0
PCDHGA1	0	-4.582787794	0.001	0.032	0
AP001025.1	0	-3.587161164	0.004	0.085	0

EFNB3	0	2.572013092	0.017	0.001	0
AC002451.1	0	2.987655933	0.023	0.006	0
MMP9	0	-3.629471719	0.004	0.054	0
FAM24B	0	-3.348537593	0.02	0.167	0
TMPRSS6	0	4.110225738	0.013	0.001	0
SH3TC2-DT	0	-4.206655618	0.002	0.044	0
TICRR	0	-4.574350957	0.003	0.065	0
IZUMO4	0	2.801434964	0.034	0.013	0
BLM	0	-4.080541357	0.004	0.087	0
PCDHGA5	0	-5.628133366	0.001	0.034	0
FAM237B	0	6.77259621	0.02	0	0
FIGLA	0	-5.830960576	0	0.039	0
CPXM1	0	3.696059155	0.016	0.004	0
LINC01816	0	-3.056189462	0.008	0.116	0
AL158064.1	0	-3.173017203	0	0.018	0
HIST1H1B	0	-6.670696251	0.001	0.072	0
AC103740.2	0	3.028937621	0.014	0.002	0
AC080013.1	0	-2.919125075	0.006	0.067	0
LINC01684	0	-2.619628461	0.007	0.06	0
CPEB1	0	-3.246222722	0.004	0.081	0
E2F2	0	-5.510787567	0.001	0.071	0
OR4E2	0	-6.196890287	0	0.02	0
UQCRHL	0	-4.689282025	0.01	0.209	0
HACD4	0	-2.768327087	0.018	0.155	0
LRRN3	0	-2.571712662	0.003	0.036	0
SLC18A2	0	2.504117511	0.048	0.022	0
CHAD	0	2.671652786	0.034	0.011	0
AL391421.1	0	5.187809279	0.013	0	0
LYL1	0	-4.270746907	0.002	0.038	0
EPHB2	0	-2.64186983	0.024	0.22	0
AC069431.1	0	-4.20522715	0.001	0.029	0
AL451085.1	0	-3.792384173	0.002	0.029	0
IL12A	0	-3.109568011	0.003	0.03	0
AC104695.2	0	2.773590736	0.031	0.01	0
AC004540.2	0	-6.724637254	0	0.106	0
CEP55	0	-3.754749642	0.006	0.123	0
AC011483.2	0	-2.713886458	0.003	0.034	0
CYP27C1	0	-2.749202339	0.005	0.069	0
TMCO1-AS1	0	-3.593111143	0.002	0.046	0

ADRB1	0	-2.747824911	0.002	0.043	0
LINC02763	0	-3.003399944	0.004	0.081	0
LINC01894	0	-4.658924345	0.001	0.024	0
MAST1	0	-2.544210267	0.005	0.067	0
NCAPH	0	-3.900366436	0.005	0.113	0
RMST	0	3.751966612	0.027	0.005	0
PGR-AS1	0	2.790441136	0.017	0.004	0
AC008957.2	0	-2.795587976	0.003	0.029	0
DUOX2	0	-2.888799177	0.001	0.019	0
AC103876.1	0	2.805604026	0.03	0.009	0
IL2RG	0	-4.555526587	0.002	0.048	0
BEND5	0	3.450025802	0.016	0	0
AC007743.1	0	-5.404321351	0.001	0.085	0
CD207	0	-6.205873484	0	0.044	0
SLC29A4	0	-3.141294388	0.005	0.042	0.0000000001020799702
AC007036.2	0	3.009430161	0.017	0.006	0.0000000001037913814
TMTC1	0	2.705369472	0.036	0.013	0.0000000001285859551
DGKB	0	-3.036791946	0.003	0.035	0.0000000001308706255
AC021055.1	0	-5.67172616	0	0.017	0.0000000001358712377
AMIGO2	0	2.560679425	0.054	0.039	0.0000000001402861994
PCP4	0	-3.625220899	0.005	0.03	0.00000000015885942
RELL2	0	-2.545703912	0.01	0.121	0.0000000001874634444
GABRG3-AS1	0	-3.053982759	0.004	0.021	0.0000000002057181878
ART3	0	-3.910159467	0.002	0.066	0.0000000002200363508
PCDH19	0	-3.529196539	0.007	0.101	0.0000000002212344493
TRIL	0	-2.683153217	0.007	0.087	0.0000000002579545551
AC007529.1	0	-4.842873059	0.001	0.035	0.0000000002721654782

CORO1A	0	-2.911979671	0.008	0.081	0.0000000002798620583
C12orf81	0	4.859955449	0.013	0	0.0000000002808269047
AC080132.1	0	-2.604386954	0.008	0.069	0.000000000343958171
AC110285.1	0	-2.66160447	0.003	0.028	0.000000000429679327
IKZF2	0	-2.669138778	0.014	0.106	0.0000000004444522258
AL365181.2	0	3.127140771	0.012	0.004	0.0000000004677087564
TMEM229B	0	3.26160419	0.024	0.006	0.000000000470910684
PWWP3B	0	3.910533262	0.017	0.001	0.0000000004789672791
AL132708.1	0	5.536008387	0.013	0	0.0000000004834667745
FADS2	0	-3.750576192	0.032	0.317	0.0000000005574776973
ERP27	0	-2.632125228	0.012	0.133	0.0000000006047963625
MRAP2	0	-2.900311379	0.004	0.049	0.0000000006836198922
POGLUT2	0	-3.410465825	0.003	0.068	0.000000000692267174
HMGA2-AS1	0	2.978775189	0.021	0.005	0.0000000007698714689
CALHM3	0	-7.25143067	0	0.047	0.0000000008300488773
TLCD3B	0	2.692454335	0.014	0.006	0.000000000867645849
AC079226.2	0	5.101282161	0.011	0	0.0000000009126466481
LINC02175	0	-2.664491595	0.006	0.048	0.0000000009174899889
MYBL2	0	-3.063378539	0.014	0.193	0.0000000009305304108
SNTG2	0	2.652335731	0.028	0.009	0.0000000009517063672

AL033384.2	0	-5.619349156	0	0.032	0.0000000009924972276
EBI3	0	4.449449368	0.021	0.002	0.0000000009990398601
AL358075.2	0	-2.579538977	0.005	0.068	0.000000001042705775
PTGS1	0	-3.452579781	0.005	0.07	0.000000001119056453
SPDYC	0	-7.405365613	0	0.042	0.000000001126838575
AC009299.3	0	-3.270016264	0.003	0.041	0.000000001368537146
AC025811.1	0	4.971027801	0.013	0	0.000000001424493771
AFAP1L1	0	-3.468242192	0.005	0.084	0.000000001590575963
SPATC1	0	-2.511399568	0.005	0.034	0.000000001649132259
PCAT18	0	2.620082944	0.022	0.005	0.000000001955981128
AC138956.1	0	3.303288636	0.017	0.005	0.000000001996927523
MAGI2-AS3	0	2.657835947	0.021	0.003	0.000000002002216001
AL357153.1	0	3.717733529	0.02	0.004	0.000000002035106178
AC004990.1	0	-4.058790897	0.001	0.016	0.000000002050506993
LINC01915	0	-3.041850289	0.001	0.037	0.000000002405920279
AL390294.1	0	3.384693848	0.016	0.002	0.000000002623725124
KIF15	0	-3.770870508	0.006	0.126	0.000000003069916305
HIST1H2AG	0	-4.084512515	0.002	0.048	0.000000003088569784
AC018618.1	0	3.340840669	0.012	0.001	0.000000003226764413
SLC45A2	0	2.706920337	0.012	0.003	0.000000003269485117

PGPEP1L	0	-6.750381427	0	0.031	0.000000003505104637
LINC02845	0	-6.816301531	0	0.04	0.0000000040611769 4
DEPDC1B	0	-3.145712353	0.01	0.141	0.000000004866429903
MUC5AC	0	3.897098379	0.011	0	0.0000000056259791
FOXRED2	0	-2.504610689	0.017	0.159	0.000000005794928351
ZNF442	0	3.070473341	0.021	0.006	0.000000006080565091
AC107464.3	0	4.773919237	0.014	0	0.000000006995086565
AC025946.1	0	-3.657289525	0.002	0.027	0.000000007000037643
ZNF695	0	-3.074699005	0.013	0.136	0.000000007217103779
CNNM1	0	-2.97620871	0.003	0.04	0.000000007637392779
AC010280.1	0	-2.713068595	0.005	0.059	0.000000007880767969
SMAD6	0	-3.432610807	0.004	0.082	0.000000008951087159
LINC02657	0	-2.904507037	0.004	0.048	0.000000009041023676
IGHM	0	-2.712875134	0.032	0.048	0.000000009129205201
NTSR1	0	-5.992418011	0	0.014	0.000000009583205829
MSS51	0	2.971614077	0.013	0.004	0.0000000104088941 6
AC021733.2	0	-3.218042258	0.002	0.023	0.0000000105355353 3
AC090192.2	0	3.638289166	0.012	0.001	0.0000000112591397 8
KCNK3	0	-4.420218378	0.001	0.05	0.0000000116945025 7
NEIL3	0	-5.576632267	0.001	0.059	0.0000000122271202 6

EXO1	0	-3.725422282	0.006	0.11	0.00000001268594165
SLC6A7	0	2.592709375	0.022	0.004	0.00000001311999611
AC010967.1	0	-3.436420994	0.003	0.046	0.00000001343766409
FOXC2	0	3.981182887	0.043	0.004	0.00000001427661695
LINC02315	0	3.55739973	0.011	0.002	0.00000001437932326
DNAJB5	0	2.57380879	0.023	0.008	0.00000001525199116
Z84486.1	0	-2.649869481	0.003	0.028	0.00000001542206335
AC005487.1	0	-2.737094406	0.003	0.025	0.00000001573608917
PTN	0	3.512598811	0.094	0.015	0.00000001816149049
KIFC1	0	-3.609463766	0.01	0.176	0.00000002096106849
MROH9	0	-3.080457583	0.007	0.026	0.00000002121432187
S1PR1	0	3.015499859	0.011	0.003	0.00000002158328307
SPATA4	0	3.51768443	0.01	0.002	0.00000002168940206
LINC00499	0	2.651903097	0.022	0.009	0.00000002238919925
GPR55	0	-3.958213286	0.001	0.073	0.00000002345111756
AC002454.1	0	-2.609418201	0.006	0.068	0.00000002353975048
AC007132.1	0	-6.033168831	0	0.01	0.00000002372326875
ANGPT1	0	-3.729760103	0.002	0.052	0.0000000250013226
ZNF853	0	3.271319154	0.017	0.004	0.00000002668093891
KCNJ10	0	-5.000140825	0.001	0.012	0.00000002853933753
AZU1	0	2.65947225	0.023	0.009	0.0000000285418151

EBF3	0	-3.788867648	0.003	0.07	0.00000002909919483
MAGEB1	0	-6.449856462	0	0.023	0.00000002954864782
WNT16	0	-3.05491711	0.001	0.025	0.000000029551724 8
WNT5A-AS1	0	3.895181958	0.013	0.002	0.00000002961213623
AC010273.3	0	-2.571768256	0.003	0.057	0.00000003068011134
DDIT4L	0	-2.916818662	0.003	0.052	0.00000003483534545
CU638689.4	0	-3.381655159	0.004	0.075	0.00000003499760673
AC073842.2	0	-3.286705945	0.003	0.039	0.00000003852302023
PRSS51	0	2.508321533	0.012	0.003	0.00000004052036003
AC092811.1	0	-4.143859101	0.002	0.033	0.00000004325870217
ADARB2	0	-2.52887557	0.015	0.045	0.00000004697983885
MRPS30-DT	0	2.716315534	0.062	0.023	0.00000004999052275
AL355388.2	0	-3.584217474	0.001	0.017	0.00000005205476846
LINC02367	0	-4.751516002	0.001	0.027	0.00000005262051712
MEG8	0	2.596120226	0.019	0.009	0.00000005309506923
CFAP126	0	2.858823358	0.014	0.001	0.00000005466955461
CALHM6	0	-3.425954382	0.005	0.072	0.00000005524962011
AC108067.1	0	2.603200777	0.042	0.02	0.00000005744001066
LINC01644	0	5.377735591	0.01	0	0.00000006133883494
KCNJ3	0	3.738609171	0.014	0.001	0.00000006227454784

AC013470.2	0	-2.637695594	0.005	0.038	0.00000006286093118
AC005072.1	0	2.736954754	0.018	0.006	0.00000006424035285
LINC01234	0	-3.9195247	0.002	0.048	0.00000006511349454
LINC00111	0	-3.196323252	0.001	0.034	0.00000006521319142
RBM24	0	3.450185396	0.012	0.002	0.00000006572940187
FCGR3A	0	-4.881997379	0.001	0.02	0.00000006735801764
COL4A4	0	-3.099560887	0.007	0.096	0.00000007059982694
AL135936.1	0	-6.21775969	0	0.022	0.00000007418890003
PHYHIP	0	3.659307578	0.013	0.001	0.00000007809305974
AC011365.1	0	3.453948322	0.015	0.001	0.00000008213637875
LINC01723	0	-4.45711822	0.001	0.025	0.0000000857499295
MTRNR2L6	0	3.45532715	0.032	0.008	0.00000008960287634
KRT6C	0	5.295929459	0.04	0.002	0.00000009736935409
AL359513.1	0	-3.657811836	0.003	0.056	0.00000009844006813
IGFLR1	0	-2.723422299	0.015	0.144	0.0000001067856756
HOXC12	0	-6.549996238	0	0.023	0.0000001078497334
AC093838.1	0	4.906478778	0.01	0	0.0000001099045929
AC073648.7	0	3.923012027	0.015	0.001	0.0000001135323375
LINC01031	0	-2.74410727	0.008	0.093	0.0000001291722801
AC005618.1	0	-4.758944195	0	0.029	0.0000001300515886
AC008555.4	0	3.335824005	0.015	0.002	0.0000001396452295
AL512328.1	0	-3.10950052	0.003	0.042	0.000000144161068
AC009063.2	0	-4.289469892	0	0.027	0.0000001446998105
NOTUM	0	-4.016613337	0.001	0.019	0.0000001447998089
AC093382.1	0	2.727765841	0.014	0.004	0.0000001462828165
SHROOM4	0	2.667542143	0.027	0.01	0.0000001465048166
GJB6	0	-4.338002344	0.001	0.052	0.0000001528768326

HOTAIR	0	-3.642554054	0.003	0.087	0.0000001575655694
CCL26	0	-3.638648279	0.001	0.015	0.0000001586702372
CA6	0	3.141798631	0.019	0.004	0.0000001604991158
CD53	0	-2.620421343	0.006	0.033	0.0000001847181581
ALDH3B1	0	-2.564375063	0.011	0.1	0.0000002163756365
AC010148.1	0	2.55372814	0.011	0.003	0.0000002289403975
LINC01204	0	3.049425497	0.015	0.004	0.0000002487554653
PLAC8	0	-4.063339914	0.003	0.035	0.0000002530538713
WFDC6	0	-4.457277241	0	0.016	0.0000002586518258
CCNF	0	-2.684634355	0.015	0.133	0.0000002800529939
HDAC11-AS1	0	-3.081861912	0.004	0.038	0.00000028188097
AC008676.1	0	3.198107771	0.013	0.001	0.0000003055112838
CASC17	0	-6.004235155	0	0.021	0.0000003068035513
ALPP	0	-7.517883742	0	0.05	0.0000003196665685
MAGEB4	0	-6.543264922	0	0.022	0.0000003220075995
ZFX-AS1	0	3.707096956	0.016	0.003	0.0000003300155465
TESC	0	3.068733468	0.032	0.004	0.0000003411727447
HABP2	0	2.662729908	0.015	0.004	0.0000003411732953
LINC02198	0	-2.799182817	0.006	0.074	0.0000003490367576
ARMC3	0	2.634849335	0.01	0.004	0.0000003875920076
TLR7	0	-6.327503833	0	0.024	0.000000394410364
FDCSP	0	-2.88566809	0.046	0.181	0.0000004138782463
HAND2	0	-7.142702613	0	0.054	0.0000004216228618
PRIMA1	0	-2.996121333	0.002	0.065	0.000000437689903
FAM90A1	0	-3.328169973	0.003	0.056	0.0000004403030992
HMGCS2	0	-3.395951268	0.032	0.095	0.0000004490873004
PEAK3	0	-2.95509324	0.003	0.023	0.0000004648148414
AL390800.1	0	-4.801916687	0.001	0.015	0.0000004691773908
AC116049.2	0	-3.144834999	0.004	0.044	0.000000470119667
LINC02515	0	-2.690747511	0.009	0.051	0.0000004849124113
LINC01098	0	-5.971806358	0.001	0.03	0.000000489522831
DKK3	0	3.108072784	0.045	0.012	0.0000005165493412
BRINP3	0	-2.932689675	0.01	0.105	0.0000005724122901
AC008056.1	0	4.105759853	0.013	0.001	0.0000005872985673
FZD9	0	3.110959977	0.023	0.007	0.0000006332436154
IFIT2	0	-2.858884654	0.01	0.039	0.0000006680505695
AC096751.2	0	2.647278769	0.011	0.002	0.0000006722319702
IL1R2	0	-2.512758158	0.01	0.051	0.0000006924993104
AC092110.1	0	-4.647970499	0	0.014	0.0000007651541898

Z84484.1	0	-3.935964809	0.002	0.044	0.0000007657294486
FCRLB	0	-2.801408991	0.003	0.04	0.000000806707103
AC007221.1	0	-3.450875503	0.001	0.011	0.0000008490977528
AL157871.6	0	-3.739918703	0.002	0.023	0.0000009432871496
SH3BP1	0	-3.086737318	0.025	0.175	0.000001051327236
ROR1-AS1	0	-4.33492669	0.001	0.051	0.000001071229077
AC125793.1	0	-2.906466891	0	0.039	0.000001086426302
GPR162	0	4.806311347	0.012	0	0.000001091582663
XRCC2	0	-3.25886231	0.008	0.1	0.000001150167111
KIF18B	0	-4.592484219	0.003	0.072	0.000001158401227
KRT222	0	3.872688399	0.01	0.001	0.00000118433559
KLHDC8A	0	-3.98322442	0.001	0.021	0.000001191646641
P2RX5	0	-3.791661255	0.002	0.05	0.000001272039461
AL355303.1	0	-2.64065178	0.002	0.021	0.00000133253938
AF127936.2	0	3.562357386	0.01	0.002	0.000001351178602
NCF1	0	-2.668075347	0.005	0.037	0.000001372312641
ERG	0	3.117200672	0.044	0.012	0.000001404750292
LINC02473	0	-5.990047261	0	0.026	0.000001454295497
TRAIP	0	-4.121773982	0.003	0.087	0.000001466034994
AL606516.1	0	-3.329916314	0.001	0.024	0.000001657727625
ZNF300	0	2.754326112	0.014	0.004	0.000001695188085
LINC00239	0	-3.478978438	0.002	0.032	0.000001733604435
ORC1	0	-2.949953488	0.006	0.09	0.000001771042806
DLK1	0	-6.017411902	0.009	0.081	0.000001792680272
ZNF280B	0	-2.839247531	0.003	0.027	0.000002171367519
MSLNL	0	-6.580870857	0	0.024	0.000002431669552
AC021504.1	0	3.338666528	0.011	0.001	0.000002436436994
ADAMTS8	0	3.596051313	0.014	0.003	0.000002450048051
HOXA1	0	-3.694110887	0.001	0.03	0.000002453008722
SKA1	0	-4.759466267	0.002	0.076	0.000002551242333
HCAR1	0	3.158647764	0.021	0.004	0.000002693050434
LINC01995	0	-5.672834338	0	0.016	0.000002750310305
FAM72D	0	-2.776981136	0.001	0.039	0.000002769288967
AC113143.3	0	-2.56380906	0.004	0.049	0.000002810829083
LINC01387	0	-5.977811684	0	0.021	0.000002830194772
AC022201.1	0.0000000001008631564	-3.425259121	0	0.013	0.000003118587932
AL031731.1	0.0000000001034236693	-3.216550852	0.003	0.023	0.000003197756431

AP006248.4	0.0000000001061635143	3.209005846	0.012	0.001	0.000003282469699
POLQ	0.0000000001179181535	-4.220778625	0.005	0.109	0.000003645911387
AC008764.6	0.0000000001223557863	-2.658850448	0.004	0.038	0.000003783118555
AC023469.1	0.0000000001223817386	-2.994710203	0.004	0.02	0.000003783920976
KIF20A	0.0000000001253538252	-4.833278694	0.001	0.056	0.000003875814922
AC022167.4	0.0000000001290197037	-2.869479621	0.003	0.035	0.000003989160218
IGHG2	0.0000000001316825402	-8.925185118	0	0.015	0.00000407149246
C5orf66-AS1	0.0000000001318664767	-3.059357512	0.001	0.015	0.000004077179592
ZNF732	0.000000000142480833	-3.577442631	0.003	0.049	0.000004405364874
AL133243.2	0.0000000001435885836	-3.059730422	0.002	0.033	0.000004439615415
CT70	0.0000000001450634319	-2.763610736	0.001	0.021	0.000004485216252
AC016700.3	0.0000000001532395525	-2.809678689	0.004	0.022	0.000004738013725
NOX5	0.0000000001626053129	-3.1825276	0.004	0.07	0.000005027593669
AC007491.1	0.0000000001640569224	-6.128558065	0	0.019	0.000005072475985
PDYN-AS1	0.0000000001771071351	-3.619463338	0.001	0.012	0.000005475975509
MEG3	0.0000000001858446461	3.668792026	0.033	0.012	0.000005746130613
PCDHGA8	0.0000000001930595123	-4.12409588	0.001	0.033	0.000005969207061
AL136090.2	0.0000000002001134957	-3.584322845	0.001	0.028	0.000006187309173
BHLHE22	0.0000000002037813217	-3.06402843	0.001	0.016	0.000006300714687
CDH17	0.0000000002129293823	-4.627844416	0.001	0.015	0.000006583563571

LINC01994	0.0000000002306194712	-5.647204429	0	0.012	0.00000713052343
AC091057.6	0.0000000002385911818	-3.85135108	0.004	0.104	0.00000737700075
AP000553.2	0.0000000002445879087	-4.227015691	0.001	0.018	0.00000756241355
AC117386.2	0.0000000002452974085	-3.193205798	0.003	0.046	0.000007584350573
GLB1L3	0.0000000002517960066	-3.343359199	0.002	0.051	0.000007785280729
MMP11	0.0000000002581628083	-2.532284525	0.008	0.075	0.00000798213587
IGHV1-18	0.0000000002763245444	-6.246759913	0.001	0.034	0.000008543678588
HS6ST3	0.0000000002837588486	-3.182218811	0.015	0.129	0.00000877353984
PIEZO2	0.0000000002873203213	3.265872884	0.029	0.009	0.000008883657014
HS3ST2	0.000000000324067585	4.097055846	0.017	0.003	0.00001001984566
SELE	0.0000000003241554796	2.744416734	0.029	0.009	0.00001002256327
CECR2	0.0000000003362063068	-2.807481264	0.011	0.064	0.0000103951628
NMU	0.0000000003786099103	-4.177338553	0.003	0.066	0.00001170623982
DCLRE1A	0.0000000003924587586	-3.294180429	0.003	0.029	0.00001213443236
KLHL7-DT	0.0000000004171927773	-2.972613089	0.001	0.024	0.00001289918348
OVCH1	0.0000000004398295274	4.222013294	0.014	0.002	0.00001359908916
RIIAD1	0.0000000004737535824	3.079388922	0.015	0.003	0.00001464798701
AP000462.1	0.000000000474374155	3.99811122	0.026	0.002	0.0000146671745
H3F3C	0.0000000004752605886	-2.647247836	0.003	0.037	0.00001469458214
AL590999.1	0.0000000004771911585	2.50577344	0.019	0.005	0.00001475427343

AP000880.1	0.0000000004774451086	-3.838653272	0.001	0.038	0.00001476212531
FBN2	0.0000000005110037424	-2.572555145	0.007	0.077	0.00001579972471
AC245014.3	0.0000000005376770885	-2.511761722	0.005	0.04	0.0000166244379
SIX3-AS1	0.0000000005494209752	-3.168279687	0.001	0.015	0.00001698754713
LINC01722	0.0000000005515443882	-3.539280829	0.001	0.02	0.00001705320094
ITIH2	0.0000000005525569307	4.220181666	0.016	0.004	0.00001708450774
CDC25A	0.000000000597586462	-3.168429836	0.003	0.064	0.00001847677582
FFAR2	0.0000000006035677448	-3.818497417	0.001	0.016	0.0000186617111
SNTG2-AS1	0.0000000006572961753	4.463780587	0.011	0	0.00002032294044
SLC8A1	0.000000000730170844	-3.474576475	0.005	0.077	0.00002257615232
AL627422.2	0.0000000007536209433	-2.798251026	0.002	0.028	0.00002330120594
ATP2B2	0.000000000796465506	-3.83949816	0.001	0.029	0.00002462591698
FXYD1	0.000000000856110642	3.129572898	0.014	0.005	0.00002647008494
AC087623.1	0.0000000008892604073	5.404885421	0.01	0	0.00002749504253
SFTA2	0.0000000009351746412	3.520894219	0.019	0.006	0.00002891466473
AC012178.1	0.0000000009875039075	-2.52172599	0.002	0.021	0.00003053263331
AC092718.2	0.00000000101613038	-2.898521064	0.004	0.025	0.00003141773523
CR381653.1	0.000000001034168256	-5.150729953	0.002	0.05	0.00003197544829
AL136295.17	0.000000001068039368	-3.47262207	0.001	0.033	0.00003302270923
PLEKHD1	0.000000001096386271	-2.508358206	0.004	0.033	0.00003389916712

CPB1	0.000000001214705565	6.295344195	0.014	0.001	0.00003755748135
AL359878.2	0.000000001312524105	-2.509615789	0.001	0.011	0.00004058193282
SLC6A2	0.000000001369896164	-3.521970947	0.001	0.015	0.00004235581951
AC012494.1	0.000000001410528645	-3.70272414	0.004	0.041	0.00004361213516
CD37	0.000000001421264914	-2.819084967	0.005	0.036	0.00004394408987
AC016394.1	0.000000001431258353	-2.584998666	0.006	0.052	0.000044253077
UVRAG-DT	0.00000000143589741	-2.78405586	0.002	0.036	0.00004439651203
AC092807.3	0.000000001443335484	3.28656359	0.013	0.003	0.00004462648983
LINC01671	0.000000001486141335	-5.369663329	0	0.016	0.00004595000394
CFAP99	0.000000001555578258	2.826029016	0.011	0.001	0.00004809692415
LINC01393	0.000000001593631912	-3.417118925	0.002	0.031	0.00004927350509
CSRP3-AS1	0.000000001598472599	-2.611960539	0.003	0.03	0.00004942317427
HOXA7	0.000000001655791132	-2.913777813	0.025	0.109	0.00005119540602
AC105219.2	0.000000001718336164	-5.532065096	0	0.014	0.00005312923586
TLR8-AS1	0.00000000173517422	-6.557981595	0	0.023	0.00005364985171
AP003071.2	0.00000000179226413	-3.952336666	0.001	0.022	0.00005541501463
BIN1	0.000000001797772427	-3.107316484	0.05	0.327	0.00005558532568
ENTPD8	0.000000001810195483	-3.470584093	0.004	0.091	0.00005596943415
AC008517.1	0.00000000188523725	-6.500118054	0	0.035	0.00005828965052
SLC22A18AS	0.000000001889419798	-2.667409771	0.005	0.079	0.00005841897073

MCHR1	0.000000002221794615	3.925776698	0.012	0.001	0.0000686956677
AC098828.2	0.000000002329055693	-3.534999872	0.001	0.015	0.00007201207298
FABP4	0.000000002476478241	2.874952694	0.075	0.023	0.00007657023074
SUCLA2-AS1	0.000000002513776436	-4.805236329	0	0.019	0.00007772345363
AP003120.1	0.000000002573577476	-4.880062737	0.001	0.053	0.00007957244197
PRSS38	0.000000002620068704	-3.232949226	0.002	0.018	0.00008100990426
CIT	0.000000002784417932	-2.876875405	0.011	0.117	0.00008609141804
C10orf55	0.000000003052229485	3.29135909	0.011	0	0.00009437188344
GPR19	0.000000003274400835	-3.087910608	0.003	0.052	0.0001012411994
LINC01895	0.000000003278057366	-5.947830757	0	0.024	0.0001013542557
AL365258.1	0.000000003314591467	-4.726304388	0	0.012	0.0001024838536
COL4A3	0.000000003400195486	-2.505898356	0.008	0.1	0.0001051306442
CXCL11	0.000000003449846725	-4.553827229	0.002	0.029	0.0001066658109
AC073957.1	0.000000003705139317	-2.628155972	0.004	0.024	0.0001145592025
AC013391.2	0.000000003938585058	-3.694219798	0.001	0.014	0.0001217771114
LCTL	0.000000004225180629	-2.606770559	0.003	0.028	0.0001306383599
LVRN	0.000000004228008668	-3.028621034	0.002	0.027	0.0001307258
PANK1	0.000000004893980103	-3.101045669	0.004	0.068	0.0001513169708
AC008115.1	0.00000000499958373	-2.946089016	0.002	0.031	0.0001545821294
AC009242.1	0.000000005024252259	-3.138499853	0.002	0.027	0.0001553448556

AC073957.3	0.000000005138577829	-3.629499571	0.001	0.031	0.0001588796879
AL353150.1	0.000000005256223775	-3.559803189	0.001	0.034	0.0001625171829
SCGB1B2P	0.000000005552654205	-8.251203251	0.011	0.148	0.0001716825154
AC010974.2	0.000000005811462364	3.285365592	0.015	0.005	0.0001796846048
AC079772.1	0.000000005906121261	-2.51212909	0.002	0.018	0.0001826113633
DFFB	0.000000006102775709	-2.840386493	0.005	0.063	0.0001886917221
SH2D1B	0.000000006114283388	-2.614158698	0.003	0.013	0.0001890475281
AF233439.1	0.000000006700844889	2.542857553	0.034	0.011	0.0002071834231
NPB	0.000000006749369303	-2.822799637	0.01	0.079	0.0002086837495
AL356580.1	0.00000000688387279	-3.391542657	0.002	0.036	0.0002128424628
DGKI	0.000000007602752949	-2.577184574	0.011	0.092	0.0002350695184
AP005230.1	0.000000008075298368	-2.830368003	0.003	0.043	0.0002496801502
RAD54L	0.000000008140398265	-3.282409828	0.007	0.101	0.000251692974
AC099552.3	0.000000008210173069	-5.718180497	0	0.019	0.0002538503411
BLACAT1	0.000000008469578974	-4.759066496	0.001	0.026	0.0002618709123
AC084866.1	0.000000008865084818	5.514601595	0.012	0	0.0002740995575
PCDH8	0.00000000898342036	-3.030715735	0.001	0.019	0.0002777583741
LINC01708	0.000000009151024311	-3.320736774	0.001	0.013	0.0002829405207
LEFTY1	0.00000000974940 62	-3.449660525	0.001	0.03	0.0003014418903
PRR15L	0.00000001013771518	-2.514567238	0.019	0.128	0.0003134480156

SLURP1	0.00000001053948087	-3.158772426	0.007	0.058	0.000325870209
PWP2	0.00000001057182742	5.18883323	0.026	0.001	0.0003268703319
LINC02026	0.00000001066309665	-3.585448759	0.001	0.03	0.0003296922854
CD52	0.00000001138139223	-3.643702675	0.004	0.045	0.0003519012665
FOXJ1	0.00000001147460354	-2.738774431	0.003	0.043	0.0003547832668
AC007381.1	0.0000000117197934	-4.591483787	0	0.011	0.000362364292
AL032819.1	0.0000000121105692	-5.455449856	0	0.017	0.0003744466892
SPNS3	0.00000001215063999	-2.545195906	0.005	0.055	0.0003756856377
PLS3-AS1	0.00000001235798433	-5.020640619	0.001	0.033	0.0003820965175
PON1	0.00000001239407093	-2.711726322	0.002	0.021	0.0003832122791
AC007370.2	0.00000001384139224	-4.928438533	0	0.011	0.0004279620067
AC244102.3	0.00000001445827538	-5.146109867	0	0.01	0.0004470354165
NKG7	0.00000001450980605	-5.203339488	0.002	0.033	0.0004486286931
LPO	0.00000001511438376	-3.148593521	0.001	0.019	0.0004673216314
ONECUT2	0.00000001515530306	-4.074928717	0.001	0.034	0.0004685868153
AC107029.2	0.00000001576191246	-6.282158453	0	0.017	0.0004873425714
AC093627.3	0.00000001590462213	-2.76394228	0.002	0.028	0.0004917550115
LINC02028	0.00000001689887868	-2.686310675	0.004	0.052	0.0005224964301
AL023755.1	0.00000001753230987	-2.625049315	0.002	0.016	0.0005420814889
LMO3	0.00000001833104952	-2.751222502	0.004	0.024	0.0005667777201

OXCT1-AS1	0.0000000183709802	-4.35571363	0	0.01	0.0005680123369
DLEU7	0.00000001913676103	-2.958906406	0.003	0.023	0.0005916895142
AC104964.1	0.00000002040022838	2.572217043	0.011	0.004	0.0006307546613
AP000820.2	0.00000002097346957	-3.27641914	0.001	0.025	0.0006484787058
AC138150.2	0.00000002175391497	2.530231578	0.011	0.005	0.0006726092969
RIBC2	0.00000002200414638	-2.52911572	0.003	0.047	0.0006803462018
HOXA10-AS	0.00000002595940644	-3.529819843	0.01	0.082	0.0008026388878
LINC01358	0.00000002634402241	-2.854759417	0.003	0.032	0.0008145308288
AC024361.2	0.00000002709554384	-3.765902337	0	0.016	0.0008377671201
ARHGEF39	0.00000002732926224	-3.888411167	0.003	0.076	0.0008449934591
AL590006.1	0.00000002736141666	-2.64651929	0.003	0.028	0.0008459876417
TRPM8	0.00000002748042408	-3.646657601	0.002	0.045	0.0008496672321
PIMREG	0.00000002814474976	-3.830345637	0.005	0.126	0.0008702075179
AL354733.3	0.00000002897529489	-3.028148671	0.002	0.015	0.0008958871427
AC040173.1	0.00000002905245539	-2.841005155	0.004	0.04	0.0008982728682
AC018553.2	0.00000002950648704	-4.292866931	0.001	0.017	0.0009123110727
Z95114.4	0.00000003048136659	-3.790630614	0	0.01	0.0009424533735
NWD2	0.00000003092425317	3.130653328	0.01	0.003	0.0009561469838
TEKT4	0.00000003217152059	2.562479064	0.017	0.007	0.0009947112452
LINC01351	0.00000003601454626	-6.203192358	0	0.021	0.001113533756

AC137770.1	0.00000003674476454	-2.767852614	0.003	0.035	0.001136111375
AP002761.3	0.0000000371987564	-3.206170474	0.002	0.024	0.001150148349
MMP10	0.00000003984871297	3.302869776	0.016	0.004	0.001232082356
AC079310.1	0.000000042239988	-4.418403667	0	0.01	0.001306018189
C1QA	0.00000004388772143	-2.724398424	0.008	0.048	0.001356964459
AL096865.1	0.00000004405149101	-3.807435039	0.001	0.013	0.001362028051
PKIA-AS1	0.00000004499094488	-2.76535164	0.003	0.027	0.001391075025
AC007179.1	0.00000004659658673	-3.937313332	0.001	0.011	0.001440719865
MCOLN2	0.00000005588658718	-4.666829605	0.001	0.056	0.001727957389
DOCK4-AS1	0.00000005783451712	-5.487795168	0	0.01	0.001788185435
CLPSL1	0.00000005822873535	-3.63544431	0.023	0.22	0.001800374268
CCDC168	0.00000005863912095	-3.755884384	0.001	0.044	0.001813062981
PAX8	0.00000005951216615	2.543128239	0.029	0.012	0.001840056665
LINC00858	0.00000005995088278	-4.996576083	0	0.023	0.001853621345
AC079148.3	0.00000006363953809	-5.029727769	0	0.021	0.001967670878
C1QB	0.00000006483866281	-3.114005038	0.008	0.046	0.002004746616
ANXA2R	0.00000006501020164	-2.73356625	0.002	0.029	0.002010050425
LINC00514	0.00000006800373571	-4.205855046	0.001	0.029	0.002102607504
AL354822.1	0.00000006909869986	-2.55637658	0.005	0.051	0.002136462701
AC244453.1	0.00000006977213149	-4.482440316	0	0.01	0.002157284534

AL033528.2	0.00000007014573004	-3.462907321	0.001	0.034	0.002168835827
RCN3	0.00000007062532403	-2.769473746	0.011	0.095	0.002183664394
AC087664.1	0.00000007328711058	-6.536645153	0	0.018	0.002265964172
AC138474.1	0.00000007389084246	-2.806085871	0.004	0.049	0.002284630958
GIMAP6	0.00000007451434589	2.624202498	0.011	0.002	0.002303909061
AC099506.1	0.00000007575586313	-5.941039818	0	0.024	0.002342295532
AC004949.1	0.00000007840972202	-3.179649091	0.002	0.013	0.002424350195
IL6R-AS1	0.00000008025149149	3.560698757	0.013	0.003	0.002481295865
RAD54B	0.00000008106273011	-2.913416024	0.007	0.101	0.002506378552
CRYBB3	0.00000008119834772	-2.808829967	0.003	0.044	0.002510571713
SPOCK2	0.00000008832402908	-5.771592166	0.004	0.138	0.002730890655
AC007432.1	0.00000009071323376	-5.30166726	0	0.011	0.002804762475
AP000320.1	0.00000009175770709	-2.612113853	0.003	0.038	0.002837056545
AC104472.4	0.00000009274522983	-2.841868	0.003	0.033	0.002867589761
PRDM16	0.00000009610195747	3.52432835	0.011	0.001	0.002971376423
PCOLCE2	0.00000009946742534	-2.660143931	0.008	0.081	0.003075433324
AL009178.3	0.0000001026466819	-3.463988729	0.001	0.025	0.003173732757
VNN1	0.0000001055196725	2.586406285	0.053	0.025	0.003262562755
AC110285.3	0.0000001101727067	-4.69318164	0	0.01	0.003406429917
HIST1H3H	0.0000001163873279	-2.596681802	0.009	0.055	0.00359857979

LINC01224	0.0000001187590176	-5.508152532	0	0.064	0.003671910064
LINC01140	0.0000001193745148	-3.05103085	0.003	0.038	0.003690940624
GPR158	0.0000001228294671	-2.584753592	0.005	0.038	0.003797764293
GREM1	0.0000001325504494	-3.095926963	0.003	0.017	0.004098327344
PCDHGA6	0.0000001367481257	-3.558335012	0.001	0.021	0.004228115297
LINC01730	0.0000001389710268	-2.73951787	0.003	0.035	0.004296845178
LINC01338	0.0000001405327075	-5.284362435	0	0.015	0.004345130782
HLX	0.0000001440392845	2.668044377	0.011	0.004	0.004453550638
DOK7	0.0000001455955755	-3.674098647	0.003	0.092	0.0045016696
AC005144.1	0.0000001459234623	-2.665305432	0.002	0.017	0.004511807531
AC010883.3	0.000000152175972	-3.090345505	0.001	0.011	0.00470512888
ADGRG3	0.0000001552309018	-3.210629413	0.002	0.017	0.004799584253
FAM83A	0.0000001552679914	-3.093254736	0.016	0.197	0.004800731026
SCML4	0.0000001726872184	-4.686186001	0	0.011	0.005339316106
IGFL1	0.0000001727947581	4.631700559	0.012	0.002	0.005342641125
MSR1	0.0000001733310809	-2.774398961	0.002	0.016	0.00535922369
LINC02789	0.0000001734297956	-3.98658628	0.001	0.019	0.005362275852
GET4	0.0000001742515529	-5.233766544	0.003	0.087	0.005387683764
CNN1	0.0000001766975582	2.745412856	0.034	0.009	0.005463311803
AL161945.1	0.0000001832720371	-2.627848305	0.001	0.041	0.005666588114

SGCG	0.0000001905008359	-2.879170266	0.002	0.024	0.005890095344
MIR137HG	0.0000001914613601	-4.666913411	0.001	0.034	0.005919793791
AC090360.1	0.0000002004433577	-3.006835824	0.002	0.022	0.006197508176
AP001021.1	0.0000002025744597	-5.231300429	0	0.015	0.006263399719
TM4SF19	0.0000002373009288	-3.238772193	0.001	0.011	0.007337107416
AC108925.1	0.0000002735460325	-2.891553774	0.002	0.017	0.008457769779
SELL	0.0000002822427793	-4.39191617	0.001	0.017	0.008726664493
CBX2	0.0000002869234647	-4.871421522	0.002	0.067	0.008871386605
AL158817.1	0.0000002871278507	-5.151256072	0	0.014	0.008877706016
C1QC	0.0000003190054024	-3.520905923	0.004	0.038	0.009863328036
AC089985.1	0.0000003396995168	2.720829903	0.013	0.004	0.01050316936
AC022167.3	0.0000003652372786	-2.797115165	0.002	0.015	0.01129277142
PCDHGC3	0.0000003774710083	-2.963445825	0.001	0.013	0.01167102611
AC019068.1	0.0000003798511104	-2.944567973	0.002	0.02	0.01174461648
AC126544.2	0.0000003819032751	-2.833532837	0.001	0.01	0.01180806736
AP005436.1	0.0000003959339518	-3.60688512	0.003	0.09	0.01224188186
AP003119.2	0.0000004062355984	-4.57876206	0	0.03	0.01256039847
GZMB	0.0000004213189513	-2.652055841	0.003	0.026	0.01302676066
KLRG1	0.0000004262061244	-3.340748165	0.002	0.039	0.01317786716
AC106900.1	0.0000004278983896	-3.082143275	0	0.014	0.01323019031

AC012676.1	0.000000448456569	-3.473813931	0.001	0.026	0.01386582866
AL355870.2	0.0000004542049898	-3.923092004	0	0.011	0.01404356408
CKB	0.0000004561303165	-2.512517317	0.202	0.399	0.01410309325
TMED6	0.0000004636579898	3.009026017	0.02	0.006	0.01433584139
CENPI	0.0000004713184651	-3.729933437	0.003	0.065	0.01457269562
CDKN2A	0.0000004820491982	-4.217771252	0.055	0.457	0.01490447916
LINC01917	0.0000004866445189	-3.309478204	0.001	0.015	0.01504656188
AL137003.2	0.0000004876916899	3.155807996	0.01	0.001	0.01507893936
ZIK1	0.0000005184559322	3.280722337	0.01	0.002	0.01603013897
AC005899.6	0.0000005335861226	3.283629315	0.016	0.002	0.01649794932
LRFN5	0.0000005533325987	3.017490279	0.01	0.004	0.01710849062
KBTBD12	0.00000057035324	-3.447648075	0.002	0.06	0.01763475183
AC018541.1	0.0000005854719167	-3.013533284	0.001	0.013	0.01810220619
TNKS2-AS1	0.0000005918030181	-2.687350972	0.004	0.03	0.01829795752
AC126178.1	0.0000005919687091	-4.404521442	0	0.01	0.01830308052
FAM25A	0.0000005957223892	3.206207519	0.01	0.002	0.01841914055

Supplementary Table S7. List of DEG between epithelial cells with very low and extreme CIN by patient

Gene	p_val	p_val_adj	baseMean	log2FoldChange	lfcSE	stat
RNF223	0.0003401677622	0.01011646848	94.51416069	-3.774421488	1.053537217	-3.582618085
CAMK2N1	0.00005234435627	0.002421974821	148.5571921	-4.105548979	1.014992695	-4.044904953
WNT4	0.0001630976438	0.00596889872	60.58636484	-4.492999648	1.191704622	-3.770229272
AL445248.1	0.0003385922724	0.01008951439	36.74767637	-2.906246994	0.8109332332	-3.583830179
CLSPN	0.0001303179413	0.00496195434	45.80754201	3.256872646	0.8512792481	3.825856971
CSF3R	0.000001391375387	0.0001165508782	53.13782841	-4.296745017	0.8902890185	-4.826236118
GRIK3	0.000002522500818	0.0001950475248	15.64754034	-5.498189615	1.16826098	-4.70630254
ZC3H12A	0.0001648982813	0.006005643202	330.2583792	-2.547991409	0.6763103749	-3.767488278
DNALI1	0.0000000001274790389	0.00000005339247079	61.62498185	-5.428339811	0.8442012663	-6.430148862
AL139220.2	0.0005102667235	0.01378817501	43.14740382	2.614168158	0.7522118908	3.475308208
KIF2C	0.0001783864145	0.00635865333	33.07817331	3.530392517	0.9419885207	3.747808429
TSPAN1	0.00009082460077	0.003731480464	97.5638653	-3.079231225	0.786746359	-3.91388049
BEND5	0.0005545831199	0.0146188886	9.392479543	-5.229761627	1.514597354	-3.452905562

WLS	0	0.0000000002243703185	177.390955	-4.796243988	0.6574274677	-7.295472465
DEPDC1	0.0000005777794099	0.00005966957495	37.34040753	5.417331619	1.083791414	4.99850022
PTGER3	0.000001223640113	0.0001054288321	37.38178829	-5.204445651	1.072689933	-4.85177076
AK5	0	0	325.7941489	-5.548868547	0.6840682726	-8.111571271
ARHGAP29-AS1	0.0000000002641161324	0.0000001021113601	62.21285451	-6.29661533	0.9965365831	-6.318498925
F3	0.0000000003402439386	0.0000001251267831	139.5727183	-4.72122254	0.7518786711	-6.279234565
HENMT1	0.0006164764493	0.01586217048	69.5297396	2.630422948	0.768172705	3.424259845
SLC16A4	0.0003839503062	0.01113055477	47.17651124	-2.678121872	0.7542142772	-3.550876658
CHI3L2	0.0000004780773468	0.00005223514663	317.8419803	-4.226983616	0.8395353534	-5.034908416
PIFO	0.00008006213598	0.003413089636	29.96932843	-3.584827461	0.9088825938	-3.944214011
ITGA10	0.0008212491493	0.01934811668	23.43680331	-2.941244706	0.8791560814	-3.345531889
AC239799.2	0.0001425900246	0.005334919083	31.76136856	3.412554804	0.8971832471	3.803631883
ANXA9	0.0002494494972	0.008158784206	21.91716843	-3.737076462	1.020271791	-3.662824451
EFNA3	0.00004949439342	0.002346781333	90.91639017	3.113949734	0.767360369	4.058001767
LINC01133	0	0	93.96904608	6.987430601	0.9159773569	7.628387916

USF1	0.0008046600577	0.01901708155	48.86471855	3.44255802	1.027265334	3.351186792
NUF2	0.000000260592581	0.00003083591982	58.29373027	3.981272994	0.7730741851	5.149923605
C1orf105	0.00007720611784	0.00331656366	9.959662109	7.233358537	1.829881397	3.952911129
IER5	0.000000003500293313	0.0000008796237095	591.9324861	-4.222991372	0.7150064862	-5.90622806
ASPM	0.0000002015194573	0.00002490582277	45.51030353	4.946935564	0.9517125286	5.197930484
UBE2T	0.000001105021178	0.00009575580072	143.6367365	3.505484683	0.7195239396	4.871950036
BTG2	0.00000004176985467	0.000006920943613	996.3365583	-3.973858295	0.7247333212	-5.483200756
PIGR	0	0	615.5153465	-7.706370485	0.5917740013	-13.0224891
CENPF	0.0000000005607085334	0.0000002012943635	210.7855989	4.72951906	0.7626906779	6.201097243
TTC13	0.0001152955164	0.004492056319	59.34744918	3.426947025	0.8887496726	3.855919311
GNG4	0.0001088032257	0.004317197466	94.94338186	3.945708273	1.019543899	3.870071977
ERO1B	0.0002130827025	0.007285399067	192.9392621	-2.969576129	0.8019425164	-3.702978791
AL390860.1	0.0000005390942127	0.00005693354872	18.53258115	-4.91279226	0.9802354341	-5.011849285
GREB1	0.0000002531363937	0.00003078056891	78.3913493	-4.224331442	0.8194048056	-5.155365715
AC104695.2	0.0001116023868	0.004382137468	28.00099356	-4.407442238	1.140679327	-3.863874914
CYP1B1	0.0001984572366	0.00687893842	134.6592513	-3.261211011	0.8764405591	-3.720972264

NRXN1	0.00001800520446	0.001012023606	120.0918826	-4.153752488	0.9686262473	-4.28829231
AC007402.1	0.0000000007847288447	0.0000002544787238	88.92968811	-6.428009853	1.045547224	-6.147986149
OTX1	0.0004367215454	0.01217169586	10.02109751	5.87385774	1.670210305	3.516837204
AC008268.1	0.000002140640548	0.0001689873203	18.72171854	-6.778771261	1.430219373	-4.73967238
FHL2	0.000007634295645	0.000496163404	932.2742867	-3.57015071	0.7977649522	-4.47519122
BIN1	0.0000004077822103	0.00004487985523	171.0508105	4.218005985	0.8327276817	5.065288542
NCKAP5	0.000004522582466	0.0003157013816	217.2415541	-3.265358937	0.712059226	-4.585796824
LYPD6B	0.00008580591498	0.003573982282	164.4616476	-2.871464216	0.731103206	-3.927577109
LYPD6	0.000003864612666	0.0002828671348	59.46579402	-4.372192389	0.9466624464	-4.618533676
LINC01876	0.00000005391673949	0.000008468297897	74.82050529	-5.682488603	1.044981376	-5.43788505
CSRNP3	0.0002922838806	0.009068017185	46.05554431	-3.943137187	1.088649584	-3.622044451
LRP2	0	0	210.4140462	-8.233996047	1.015436663	-8.108822882
CDCA7	0.000003021972208	0.0002301277624	40.20571086	4.439179139	0.950711969	4.669320765
ZNF385B	0.0007843839264	0.01873409991	45.17285258	-3.498030257	1.041623771	-3.358247339

ITGAV	0.00008435921173	0.003543086893	386.9080526	-2.503031662	0.6366338483	-3.93166601
AC017101.1	0.0002195414001	0.007455507277	70.22005993	-3.22677018	0.8731857737	-3.695399396
TFPI	0.0005075811007	0.01374022951	151.7965908	-3.757481771	1.080753715	-3.476723436
MREG	0.000007226391046	0.0004737370617	163.9738012	-2.982896684	0.6647999018	-4.486909032
IGFBP5	0.0007852467931	0.01873409991	394.113376	3.15009582	0.9381027041	3.357943439
KCNE4	0.00002650874143	0.00136638828	28.91371518	-4.795376636	1.141333681	-4.20155535
SNORC	0.0008275446184	0.01943569744	171.1329816	-2.978334071	0.8908063541	-3.34341359
HJURP	0.00004943924506	0.002346781333	17.05490808	5.045724299	1.24332142	4.058262183
HES6	0.000008065571552	0.0005153079994	90.03648776	3.896687073	0.8730240817	4.463435951
CNTN4	0.0001094006902	0.004329510777	75.46239294	-3.421158147	0.8843088558	-3.868736725
ITPR1	0.0002206673224	0.007476903117	265.8712167	-2.666192401	0.7217435115	-3.694099577
EGOT	0.0007770899699	0.01862792141	119.9553552	-3.259153946	0.9697471104	-3.360828727
IL17RE	0.0004416906434	0.01226484626	43.99807316	2.599755351	0.739863123	3.513832857
SGO1	0.0000003714362743	0.00004118026577	49.89447763	4.350822652	0.8559484859	5.083042641
NEK10	0.0002239874145	0.007555441244	126.7301741	-2.840694207	0.7697726951	-3.690302637

CSRNP1	0.000000006369904255	0.000001433513677	215.9577634	-3.374971131	0.581215662	-5.806744985
SEMA3B	0.0000000001173207829	0.00000005054179328	81.6427026	-4.647152898	0.7212985578	-6.44275917
PTPRG	0.0004329005451	0.01210997109	172.8755751	-2.788420376	0.7923518957	-3.519169187
ADAMTS9-AS2	0.00002642658201	0.00136638828	165.0036386	-4.23596361	1.008020859	-4.202257894
COL8A1	0.00000005624005681	0.000008652934454	98.40899413	5.570195982	1.025750519	5.430361357
PHLDB2	0.000001377404829	0.0001160251956	248.8531468	-3.630812822	0.7519940771	-4.828246568
C3orf52	0.00000006908555132	0.00001001607637	205.5524502	-3.660353173	0.6786559076	-5.393533206
LSAMP	0.0001620881767	0.005960891532	92.9544805	-3.298225782	0.8744484515	-3.771778401
AC092691.1	0	0	82.72753033	-7.482083517	0.975047267	-7.673559806
MECOM	0.0000170153836	0.0009718104315	115.7559001	2.849798532	0.6626147949	4.300837461
TNIK	0.0000009586685025	0.00008707713061	228.2969976	-3.227923958	0.6587683124	-4.899938108
ALG3	0.0002733060815	0.008730739613	333.4256222	2.894849085	0.7954259238	3.6393698
EEF1AKMT4	0.000553046884	0.01460392455	132.7331679	2.602051129	0.7534197963	3.453653782
PLAAT1	0.0000008949586524	0.000084338666	77.91677843	4.176472675	0.8500113222	4.913431817
HES1	0.000008340624696	0.0005278166212	1367.992639	-2.969184028	0.6662967425	-4.456248753

MUC4	0.0000003010846372	0.00003439207697	40.4778852	-5.654622535	1.10382197	-5.122766796
TACC3	0.0004431339199	0.01228230376	27.25980647	3.257758404	0.9273526303	3.512966155
STK32B	0.000000004930423933	0.000001180014795	33.77920682	-5.550067143	0.948809947	-5.849503539
NCAPG	0.00001002656074	0.0006195921431	26.95763376	4.185111433	0.9475867408	4.416599824
LINC02438	0.000001988040281	0.0001594450604	15.34897503	6.65692395	1.40009111	4.754636253
AC024230.1	0.000001356254021	0.0001148853828	199.4370883	4.626999954	0.9577076983	4.831327933
RELL1	0.00001254174182	0.0007445054455	146.8015083	-3.03984689	0.6959433939	-4.367951354
TBC1D1	0.00009734424843	0.003956217191	81.25381031	-2.639134463	0.6772016018	-3.897117869
TMEM156	0.0003213871795	0.009711174135	25.10272649	-3.53732106	0.9832934082	-3.597421716
KLHL5	0.000003700902689	0.0002748877377	174.3953432	-3.420059499	0.7390713981	-4.627508936
AC079921.1	0.0000427474896	0.002085911483	31.12484151	-3.835457714	0.9372823184	-4.092105056
UGDH	0.0000000007267140568	0.0000002490316943	724.3122367	-3.26613366	0.5302027174	-6.160160167
AC021148.2	0.0002730102259	0.008730739613	16.21032083	-4.8445745	1.331055515	-3.639648719
RHOH	0.000004075339718	0.0002940094367	65.78410576	-3.215660328	0.6979182777	-4.607502671
UCHL1	0	0.000000002434330656	138.3731542	7.507978038	1.080194194	6.950581738

NPFFR2	0.00000006218788527	0.000009376689341	29.24566492	-6.756971544	1.248425803	-5.412393376
ALB	0.000000672000973	0.0000666607281	92.57566024	-5.874062053	1.182073795	-4.969285402
RASSF6	0.0001696394146	0.006133868329	80.40839214	-2.736549487	0.7277275932	-3.760403636
CXCL8	0.00000003860812049	0.000006615150462	833.2601242	-5.373104138	0.9774428699	-5.497103006
CXCL3	0.000000003615268886	0.0000008936233486	572.5175411	-5.07454346	0.8599611241	-5.900898678
CXCL2	0	0	2105.95204	-5.552941578	0.7158646307	-7.756971555
MTHFD2L	0.00005594731891	0.002556283862	570.996339	-3.385978849	0.8403428478	-4.029282641
EREG	0.000002082385117	0.0001661280571	130.6947566	-5.097840083	1.074301493	-4.745260167
AREG	0	0	4513.393392	-5.936591797	0.7830252172	-7.581609974
PARM1	0.00000002180290299	0.000004058570016	83.65923373	-5.294512656	0.9459468907	-5.597050647
CXCL10	0.000001033880113	0.0000922416825	183.9359938	5.498566289	1.125584175	4.88507782
TMEM150C	0.0001501608085	0.005576661752	39.57598315	-3.311663129	0.8736045686	-3.790803355
AC093866.1	0.0002418104289	0.008013225598	18.77297389	-4.558445935	1.241819081	-3.670781037

STPG2	0.0000141227518	0.000831808014	40.55066255	-3.948193448	0.9093146815	-4.341944025
NFKB1	0.000006485806694	0.000430806138	471.7754008	-2.622761075	0.5815563178	-4.509900409
CCNA2	0.0001115491487	0.004382137468	27.05758038	4.081837139	1.056378421	3.863991404
TBC1D9	0.000008141226995	0.0005179469225	308.4316409	-3.15693726	0.70760564	-4.461435977
INPP4B	0.00000002410928186	0.000004433167705	787.0785041	-4.652767511	0.8338912906	-5.579585209
FNIP2	0.0007251445553	0.01772079353	100.4385818	-2.647609005	0.7833414242	-3.379891479
HAND2	0.000226103654	0.007609801103	12.2586203	7.021637396	1.903961196	3.687909928
TENM3	0.0000008684949354	0.00008235953859	66.08793263	-5.317251119	1.080893521	-4.919310751
CTNND2	0	0	247.6782705	-6.370893561	0.7771524681	-8.197739597
DNAH5	0.0000001587660339	0.00002098934095	114.856971	-4.602703262	0.8780279177	-5.242092158
AC016576.1	0.0006112711803	0.01575512283	10.77888294	-5.504043328	1.606286761	-3.42656334
TRIO	0.00002939899828	0.001502637614	1486.842561	-3.259433442	0.7801285069	-4.178072475
OTULINL	0.0001533116438	0.005679687877	33.76259	-3.316015783	0.8759451927	-3.785642995
LIFR	0.00007223396253	0.003159892263	40.53451625	-3.167265455	0.7980400801	-3.968804993

CCL28	0.00008547700674	0.003570144897	363.7813838	-2.758962028	0.7022938395	-3.928500968
MRPS30-DT	0.0000000003039884846	0.0000001145884593	247.8216758	-6.514001717	1.034505349	-6.296730823
IL6ST	0.00000321223472	0.000239772649	525.7492874	-3.223564108	0.6922331481	-4.656760684
PLK2	0.0002650905127	0.008558960923	348.1511513	-2.68768297	0.7369122883	-3.647222352
ZSWIM6	0.00001212746293	0.0007256265321	1095.00833	-3.34296759	0.7640573268	-4.37528373
MAST4	0	0.000000002745239591	578.2853248	-4.216212431	0.6098663747	-6.913338078
NAIP	0.00008796109686	0.0036536568	26.65228831	-3.425283881	0.8734392848	-3.921605017
ENC1	0.0006333163473	0.01613031062	45.2564245	2.812854115	0.8232110035	3.416929685
FAM81B	0.0001563981716	0.005779832428	10.71752646	-4.89602126	1.295009434	-3.780683857
ELL2	0.00002442677848	0.001287786594	586.3742803	-2.572033431	0.6094819921	-4.22003187
TNFAIP8	0.000000006791262259	0.000001505862534	515.680879	-3.463148968	0.5975060507	-5.796006524
FNIP1	0.0007610531347	0.01836402165	405.132477	-2.53357245	0.752564681	-3.366584313
EGR1	0.0005009479004	0.01363410188	1169.45574	-2.615217182	0.7514454148	-3.480249037
RELL2	0.0007902960009	0.0187654852	27.55251724	2.906154175	0.8659135394	3.356171307

ABLIM3	0.00009118847964	0.003736249718	49.9592979	-3.941869078	1.00739951	-3.912915422
FABP6	0.0002909457091	0.009049825807	20.50257092	5.023215971	1.38639129	3.623231051
UBTD2	0.00006110823866	0.002758652762	126.0591	-3.005006392	0.7496604528	-4.008489951
AC022217.3	0.0006254496367	0.01601108595	109.7937217	-3.108534161	0.9088401331	-3.420331087
STC2	0.0000000001011425444	0.00000004485374368	647.9318517	-4.687278327	0.7249975376	-6.465233445
AC008663.1	0.000001959914235	0.0001580298761	14.21490191	-6.429777642	1.351499397	-4.757514252
SCGB3A1	0.0000002407273254	0.00002950964726	1813.91886	-7.672039753	1.485454567	-5.16477577
TUBB2B	0.0007624272155	0.01836402165	25.78093388	-4.413194281	1.311075615	-3.366086769
ATXN1	0.0000001877584022	0.00002387776062	591.6353657	-3.388416944	0.6502348643	-5.211066231
ID4	0.0003702385084	0.01083972084	160.1878681	-3.512844728	0.9866330989	-3.560436734
DCDC2	0.0000002002395648	0.00002490582277	29.83650503	-4.956902944	0.9534128209	-5.199115048
HIST1H1A	0.0000001064231563	0.00001445629145	30.39904554	6.432248612	1.210115579	5.315400217
HIST1H1E	0.00003439812717	0.001734631978	40.14358294	3.930538079	0.9488997278	4.142205929
AL121944.1	0.000004246071585	0.0003007745453	235.2587939	-3.332811094	0.7246881378	-4.598959084

ZNF165	0.0000003410667578	0.00003837764607	201.7461338	-3.979801124	0.7804734444	-5.099214012
IER3	0.000000001557226924	0.0000004603895599	3323.173394	-3.640857528	0.6029582449	-6.038324475
TCF19	0.0005481240343	0.01449932314	32.94049168	2.750959286	0.7959802957	3.456064554
SLC44A4	0.0005847884887	0.01522874064	92.84298423	-3.597711151	1.046280239	-3.438573164
HLA-DQA1	0.00005037783971	0.002378961091	110.1365709	4.183135107	1.031887574	4.053867119
PSMB9	0.000005534779214	0.0003759162207	499.4562349	3.754758297	0.8264160199	4.5434239
HLA-DPB1	0.0003465159717	0.01024464279	471.8925206	3.540086196	0.9894626033	3.577786754
KIFC1	0.000523302218	0.01411511779	25.84605592	3.574983092	1.030689314	3.468536098
CLPSL1	0.00001017143755	0.0006259793281	84.91185804	4.408480482	0.9988634881	4.413496474
CDKN1A	0.00002455212022	0.00128988456	348.0610941	-3.058164947	0.7248763847	-4.218877882
MOCS1	0.00045012198	0.01240756712	60.87320929	-3.992887762	1.137961747	-3.508806664
AL133334.1	0.0002479285634	0.008158784206	19.67346425	3.830792398	1.045410609	3.664390206
ME1	0.0002774852859	0.008808259242	76.17489236	3.047218569	0.8381934481	3.63545978
BACH2	0.00000001833272091	0.000003498997037	334.4770537	-3.623201758	0.6438911294	-5.627040959
CALHM6	0.0006401344133	0.01621322536	29.34278079	4.377443983	1.282198851	3.41401334
MAN1A1	0.000005817023179	0.0003933142399	207.3545474	-3.499094002	0.7719271151	-4.53293314

ENPP1	0.0004236739632	0.01190980527	87.10094651	-3.017628885	0.8560939685	-3.524880441
AL355304.1	0.0002492375123	0.008158784206	21.85508225	-3.399303557	0.928000122	-3.663042145
STX11	0.00001948392199	0.001076111999	68.51603101	-3.972940473	0.9302732433	-4.270724222
UST	0.00002621885935	0.001363199866	75.00057224	-3.340000123	0.7944732677	-4.204043432
CCDC170	0.00000008999819615	0.00001244947524	72.42581734	-4.521553623	0.8458077294	-5.345840982
ESR1	0.000000000176824866	0.00000007016224553	360.3002019	-5.626553062	0.8818733942	-6.380227706
AL078604.4	0.00005140845286	0.002407256684	293.2977521	-3.472568215	0.8576085441	-4.049129687
PACRG	0.0001731291934	0.006230171786	28.53873686	-3.362422055	0.8953787627	-3.75530691
SMOC2	0.00001725480988	0.0009780752756	34.79661057	-3.690084871	0.8586104674	-4.297740374
SDK1	0.00000002742046212	0.000004919336746	69.71728176	-3.906710011	0.7030051953	-5.557156672
AGR2	0.00001372793051	0.0008117244557	962.1850222	-3.374151346	0.7759937491	-4.348168204
AGR3	0	0	732.2535285	-7.011580281	0.6771589494	-10.35440835
AC005062.1	0.00002023524988	0.001105460499	154.4707464	-3.769326583	0.8843453239	-4.262279091

MACC1	0.000003167672461	0.0002376227133	405.3892089	-3.368003387	0.7228037858	-4.659637171
CPVL	0.0002801663196	0.008874680183	18.62745671	4.211641564	1.159280016	3.632980389
GLI3	0.00000006344741379	0.00000947188223	189.0805686	-4.122033418	0.7620971787	-5.408802884
CLIP2	0.0003846013421	0.01113055477	64.22740413	-2.736448044	0.770736921	-3.550430724
MAGI2	0.00007060613503	0.003121992094	87.30193038	-4.297068637	1.081231927	-3.974233954
CACNA2D1	0.00008947455146	0.003706311228	97.70683199	-3.248420077	0.8292088516	-3.917493247
PDK4	0.0007842283273	0.01873409991	221.2413383	-2.631102685	0.783462163	-3.358302173
GJC3	0.00005218096647	0.002421974821	84.44191375	-2.87288406	0.7101190324	-4.045637321
IMPDH1	0.00005236528662	0.002421974821	310.996123	2.936942539	0.7261012508	4.044811293
HILPDA	0.00000005088840799	0.00000825048834	287.7247557	4.272191368	0.7841504705	5.448178033
PTN	0.0000000008307249748	0.0000002609514827	231.2969017	-5.349684854	0.8714342312	-6.138942748
CLEC2L	0.0006626152263	0.01659619997	13.22091958	4.399489402	1.292219938	3.404597988
MGAM2	0.0004854298964	0.01325962315	24.94336263	-4.083981181	1.170641274	-3.488670076
MTRNR2L6	0.00046493885	0.01274608724	20.90570295	-4.35862746	1.245257021	-3.500183005

DLC1	0.00002425034671	0.001282970975	97.1644907	-4.274353689	1.012480352	-4.221665812
AC106845.1	0.0001951102143	0.006813952217	19.49000603	-5.635385967	1.512747711	-3.72526491
MICU3	0.000331035449	0.009923166005	17.40146047	-3.691445402	1.028338451	-3.589718345
ZDHHC2	0.0000197055387	0.001080436773	41.538978	-3.40313887	0.7973238745	-4.26820139
SLC7A2	0	0	267.7483742	-6.67646754	0.8334000985	-8.011119212
CLU	0.00003673435487	0.001818948753	1201.992487	-2.962941534	0.7179208497	-4.127114479
RAB11FIP1	0.0000005956181579	0.00006109340534	766.1223434	-2.882244034	0.5772994541	-4.992632529
FGFR1	0	0.000000002745239591	320.9615531	-4.937478854	0.7137172251	-6.917976308
TCIM	0.0000005399587125	0.00005693354872	1441.413436	-4.484019307	0.894738623	-5.011541015
PLAT	0	0.0000000001177465688	771.7724498	-5.812518979	0.7866866314	-7.388607798
CEBPD	0.00001100386392	0.0006636650405	2282.383679	-3.386026387	0.7701738597	-4.396444186
HNF4G	0.00006723264979	0.003008112444	32.65610842	-3.300880426	0.8281450408	-3.985872358
AC083837.1	0.00001427108692	0.000837274119	294.0131846	-3.591472384	0.8275952464	-4.339648396

FABP5	0.0004081487949	0.01158958104	367.8995617	2.932455225	0.8296056586	3.534757984
CTHRC1	0.0007586390802	0.01836077055	107.8273129	3.29552114	0.9786369261	3.367460446
AARD	0.0000006218327482	0.00006292613541	246.2011793	3.106440401	0.6232438036	4.984310126
CDKN2A	0.000000006013575613	0.000001373828683	486.6573694	5.076963546	0.8728734606	5.816379779
FAM189A2	0.00001812218952	0.001012023606	64.64919383	-3.605237914	0.8409986706	-4.286853285
PSAT1	0.0000000001387917617	0.00000005655951846	124.4712215	4.987846177	0.7772605104	6.417212904
SUSD3	0.0000000685564167	0.00001001607637	108.2016152	-4.323505121	0.8014039143	-5.394913906
KLF4	0.0002327436443	0.007763957233	436.916457	-2.76037967	0.74999381	-3.680536604
ZNF483	0.000007772411267	0.0005029717472	46.233311	-3.394107239	0.7590773346	-4.471358958
UGCG	0.0000001846458815	0.00002379564617	347.0958775	-3.35502415	0.6434440391	-5.214166185
ORM2	0.0003659911844	0.0107571444	39.65565919	5.51656633	1.548090402	3.563465236
ASTN2	0.0002177108692	0.007410032699	88.24659299	-3.025339268	0.8182063393	-3.697526067
BRINP1	0.0000108366478	0.0006562047211	78.03594633	-4.630703639	1.052488048	-4.399768385
STOM	0.000004248904241	0.0003007745453	412.8070108	-2.528193722	0.5497483378	-4.598820129
PHYHD1	0	0.000000002434330656	125.2549778	-4.484919616	0.6460860901	-6.941674933

SH3GLB2	0.00003399652459	0.001720132878	118.4904174	-2.963963376	0.715087197	-4.144897837
RALGDS	0.00006527172309	0.002929068574	202.7900804	-2.549713568	0.6385627822	-3.992894103
KLF6	0.000002832144395	0.000216766869	2952.958464	-2.590668832	0.5532505502	-4.682632183
MANCR	0.000004633509064	0.0003204772921	30.32045192	-4.587382855	1.001451881	-4.580732175
CALML5	0.0000002599913807	0.00003083591982	2417.719773	4.573758249	0.8880468731	5.150356796
SFMBT2	0.000002492263788	0.0001937028525	214.0032521	-3.394839068	0.7209621912	-4.708761582
GATA3-AS1	0.00001734633088	0.0009795804381	34.2522833	-3.706144224	0.862582604	-4.296567316
FAM107B	0.000003985487426	0.0002889095164	473.2591749	-2.863802586	0.6209273765	-4.612137739
ST8SIA6-AS1	0.000000002178635262	0.0000006083233792	203.2942737	4.877602768	0.8151216905	5.983895196
CACNB2	0.0004324532792	0.01210997109	114.0497413	-3.503643093	0.9955105632	-3.519443412
OTUD1	0.000003072647482	0.0002316468937	38.33577853	-3.594710502	0.7704211939	-4.665902925
ANKRD30A	0	0	3072.994567	-9.044667973	1.043082911	-8.671092088
FRMPD2	0.0003021081972	0.009251137636	20.74417778	-4.502611891	1.246057809	-3.613485552
MAPK8	0.00010410341	0.00417465749	670.3228115	-2.592471313	0.6680209425	-3.880823412

LNCAROD	0.00000001582087735	0.000003098015437	54.80701207	6.828055629	1.207989024	5.652415291
CDK1	0.00000001497017889	0.000003009604765	99.3167555	4.051561344	0.7155827266	5.661904897
TMEM26	0.00000001799696728	0.000003478952214	33.95010444	-7.127576829	1.265947764	-5.630229802
DNAJC12	0.000000000751009128	0.0000002516381252	365.9163134	-5.379792186	0.874059364	-6.154950576
CFAP70	0.0004812887523	0.01317036626	44.75404678	-2.773009028	0.794340017	-3.490959751
KCNMA1	0.0000001749337977	0.0000227383776	283.6962637	-4.11123724	0.786964003	-5.224174453
DLG5	0.0000003347639992	0.00003795166601	274.6923866	-2.934079502	0.5750003519	-5.102743836
ADIRF	0.00001962583311	0.001079993838	1013.463353	-3.196011221	0.7486371879	-4.269105614
MYOF	0.000004440502468	0.0003114134707	613.0653373	-3.238207814	0.7055500634	-4.589621604
DNMBP	0.00006960658087	0.00308720819	67.82284725	-3.67178783	0.923110249	-3.977626544
BTRC	0.000004882379754	0.0003331064341	206.3438622	-3.20501079	0.7013491338	-4.569779352
AC010789.1	0.00000001209968865	0.000002553277235	29.94693866	7.209651939	1.265225095	5.698315633
DUSP5	0.000000003732293519	0.0000009076697046	346.5572594	-4.90062514	0.8312285353	-5.895641128
TDRD1	0.000557314756	0.01465961025	9.089570361	7.048874815	2.042216914	3.451579882

GFRA1	0.0000002861565523	0.00003318975766	53.88810411	-5.42832011	1.057668889	-5.132343558
AC063960.1	0.0006596208988	0.01654869203	14.34248711	4.419707491	1.297687	3.40583476
CHST15	0.0003428911372	0.01015739207	48.46356477	-2.661081749	0.7432078155	-3.580535206
PTPRE	0.0002588934722	0.008412921928	304.6666991	-3.140185254	0.8595480725	-3.653298
MKI67	0.000000002527392384	0.0000006685617958	76.64774459	5.236020906	0.8785748267	5.959675541
DUSP8	0.0007047470282	0.01739144958	79.40909737	-2.893692872	0.8541697634	-3.387725715
ASCL2	0.0005350489181	0.01435492453	31.39281329	5.255091666	1.517686689	3.462566882
CDKN1C	0.0008187873391	0.01932030594	119.7946036	-3.363558269	1.005138093	-3.346364339
SYT9	0.000004117150418	0.0002943492211	24.42586187	-4.43008537	0.9619371676	-4.605379144
RIC3	0.00008013222782	0.003413089636	95.58247277	-2.563978293	0.6500952112	-3.944004277
NRIP3	0.0003024570868	0.009251137636	28.34719486	-3.369256081	0.9324888591	-3.613186418
SCUBE2	0.0000009251473813	0.0000861072359	82.17660667	-4.190330005	0.8539622728	-4.906926382
DENND5A	0.0000001600858343	0.00002098934095	201.9942222	-3.331515912	0.6357169544	-5.240564828
WEE1	0.00003745958737	0.001845802805	308.4399638	-2.635251832	0.6392185498	-4.122614766

MTRNR2L8	0.00000002686563139	0.000004880481809	522.1744618	-5.139667812	0.9242801711	-5.560724954
MICAL2	0.00008094330331	0.003437924302	151.7135552	-2.805522421	0.7117743168	-3.941589848
ABCC8	0.0003310145204	0.009923166005	16.19755662	-4.016108527	1.118775821	-3.589734827
FADS2	0.0000002638170617	0.00003083591982	136.5964317	4.132771527	0.8028514696	5.147616568
SAC3D1	0.0000001884502928	0.00002387776062	119.3416859	2.970428657	0.5700978512	5.210383886
CDCA5	0.0001958266074	0.006819107591	56.52445657	3.254822567	0.8739326378	3.724340328
UCP2	0.00004490004264	0.002176858016	566.03241	3.099098171	0.7594523621	4.080701208
SYTL2	0	0	672.0979932	-5.962724662	0.6204265589	-9.610685708
PRSS23	0.00007294100788	0.003178625771	266.2514001	-2.980274423	0.7513644966	-3.966482894
CTSC	0.00007111043606	0.00312595672	440.8070696	3.688366893	0.9284657538	3.972539512
PGR	0	0	219.2465479	-6.629345072	0.8616463528	-7.693812027
ZBTB16	0.00004220695189	0.002066222145	71.15107918	-3.611581777	0.8819374651	-4.095054264
AP002518.2	0.000147162104	0.005478790628	40.36563915	-4.143608516	1.091626921	-3.795810122
AP000462.1	0.0001309105652	0.004971963482	13.44577288	-4.495856134	1.175467356	-3.824739249
JHY	0.0003380018363	0.01008951439	23.91219617	-3.451276615	0.9628910248	-3.584285787

VWA5A	0.0003032727279	0.009256571238	61.50224029	-2.681717532	0.7423463461	-3.612488357
VSIG2	0.0000007217958502	0.0000702144376	65.5040028	-4.868849254	0.9825329576	-4.955405533
TMEM45B	0.000000002114326228	0.0000006015058655	124.7544129	-5.861221673	0.978701921	-5.988770991
GPR162	0.0004448172102	0.01229288802	14.45069871	-5.254830589	1.496267787	-3.511958645
A2ML1	0.000009945630092	0.0006171202079	41.05376376	5.466229922	1.237164851	4.418352104
CLEC7A	0.000001259325584	0.0001072774642	54.16833581	3.733072593	0.7703302603	4.8460677
AC024901.1	0.0000002619041203	0.00003083591982	73.77444942	6.217343844	1.207489935	5.148981922
LINC02398	0.0002703060216	0.008690136874	68.11159995	7.659865593	2.103081041	3.642211328
KCNJ8	0.000045447366	0.002196331361	16.14579315	-5.369497879	1.316736192	-4.077884327
SOX5	0.0003209579065	0.009711174135	123.2981654	-4.144988274	1.152099465	-3.597769463
PTHLH	0.000008004309675	0.0005135701331	684.7275508	-8.061182402	1.805388249	-4.465068611
AC008011.2	0.00000902957825	0.0005649293812	15.79881716	-5.727896215	1.29030052	-4.439195465
ANO6	0.000255682237	0.008326515701	363.5592896	-2.56595733	0.7017522786	-3.656500175
RND1	0.00000004593085609	0.000007527667915	304.5703	-4.277424872	0.782496219	-5.466384077
TROAP	0.0000009523353198	0.00008704037934	44.51824279	4.260947833	0.869361154	4.901240196

LINC02381	0.0003206864654	0.009711174135	27.31044764	-3.140897672	0.8729590801	-3.597989578
LYZ	0.0000005216114395	0.00005617755203	47.75554906	5.095041794	1.015315066	5.018187912
KCNC2	0	0.00000001117905704	78.74085571	-7.270235549	1.087419718	-6.685767629
AC078923.1	0.00002309281905	0.001234083558	69.78167155	-3.532922461	0.8346784333	-4.232674909
NAV3	0	0	417.8974071	-6.417170766	0.8363484791	-7.67284323
AC027288.3	0.0000009502640506	0.00008704037934	131.4761334	-3.881118311	0.7917954514	-4.901667854
LIN7A	0.0000006856044069	0.00006756564214	31.74116662	-4.733097768	0.9532162566	-4.965397658
NTN4	0	0.0000000001177465688	809.0439032	-4.906771739	0.6640454892	-7.389210255
ALDH1L2	0.0006700752756	0.01672747517	32.14379657	3.026830659	0.8898414853	3.40153916
TBX3	0	0.00000001555576025	287.8812307	-3.687815113	0.5560252949	-6.632459254
CLIP1	0.00007922794241	0.003393746919	751.3818451	-2.580005411	0.6537081632	-3.946723563
HCAR2	0.000001052328681	0.0000933353638	48.9764267	-4.6226363	0.946952635	-4.881591887
LINC00621	0.000688307739	0.01706957909	18.23112617	-5.479695931	1.614431889	-3.394194558
DCLK1	0.0001238482166	0.004727552937	106.2119211	-4.039649765	1.052435852	-3.838380988

FOXO1	0.00009796546365	0.003970761454	205.0876914	-2.760405075	0.708599902	-3.895576428
TNFSF11	0.0002490345067	0.008158784206	11.04904973	-5.737274776	1.566170355	-3.663250781
LPAR6	0.0006550858332	0.01647432334	63.87842972	-2.760585952	0.8100981477	-3.407717892
INTS6	0.0005039055536	0.01367791337	1133.011528	-3.322522546	0.9551124265	-3.478671676
ITGBL1	0.000003743969735	0.0002767234101	45.24614579	-3.922461915	0.8480794312	-4.625111482
GAS6	0.000162944585	0.00596889872	103.1195236	-4.085316445	1.083505082	-3.770463575
ANG	0.00000001279523412	0.000002642829316	83.74744454	-3.876478003	0.6814255373	-5.688777116
RNASE4	0	0	251.8404723	-4.640943862	0.6197314082	-7.488637499
SLC7A8	0.000001451278544	0.0001208971154	94.30508034	-4.052244789	0.8410933332	-4.817830114
SLC22A17	0.0000001155106431	0.00001555062032	48.14258422	-3.926293437	0.7407456525	-5.300460994
NOVA1	0	0.000000002434330656	192.9581021	-6.839698774	0.984877512	-6.944720222
PRKD1	0.000000003487981245	0.0000008796237095	73.15317324	-5.186483994	0.878051779	-5.906808822
FRMD6	0.00000638783638	0.0004283540453	179.0526172	-3.000077351	0.6647445355	-4.513128263

SAMD4A	0.00004710906388	0.002269362509	403.6919845	-3.576048154	0.8787384434	-4.069525103
RTN1	0.000596807779	0.0155149443	17.50154236	-3.446234325	1.003837191	-3.433061014
SIX1	0.0004976950621	0.01357006536	26.86055257	3.922983108	1.126648478	3.481993882
AL136038.5	0.0006898459317	0.01707963376	42.56202524	-2.512119986	0.7402558945	-3.393583226
ARG2	0.000272354239	0.008730739613	159.5115635	-2.616093319	0.7186540114	-3.640268164
SLC10A1	0.0006367292334	0.01618988766	51.30026749	-3.899478921	1.14171204	-3.415466233
TTC9	0.0003597770092	0.010615886	80.76463854	-3.239697376	0.9079981502	-3.567955921
RGS6	0.000001070464359	0.00009406846376	97.10472874	-3.978858287	0.8156369956	-4.87822194
ELMSAN1	0.0003323906693	0.009944020857	153.5420981	-2.724672531	0.759246545	-3.588653184
BBOF1	0.00002180612789	0.001178468804	62.78794516	-2.731267179	0.6433252302	-4.2455465
FOS	0.0007757006793	0.01862422746	5180.923553	-2.778536272	0.8266198483	-3.361322956
BATF	0	0	554.0219096	-4.465180654	0.5937372702	-7.520465496
TGFB3	0.000006392071906	0.0004283540453	36.77601599	-3.327315267	0.7372754947	-4.512987738

NRXN3	0.0006526578256	0.01645614497	106.7751995	-3.494938963	1.025290315	-3.408731082
DIO2-AS1	0.00000005352527394	0.000008468297897	132.6154824	-5.403117624	0.9933692297	-5.439183601
STON2	0.000001073071745	0.00009406846376	40.60594064	-3.941354958	0.8080285894	-4.877741963
SERPINA1	0.000008366373024	0.0005278166212	218.5945789	-4.201328971	0.942934886	-4.455587585
SERPINA11	0.0000002980779554	0.00003430854513	23.25325044	-7.156705399	1.396523455	-5.124658218
SERPINA5	0	0.000000003555165443	76.49419125	-5.773389397	0.8402295664	-6.871204761
SERPINA3	0.0002171728724	0.007408444728	4105.875609	-3.488928749	0.9434243336	-3.698154293
EVL	0.00000001460851743	0.000002976584132	412.8825129	-3.704773337	0.6538490348	-5.666098961
WARS	0.0001182709608	0.004584291894	275.9685251	2.842835922	0.7384598108	3.849682651
MEG3	0.00009415198461	0.003847218493	34.01920082	-3.96731425	1.015908527	-3.905188453
IGHG4	0.000000009465960123	0.000002044525024	42.63295942	7.77855746	1.355142225	5.740030321
MKRN3	0.0000008313825565	0.00007984449801	33.17140714	6.470468523	1.313040165	4.9278527
ARHGAP11B	0.0006555640009	0.01647432334	21.70406728	4.259360725	1.249988926	3.407518768
ARHGAP11A	0.0008430475338	0.01970770653	22.75500877	3.771955262	1.129916483	3.338260232
AC013652.1	0.0002626679005	0.008498941209	59.66964307	-2.9979283	0.8214444053	-3.649581494

FSIP1	0.000004281664338	0.0003016772658	42.2959558	-3.92447198	0.85366208	-4.597219522
PHGR1	0	0	63.78749903	-8.954877322	1.106601553	-8.092232741
KNSTRN	0.0001659637278	0.006029882141	58.9454161	2.752913722	0.7310147905	3.765879648
NUSAP1	0.00001834611444	0.00102074802	124.8065729	2.695144243	0.629100529	4.284123314
C15orf48	0.000004572167255	0.0003176918796	620.6519017	-3.433331275	0.7490602214	-4.583518356
AC025580.2	0.0000003611629525	0.00004033788887	223.1530711	-4.142449445	0.8141021935	-5.088365415
AC104574.2	0.0004230022631	0.01190980527	19.75970156	-4.326693296	1.227326009	-3.525300746
PCLAF	0.00002655204709	0.00136638828	213.5735755	2.9557006	0.7035395925	4.201185878
DENND4A	0.0000107322281	0.0006525021587	529.4647922	-2.687729726	0.6105882837	-4.401869144
SMAD3	0.0001367797108	0.0051559112	319.3112164	-3.125855472	0.8195912336	-3.813920091
KIF23	0.0000007440563079	0.00007191590392	75.70269179	3.792005067	0.7661395249	4.949496722
THSD4	0.0000001332457526	0.00001777946423	1176.468702	-4.132009208	0.7834195614	-5.274324783
PEAK1	0.0006480575062	0.01636752274	363.6621216	-2.802281388	0.821624275	-3.410660412
RASGRF1	0.00001597352859	0.0009211448435	18.94518935	4.657409671	1.07939916	4.314816839
MESP1	0.0000158474815	0.0009190320234	90.65152814	2.829424832	0.6554803929	4.316566692

IGF1R	0	0.000000006899646796	634.6652814	-3.567214723	0.5268575949	-6.770737971
ALDH1A3	0.00001904115314	0.001055523923	283.0846651	-3.687802286	0.8624728268	-4.275847507
AC015712.2	0.00002739124824	0.001404779731	147.9246661	-3.860216916	0.9203840921	-4.194136936
MCRIP2	0.00003375246083	0.001713534022	294.9766262	2.633658196	0.6351446636	4.146548569
HAGHL	0.000009591645086	0.0005976149777	97.76488351	3.263350214	0.7372836485	4.426180102
MSLN	0.000004119093092	0.0002943492211	123.9989626	8.507728799	1.84738539	4.60528098
RPUSD1	0.000199345311	0.006893872933	123.0052827	2.765863422	0.7435427125	3.719844705
TEDC2	0.000002296817712	0.0001803719659	33.32225155	4.05740132	0.85863963	4.725383244
PKMYT1	0.0001361723453	0.005145881258	34.51455039	3.405543817	0.8926675432	3.8150192
PAQR4	0.0000009087110504	0.00008510276533	76.01979108	3.474344258	0.7075419915	4.910442489
C16orf89	0.00000005341085777	0.000008468297897	84.71218518	-4.216448492	0.7751444442	-5.439564875
RBFOX1	0.0001192686605	0.004602315102	58.06126249	-3.436115148	0.8930484587	-3.847624521
RMI2	0.0007618207606	0.01836402165	36.0176373	2.830491817	0.8408301559	3.366306259
BMERB1	0.0003234713899	0.009753306069	158.4045523	-2.53230738	0.7042521825	-3.595739485
XYLT1	0.00004739434496	0.002275834183	106.3913573	-3.10236042	0.7626033185	-4.068118174
TMC5	0.000002464657152	0.0001925497437	343.8574748	-3.713077139	0.7881664272	-4.711031846

ACSM1	0.0002289482791	0.007662077769	12.96849182	-4.079818939	1.107224335	-3.684726581
PLK1	0.000000005017258352	0.00000118203471	86.47438637	5.698985822	0.974752345	5.846598729
HS3ST4	0.0000007087091648	0.00006938907005	33.62505789	-4.392355625	0.8857409763	-4.958961754
IL4R	0.0000363058008	0.001810015199	89.92283397	-3.350837428	0.8113774687	-4.129813259
SHCBP1	0.0003240752315	0.009753306069	24.75736249	4.13859322	1.151126796	3.595254001
MT1M	0.000292943281	0.009069812712	63.97674905	-3.969774067	1.096180077	-3.62146161
CX3CL1	0.0007315431572	0.01784823256	385.5908896	-2.6838615	0.7946352358	-3.377476079
AC009107.2	0.0008243799517	0.01939157709	48.06976829	-2.65444951	0.7936816005	-3.344476561
RRAD	0.000004827111311	0.0003308669289	161.957699	-4.089807221	0.8945012777	-4.572164762
TAT	0.00007099780169	0.00312595672	13.27125385	-6.19475773	1.559246713	-3.972916973
COTL1	0.000229181395	0.007662077769	547.1844968	3.614001638	0.9808749243	3.684467355
RPH3AL	0.00005998686034	0.002724343013	30.32035979	-3.890784501	0.9695783267	-4.012862493
MIR22HG	0.000007915753961	0.0005100587104	121.5970085	-3.287510187	0.7358807053	-4.467449905
SLC16A11	0.0002229756063	0.007538175318	14.5974523	-3.72846034	1.010024831	-3.691454138

ALOXE3	0.0001818851707	0.006452857892	16.0214403	-4.515376978	1.206374172	-3.742932403
AURKB	0.00002267527144	0.001220120982	40.92915279	3.768922549	0.8895733568	4.236775438
RASD1	0.000001005891278	0.00009027874222	205.6771547	-4.101184366	0.8386052723	-4.890482449
TOM1L2	0.000137315616	0.005163204135	284.2309846	-2.685212225	0.7042340786	-3.81295411
MTRNR2L1	0.0000005285920954	0.00005652561429	72.85323171	-5.123593643	1.021524878	-5.01563276
KSR1	0.0001152168507	0.004492056319	39.67726368	-2.785809176	0.7224447265	-3.856086249
SLC13A2	0.0007475778008	0.01812215126	21.02588412	-4.16058457	1.234042504	-3.371508322
CCL2	0.0005449456136	0.01447481223	536.8794785	-3.341384655	0.966379548	-3.457631798
CCL14	0.0002064819364	0.007091878444	20.93834524	-5.728348206	1.54363341	-3.710951168
PSMD3	0.0005369872592	0.01438133907	1101.206605	3.126878232	0.9033059766	3.461593649
IGFBP4	0.00002564752369	0.00133810852	500.1996424	-4.069130379	0.9667632219	-4.209024803
KRT23	0.00005048862907	0.002378961091	684.7853104	-3.819813944	0.9423836275	-4.053353467
ZNF385C	0.00005182558794	0.002419276207	53.98984255	-3.747225495	0.9258723351	-4.047237781
STAT5B	0.000006851857694	0.000453124168	160.5889234	-2.880683631	0.6404024983	-4.498239214

WNK4	0.0001948813317	0.006813952217	15.9187351	-4.409873544	1.183680409	-3.72556098
PLCD3	0.0003939375242	0.0113138857	36.79963219	-2.935712358	0.8283350445	-3.544112225
MAPT	0.000004827611378	0.0003308669289	157.7327084	-3.471357129	0.7592407066	-4.572143062
SKAP1	0.0003766012864	0.01098335435	50.24106557	-2.812909699	0.7910408575	-3.555960064
SEPTIN4-AS1	0.0005398430131	0.01440664239	29.35438326	-3.491297737	1.008997258	-3.460165734
TANC2	0.00000004034518566	0.000006759163437	265.8937272	-3.734636218	0.6803441275	-5.489334099
AC015923.1	0.00008502906262	0.003561300573	23.0798917	-3.671143934	0.9341892726	-3.929764601
LIMD2	0.0008046755556	0.01901708155	57.50720858	3.579365579	1.068090649	3.351181459
CACNG4	0.0000005515773571	0.00005775474577	49.27076791	-4.745982047	0.9477854416	-5.007443498
ABCA10	0.0002895407494	0.009038706873	35.51426795	-3.861997127	1.065530665	-3.624482388
TK1	0.0000000006227072526	0.0000002183530222	324.1615957	4.111520624	0.6648026487	6.18457317
BIRC5	0.00000007191838041	0.00001032747943	212.5339938	4.14755079	0.7700169191	5.386311245
SOCS3	0.00004356032225	0.002118717867	475.0192067	-3.017761443	0.7382476148	-4.087736124
CBX2	0.0001049130785	0.004195966574	14.30417787	5.247226691	1.352747945	3.878938949
PCYT2	0.00007549049663	0.00326144902	112.3292899	2.591543069	0.6547139504	3.958282953

PYCR1	0.00000006771848699	0.00001001038575	364.0266162	2.840030733	0.5262121058	5.39712162
NDC80	0.0006325606518	0.01613031062	31.58511591	3.823662551	1.118928164	3.41725472
PTPRM	0.0000000155264057	0.000003080357172	111.660205	-3.718771444	0.6575329387	-5.655642821
RAB31	0.000001720160348	0.0001409596616	204.4831081	-2.749847728	0.5748255657	-4.783795105
ANKRD30B	0.00002316259762	0.001234083558	22.44790553	-4.771351662	1.127446968	-4.231996537
DTNA	0	0.000000007771313109	577.5121994	-4.949163897	0.733381594	-6.748415746
SLC39A6	0.000000002720604098	0.0000007072632515	1096.927706	-3.22466286	0.5421765276	-5.947625351
SKA1	0.0005580724422	0.01465961025	10.35542734	4.908811252	1.422343653	3.451213244
NEDD4L	0.0000000007932418104	0.0000002544787238	1146.696067	-3.021073738	0.4915292923	-6.146274057
DSEL	0.00001809312205	0.001012023606	36.66565822	-3.935843627	0.9180431128	-4.287210014
ZNF516	0.00000005886761699	0.000008965716455	60.71781622	-3.812357641	0.7031007038	-5.422207117
NFATC1	0.000003976860181	0.0002889095164	109.6535194	-3.881799853	0.8415665615	-4.612588036
SHC2	0.000000843553175	0.00008050059982	26.88854537	-4.494822367	0.9126521243	-4.925011675
MISP	0.000075864412	0.003268238869	59.69256556	-3.864273237	0.9765411245	-3.95710241

PLPPR3	0.00005328471873	0.002449472527	35.92444939	-4.730433593	1.170687412	-4.040731579
CFD	0.00000009536527021	0.00001307197767	323.8109287	-5.110960111	0.9579444509	-5.335340798
GADD45B	0.000000256882742	0.00003083591982	1764.573781	-3.20322975	0.6216710256	-5.152612263
GNA15	0.00009045558541	0.003726473543	113.077875	-3.157866678	0.8066353137	-3.914862918
INSR	0.000000005803281405	0.000001346182723	508.8249301	-3.412017642	0.5860227152	-5.822330011
ICAM1	0.0001894900151	0.006675538429	401.2034333	-2.823374233	0.7564032335	-3.7326311
RGL3	0.0003863476594	0.01115967435	44.20476249	-3.504789347	0.9874765537	-3.549238039
CYP4F22	0.000008877965126	0.000557758159	29.6014554	-5.311137864	1.195437876	-4.442838872
JUND	0.0003128250584	0.009528840868	1881.496845	-3.163930979	0.8777867793	-3.604441367
GDF15	0.00002177999983	0.001178468804	87.41890062	-3.517711817	0.8285126819	-4.245815295
HAMP	0.0003873232044	0.01116646133	18.75095366	-5.152896776	1.45210353	-3.548573962
DMKN	0.0001024652627	0.004130939122	190.2332477	-2.753213774	0.7087365307	-3.884678799
IGFLR1	0.0002820282516	0.00887980474	32.52982824	2.993155192	0.8242719273	3.631271542

COX7A1	0.00000123246145	0.0001055855326	24.07340103	-4.546651472	0.9373869924	-4.850346237
KCNK6	0.00005760456761	0.002624053385	145.9694236	-3.185477781	0.7919317566	-4.022414501
AXL	0.0002763105737	0.008789474325	52.53479402	-3.746165116	1.030141699	-3.636553222
TGFB1	0.00007393367315	0.003212599204	111.3050214	-2.717046292	0.6855586991	-3.963258428
BCAM	0.000000001031347447	0.0000003173603431	477.6070426	-3.338739435	0.5469324911	-6.104481795
IGFL2	0.000171264115	0.006177799821	14.92673587	-6.268331517	1.667988398	-3.758018656
MEIS3	0.0000001959727213	0.00002462397243	36.02138217	-4.565507665	0.8774561669	-5.203117645
PRKCG	0.00008362481351	0.0035220529	65.76772575	-3.982522664	1.012394114	-3.933767106
CDC42EP5	0.000000560927718	0.00005832874574	101.6794645	-4.755231241	0.950246916	-5.004205919
TMEM238	0.0000006307203132	0.0000629866818	192.234898	-4.997106545	1.003119775	-4.981565183
EBF4	0.00000005536661244	0.000008606368891	33.5988289	-4.759524983	0.8760151216	-5.43315391
C20orf27	0.0001161313352	0.004512959463	200.9564729	3.018685726	0.7832295049	3.854152209
BPIFB1	0.00001036994471	0.0006356017329	31.81934294	-5.281544081	1.197815863	-4.409312186

TGM2	0.0002910973283	0.009049825807	114.5496405	-3.512034152	0.9693460551	-3.623096348
PREX1	0.000000009491759627	0.000002044525024	74.12430567	-4.497774925	0.7836432723	-5.739569372
COL9A3	0.00000001219233061	0.000002553277235	65.06591417	4.701846358	0.8253175524	5.697014858
EEF1A2	0.00002345467673	0.001245245126	78.0266256	-5.365780761	1.268752656	-4.229177954
NCAM2	0.000001778886774	0.0001449840799	69.70525821	-4.830988767	1.011291977	-4.777046468
GRIK1	0.000000001767267316	0.0000005124395497	119.138855	-5.773190267	0.9593410968	-6.017870271
GRIK1-AS1	0.000001543802075	0.0001278980642	23.44232475	-7.025191795	1.461912424	-4.805480601
RCAN1	0.00003600716008	0.001803982673	626.5739422	-3.027473976	0.732740829	-4.131711864
AP000331.1	0.0004181065718	0.01180563837	131.2912209	-2.816541896	0.7982528584	-3.528383101
ETS2	0.000250557988	0.008177301607	337.2988843	-2.586884743	0.7064731049	-3.661688924
AP001042.1	0.000006421853416	0.0004284456009	66.90205052	-4.659420811	1.032672558	-4.51200216
TFF3	0.00000002862488635	0.000005018674842	2416.317161	-8.250628638	1.486694622	-5.549645849
TFF1	0.0000005004641516	0.00005428775883	1947.627375	-9.372199014	1.864693124	-5.026134806

AP000547.3	0.00007230155397	0.003159892263	35.48116842	-3.716494932	0.9364792909	-3.96858208
CDC45	0.00001462567387	0.0008547515916	29.70671215	4.154077046	0.9584300363	4.334251734
PRAME	0	0.00000001117905704	87.97889242	8.225285712	1.230282197	6.685690267
GSTT2B	0.0002964336321	0.009159070298	35.99588021	-3.891686313	1.075527782	-3.618396825
GGT5	0.0000547948106	0.002511234512	37.7243704	-3.575401913	0.8862786571	-4.034173546
INPP5J	0.00000008796155585	0.00001228041055	44.38826049	-4.142125206	0.7742311912	-5.349984931
GCAT	0.0001135974569	0.004448889492	232.0076199	2.723581898	0.7056740007	3.859546895
MAFF	0.00008156795358	0.003447015742	837.3143931	-2.68730046	0.6820999425	-3.939745912
CENPM	0.00005099606213	0.002395385124	70.33505974	3.213400419	0.7932335159	4.05101443
LINC01315	0.000000001123496518	0.0000003388016101	222.0005807	3.900946774	0.640465691	6.090797413
AL022316.1	0.0003671065177	0.010768934	19.9603049	3.822023544	1.072798487	3.562666792
TTLL12	0.0001098145975	0.0043345144	102.5454571	2.52643595	0.6531944975	3.867815727
UPK3A	0.000001092473261	0.00009521567529	49.15328162	-5.259525492	1.079052923	-4.874205315
ANOS1	0.00003856188798	0.001893928818	23.6645249	-4.170311602	1.013212108	-4.115931469
PIR	0.0000009524912184	0.00008704037934	72.51116401	3.610174438	0.7365886953	4.901208044
RAI2	0	0.00000002054649156	60.97120481	-5.34499698	0.8114822639	-6.586708322

AL591501.1	0.0000172108553	0.0009780752756	32.18661622	-5.377203041	1.251005212	-4.298305865
SYTL5	0.0003901215856	0.01122567417	18.95750134	-3.996797077	1.126912973	-3.546677671
MAOA	0.00006961472242	0.00308720819	35.49016601	-3.405683618	0.8562159868	-3.977598725
MAOB	0.0001321508236	0.005006457585	94.89800194	-4.38469924	1.147101709	-3.822415401
MTRNR2L10	0.0000000778952686	0.00001108023453	55.62014089	-5.716850093	1.06420644	-5.371937132
FAAH2	0.0002970834454	0.009160376665	309.5585343	-2.872998161	0.7941219406	-3.617829976
CITED1	0.00000003115039684	0.000005398686017	47.58207464	-5.731728291	1.035571627	-5.534844852
ARMCX1	0.00003601265318	0.001803982673	63.20417074	-3.01042815	0.7286214027	-4.131676807
ARMCX2	0.000006946695345	0.0004573898359	44.67036799	-4.164283047	0.9263606272	-4.495315242
TCEAL1	0.0005671422416	0.01484612972	146.8679832	-2.683086342	0.7784146398	-3.446860073
COL4A6	0.000000990580214	0.00008943693692	79.69993429	-4.891451619	0.9995812889	-4.893500582
COL4A5	0.0003009279567	0.009251137636	236.3518969	-3.293644375	0.9112310098	-3.614499879
CT83	0	0.00000002033732928	56.08301255	7.039228681	1.007649069	6.985793862

HS6ST2	0.0000006307858438	0.0000629866818	53.649626	-5.145177725	1.032847769	-4.981545083
VGLL1	0.0001193464123	0.004602315102	44.09776807	4.097867808	1.065082596	3.847464811
HIST1H1B	0.0001201396488	0.004621085776	10.72258112	6.194406181	1.610676615	3.84584101
